# Twisted and Disconnected Chains: Flexible Linear Tetracuprous Arrays and a Decanuclear Cu^I^ Cluster as Blue- and Green/Yellow-Light Emitters

**DOI:** 10.1021/acs.inorgchem.4c01646

**Published:** 2024-06-27

**Authors:** Janet Arras, Alvaro Calderón-Díaz, Sergei Lebedkin, Samer Gozem, Colin D. McMillen, Nattamai Bhuvanesh, Michael Stollenz

**Affiliations:** † Department of Chemistry and Biochemistry, Kennesaw State University, 370 Paulding Avenue NW, MD # 1203, Kennesaw, Georgia 30144, United States; ‡ Institute of Nanotechnology, Karlsruhe Institute of Technology (KIT), Herrmann-von-Helmholtz-Platz 1, 76344 Eggenstein-Leopoldshafen, Germany; § Department of Chemistry, Georgia State University, 145 Piedmont Ave SE, Atlanta, Georgia 30303, United States; ∥ Department of Chemistry, Clemson University, 379 Hunter Laboratories, Clemson, South Carolina 29634-0973, United States; ⊥ Department of Chemistry, Texas A&M University, 580 Ross Street, P.O. Box 30012, College Station, Texas 77842-3012, United States

## Abstract

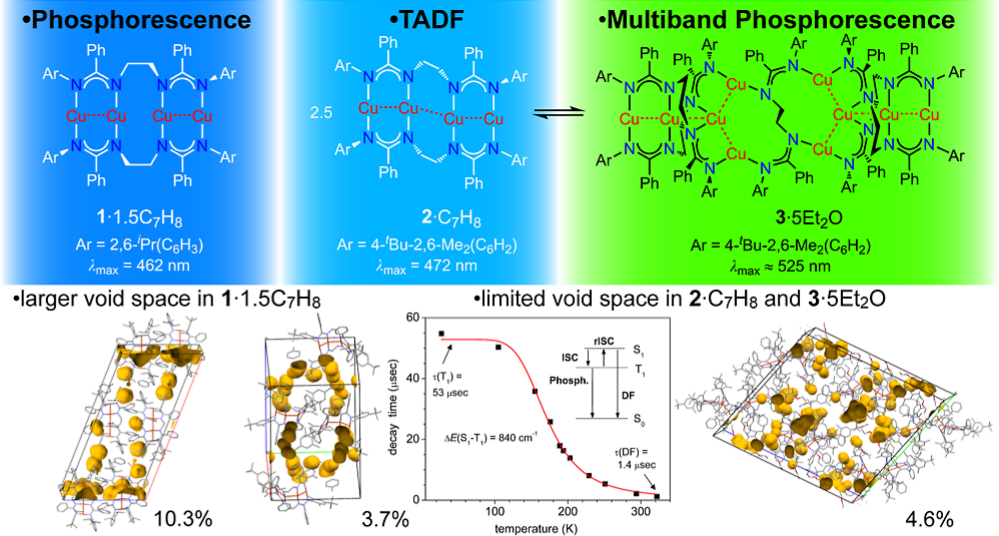

Defined arrays of transition metal ions embedded in tailored polydentate ligand scaffolds allow for a systematic design of their physical properties. Such molecular strings of closed-shell transition metal centers are particularly interesting for Group 11 metal ions in the oxidation state +1 if they undergo metallophilic d^10^···d^10^ contact interactions since these clusters are oftentimes efficient photoluminescence (PL) emitters. Copper is particularly attractive as a sustainable earth-abundant coinage metal source and because of the ability of several Cu^I^ complexes to serve as powerful thermally activated delayed fluorescence (TADF) emitters in molecular/organic light-emitting devices (OLEDs). Our combined synthetic, crystallographic, photophysical, and computational study describes a straight tetracuprous array possessing a centrally disconnected Cu^I^_2_···Cu^I^_2_ chain and a continuous helically bent Cu^I^_4_ complex. This molecular helix undergoes a facile rearrangement in diethyl ether solution, yielding an unprecedented nanosized Cu^I^_10_ cluster (2.9 × 2.0 nm) upon crystallization. All three clusters show either bright blue phosphorescence, TADF, or green/yellow multiband phosphorescence with quantum yields between 6.5 and 67%, which is persistent under hydrostatic pressure up to 30 kbar. Temperature-dependent PL investigations in combination with time-dependent density-functional theory (TD-DFT) calculations and void space analyses of the crystal packings complement a comprehensive correlation between the molecular structures and photoluminescence properties.

## Abstract 2

Subtle modifications on the flexible bis(amidinate) ligand framework of a highly luminescent linear tetranuclear Cu^I^ complex lead to straight and centrally disconnected or helically bent and connected arrangements that interconvert into a unique nanosized Cu^I^_10_ cluster. A combined synthetic, crystallographic, photophysical, and computational study reveals adjustable photoluminescence properties through crystal packing and aggregation states.

## Introduction

**Scheme 1:**
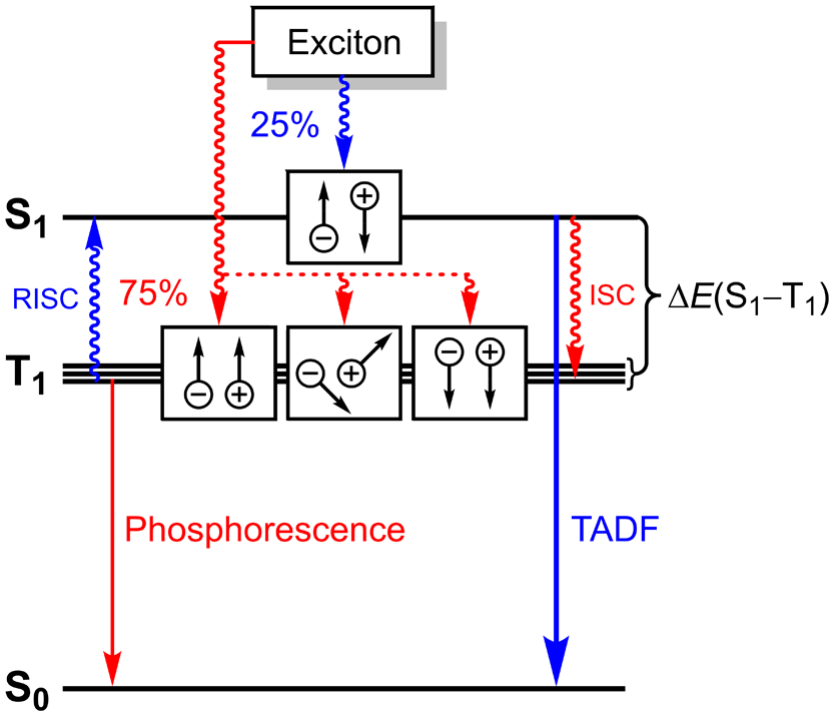
Simplified Mechanism for Phosphorescence and TADF in an OLED Emitting Component Three triplet paths and one singlet path as well as (Phosphorescence) and RISC (TADF) are represented. Spin states and electron–hole recombinations are shown in boxes.

Discrete linear arrangements of transition metal ions in designed ligand scaffolds have received tremendous attention within the past two decades.^1^ These chain complexes are predominantly based on oligopyridyl amide ligands and have coined the term “extended metal atom chains” (EMACs)^2^ which are highly popular because of their potential in serving as conducting molecular wires and as single molecule magnets (SMMs).^3^ Nonconducting molecular strings of closed-shell transition metal centers are particularly interesting for Group 11 metal ions in the oxidation state +1 if they undergo d^10^···d^10^ metallophilic contact interactions.^4^ Such metallophilicity, which increases with increasing nuclear charge from Cu to Au due to increasing relativistic effects,^5−8^ is oftentimes associated with intense luminescence properties of coinage metal clusters.^9^ While mononuclear Ag^I^ and Au^I^ complexes without d^10^···d^10^ contacts already serve as efficient triplet emitters, the relatively small spin–orbit parameter ξ of copper (856.99 cm^–1^) in comparison to silver (1779.49 cm^–1^) and gold (5104.20 cm^–1^) usually impedes efficient phosphorescence of mononuclear Cu^I^ congeners (Scheme 1).^10^

However, it has been demonstrated by a series of mononuclear bis-phosphine/pyrrolide Group 11 metal complexes that Cu^I^ cations exhibit a significant d orbital contribution (through metal-to-ligand charge transfer, MLCT) to the lowest lying excited state. These Cu^I^ complexes have significantly larger intersystem crossing (ISC) rates and phosphorescence radiative decay rate constants (*k*_r_^P^) than their Ag^I^ and Au^I^ congeners.^11^ In addition, copper is significantly cheaper than its higher silver or gold homologs and has also become highly attractive as energy-saving molecular/organic light-emitting device (OLED) components due to the potent thermally activated delayed fluorescence (TADF) behavior of several Cu^I^ complexes.^12,13^ TADF is a powerful alternative to phosphorescence that is achieved by thermal population of the singlet S_1_ state from energetically close populating triplet excitons through reverse intersystem crossing (RISC, Scheme 1). Cu^I^ complexes show fast RISC rates that allow for quantitative depopulation of the S_1_ state and resulting monoexponential emission decays across a wide temperature range.

**Scheme 2:**
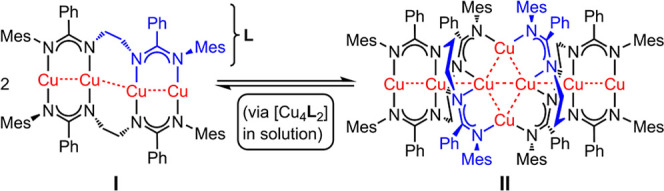
Formation of Complexes **I** and **II**

Despite these unquestionable advantages for applications in OLEDs,^9a−9c,9g−9i^ the number of linear multinuclear cuprous complexes with intramolecular Cu^I^···Cu^I^ contacts is still limited.^4^ This is because of the extreme weak nature of d^10^···d^10^ cuprophilic interactions.^14^ Except for rare examples of unsupported Cu^I^···Cu^I^ contacts,^15^ multinuclear Cu^I^ complexes with cuprophilic interactions usually require a tailored polydentate ligand framework to incorporate the Cu^I^ ions in close proximity (<2.8 Å, the sum of two Cu van der Waals radii)^16^ to each other. A series of amidinate and guanidinate bridging ligands^17^ has been found suitable for this purpose since corresponding binuclear Cu^I^ complexes originally served as examples for studies of closed-shell d^10^···d^10^ interactions.^18^ While binuclear cuprous complexes are well established, linear complex arrays with three or four Cu^I^ centers undergoing mutual d^10^···d^10^ contact interactions are still rare in comparison to related Ag^I^ and Au^I^ clusters.^4,9h^ There has been no example of such a linear pentanuclear Cu^I^ complex described yet. The longest discrete arrays of six Cu^I^ ions undergoing significant d^10^···d^10^ interactions has been reported by Chen and Tsai et al. as well as by our group (complex **II**, Scheme 2).^19,20^

We became interested in flexible connected bis(amidines) for linear Cu^I^ complexes because they are extremely versatile ligands,^21,22^ which have predominantly been employed in Groups 1–4,^23−26^ 13,^27^ and 14^28^ coordination chemistry, together with related catalytic studies. Less, although recently growing interest in late transition metal complexes of bis(amidines) and bis(amidinates) is reflected by examples of Groups 8–11^29−32^ that have also included multinuclear complex assemblies with more than two metal centers.^23c,31,32a^ We chose alkylene-linked bis(amidinates) not only for designing multinuclear clusters but also for creating defined linear arrangements of Cu^I^ ions that are, due to the flexibility of the linker, substantially bent and therefore adopt structures of helices. The combination of multiple Cu^I^ centers undergoing cuprophilic interactions overcomes their small individual spin–orbit-coupling contributions and can facilitate phosphorescence-based emission of these systems.^33^ Moreover, amidinates generally favor linear coordination spheres of the Cu^I^ centers and ensure rigid microenvironments in their interconnected binuclear {RC(NR′)_2_Cu_2_(NR′)_2_CR} compartments, which reduce large reorganization energies upon photoexcitation and also avoid Jahn–Teller distortions due to formal oxidation to d^9^ electron configurations originating from typically observed MLCT events in luminescent Cu^I^ complexes.^34^ Jahn–Teller distortions are known to increase nonradiative decay rates and further decrease intersystem crossing rates that are usually low in copper systems.

We have reported on a new ethylene-bridged bis(amidine) **L**H_2_ that combines sterical protection through bulky mesityl substituents with a flexible linker. **L**H_2_ cleanly reacts with mesitylcopper^35^ to afford the bis(amidinate) complex [Cu_2_**L**]_*n*_ in 74% yield which crystallized simultaneously into **I** and **II** (from toluene/hexanes mixtures) or exclusively as **II** (from toluene/diethyl ether, Scheme 2).^19^

X-ray crystallography revealed complex **I** as a rare twisted linear array of four Cu^I^ ions that are embedded in a bis(amidinate) scaffold. Upon dimerization, the unique octanuclear cluster assembly **II** is formed that possesses a straight linear arrangement of six Cu^I^ centers with two additional bridging cuprous ions constituting a central pseudorhombic Cu^I^_4_ core. Both **I** and **II** are potent blue- (**I**: *λ*_max_ = 460 nm, as solvate **I**·C_7_H_8_) and green-light emitters (**II**: *λ*_max_ = 495 nm).

**Scheme 3:**
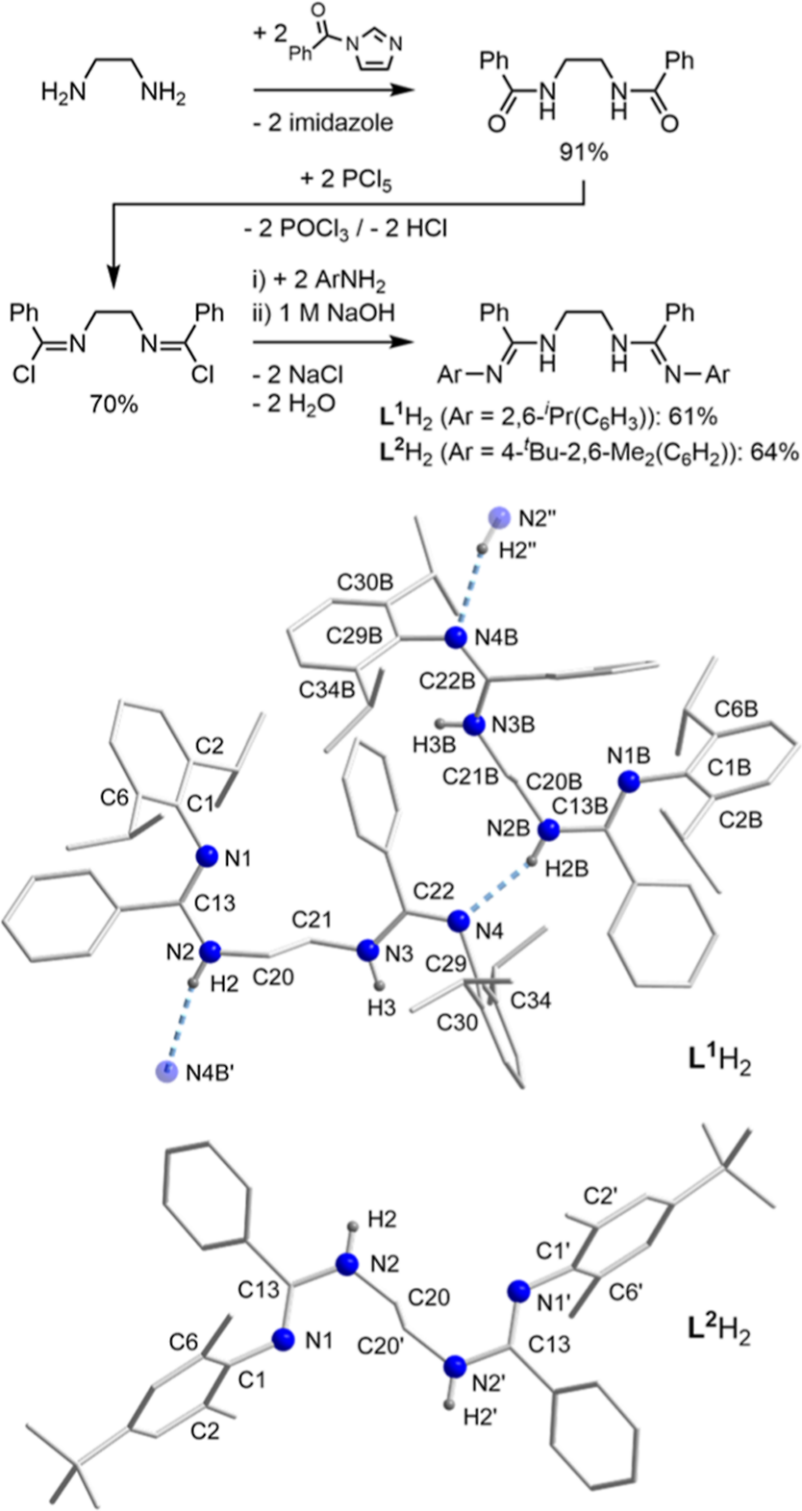
Synthesis of Ligands **L**^**1**^H_2_ and **L**^**2**^H_2_ and Their Single-Crystal XRD Molecular Structures Hydrogen atoms except for NH functionalities have been omitted for clarity. Symmetry operations used for **L**^**1**^H_2_ to generate equivalent atoms: (′) *x* – 1, *y*, *z* and (″) *x* + 1, *y*, *z*. Symmetry operation used for **L**^**2**^H_2_ to generate equivalent atoms: (′) −*x* + 5/3, *–y* + 4/3, −*z* + 1/3.

As subtle changes of the crystallization conditions (solvent polarity) have a fundamental impact on the selectivity of the formation of clusters **I** and **II**, we were curious about manipulations of the bis(amidinate) ligand backbone and its influence on the structures of [**L**Cu_2_]_*n*_ complexes. In this report, we focus on two new bis(amidines) **L**^**1**^H_2_ and **L**^**2**^H_2_ that offer enhanced sterical protection at the ortho position through bulky isopropyl substituents (**L**^**1**^H_2_) and increased solubility by ^*t*^Bu substituents in the 4-position of the terminal aryl groups (**L**^**2**^H_2_, Scheme 3). Upon clean conversion with mesitylcopper, **L**^**1**^H_2_ forms a straight linear Cu^I^_4_ cluster **1** in the crystalline state in which the tetracuprous chain is disconnected at the flexible diethylene bridge and thus represents a snapshot of the molecular dynamic process originally observed for **I**. By contrast, the corresponding bis(amidinate) complex **2** of **L**^**2**^H_2_ exhibits a tetranuclear Cu^I^ coil arrangement that resembles the structure of **I**, although **2** is significantly more twisted than **I**. Increasing the solvent polarity by using diethyl ether instead of toluene results in a rearrangement of **2** into the unprecedented Cu^I^_10_ cluster **3** through dimerization that is accompanied by a formal insertion of a [**L**^**2**^Cu_2_] fragment into the octanuclear Cu^I^ core assembly. Complexes **1–3** are efficient solid-state emitters in the visible spectral range, with quantum yields as high as 67% (**2**). Their photoluminescence (PL) properties show not only similarities but also remarkable differences which are primarily attributed to their varying core structures in the solid state. The PL has been studied in the temperature range of 5–320 K, as well as at ambient temperature under high pressure in a diamond anvil cell. TD-DFT calculations for the gas phase reveal a correlation between the void spaces of the crystal lattices of the tetranuclear complexes and the different emission behavior of these two clusters.

## Results and Discussion

### Synthesis, Structures, and Properties of **L**^**1**^H_2_ and **L**^**2**^H_2_

The synthesis of **L**^**1**^H_2_ and **L**^**2**^H_2_ was accomplished in three straightforward steps:^19,21^ benzoylation of ethylenediamine, chlorination of the resulting bis(amide), and aminolysis of the bis(imidoyl chloride). Both ligands were isolated as colorless microcrystalline solids in reasonable yields (up to 64%, Scheme 3). The ^1^H NMR spectra of **L**^**1**^H_2_ and **L**^**2**^H_2_ in C_6_D_6_ show broad resonance signals that suggest slow rotational motion of the bulky aromatic groups relative to the NMR time scale and proton exchange through tautomerization (Figures S36 and S40 in the Supporting Information). This is evidenced by a broad shoulder at δ = 3.27 ppm and an additional broad signal at δ = 7.81 ppm for **L**^**1**^H_2_ as well as additional broad signals at δ = 2.99 and 7.83 ppm for **L**^**2**^H_2_. These signals are indicative of *EE*/*EE*, *EZ*/*ZE*, and *ZZ*/*ZZ* C=N double bond as well as *syn*/*syn*, *syn*/*anti*, and *anti*/*anti* stereoisomers in solution that interconvert through tautomerization and single-bond rotation at the amidine moieties.^36^ Since more polar solvents suppress the underlying prototopic exchange for this isomerization, additional signals in the DMSO-*d*_6_^1^H NMR spectra of both bis(amidines) disappear and also show decreased line broadening, although to a lesser extent for **L**^**1**^H_2_ than for **L**^**2**^H_2_. Consequently, also the ^13^C NMR spectra of **L**^**1**^H_2_ and **L**^**2**^H_2_ in both solvents show broadened signals (Figures S37, S39, S41, and S43).

To examine the molecular structures in the solid state, single crystals of **L**^**1**^H_2_ and **L**^**2**^H_2_ suitable for X-ray structure analyses were both grown from diethyl ether solutions (Scheme 3). **L**^**1**^H_2_ was found to crystallize in the triclinic space group *P*1̅ with two independent molecules in the asymmetric unit and four molecules in the unit cell (Figure S3 and Table S1). By contrast, ligand **L**^**2**^H_2_ crystallized in the trigonal space group *R*3̅ with half of the molecule occupying the asymmetric unit and nine molecules being present in the unit cell (Figure S6 and Table S1). Increased steric bulk in the *ortho* positions of **L**^**1**^H_2_ in comparison to **L**H_2_,^19^ which occurs as an *EE*(*syn*/*syn*) isomer in the solid state, results in a preference for *Z*(*anti*) at one-half of the molecule of **L**^**1**^H_2_ whereas *E*(*syn*) is retained at the other half. The *Z*(*anti*) compartment of **L**^**1**^H_2_ features a considerably larger N=C–NH angle (125.58(17)°) than in *E*(*syn*) (**L**^**1**^H_2_: 119.47(17)°), which is consistent with the increased steric constraint for the latter. For this reason, the originally encapsulated bis(amidine) moiety of **L** unfolds in **L**^**1**^H_2_ and exposes the NH protons to hydrogen bonding. In consequence, an alternating polymeric chain of monomeric *EZ*(*syn*/*anti*) isomers of **L**^**1**^H_2_ that are linked through weak to moderately strong^37^ intermolecular NH···N′ hydrogen bonds is observed (Scheme 3 and Figure S1). Very similar to the sterically crowded bis(amidine) **L**H_2_,^19,21^ ligand **L**^**2**^H_2_ also features the *EE*(*syn*/*syn*) isomeric configuration in the crystalline state (Scheme 3 and Figure S4). This results in considerable shielding of the bis(amidine) *N*-donor sites and prevents the NH groups from forming hydrogen bonds.

Consistently, the IR *ν*(N–H) stretching frequency of **L**^**2**^H_2_ is blue-shifted by 36–98 cm^–1^ relative to **L**^**1**^H_2_. As previously observed for a series of ethylene-linked bis(amidines),^21,22,23a,23c^ the NH protons of **L**^**1**^H_2_ and **L**^**2**^H_2_ are localized at the central −NH(CH_2_)_2_NH– diamine bridge. Its resulting N–C single bond character is reflected by large Δ_CN_ values^38^ for both bis(amidines) (**L**^**1**^H_2_, *E*: 0.076 Å, *Z*: 0.060 Å; **L**^**2**^H_2_: 0.083 Å) that confirm only a low extent of delocalization within the −N=C–N– moieties.

### Synthesis and Properties of [**L**^**1**^Cu_2_] and [**L**^**2**^Cu_2_]. Formation and Solid-State Structures of 1·1.5C_7_H_8_, 2·C_7_H_8_, and 3·5Et_2_O

**Scheme 4:**
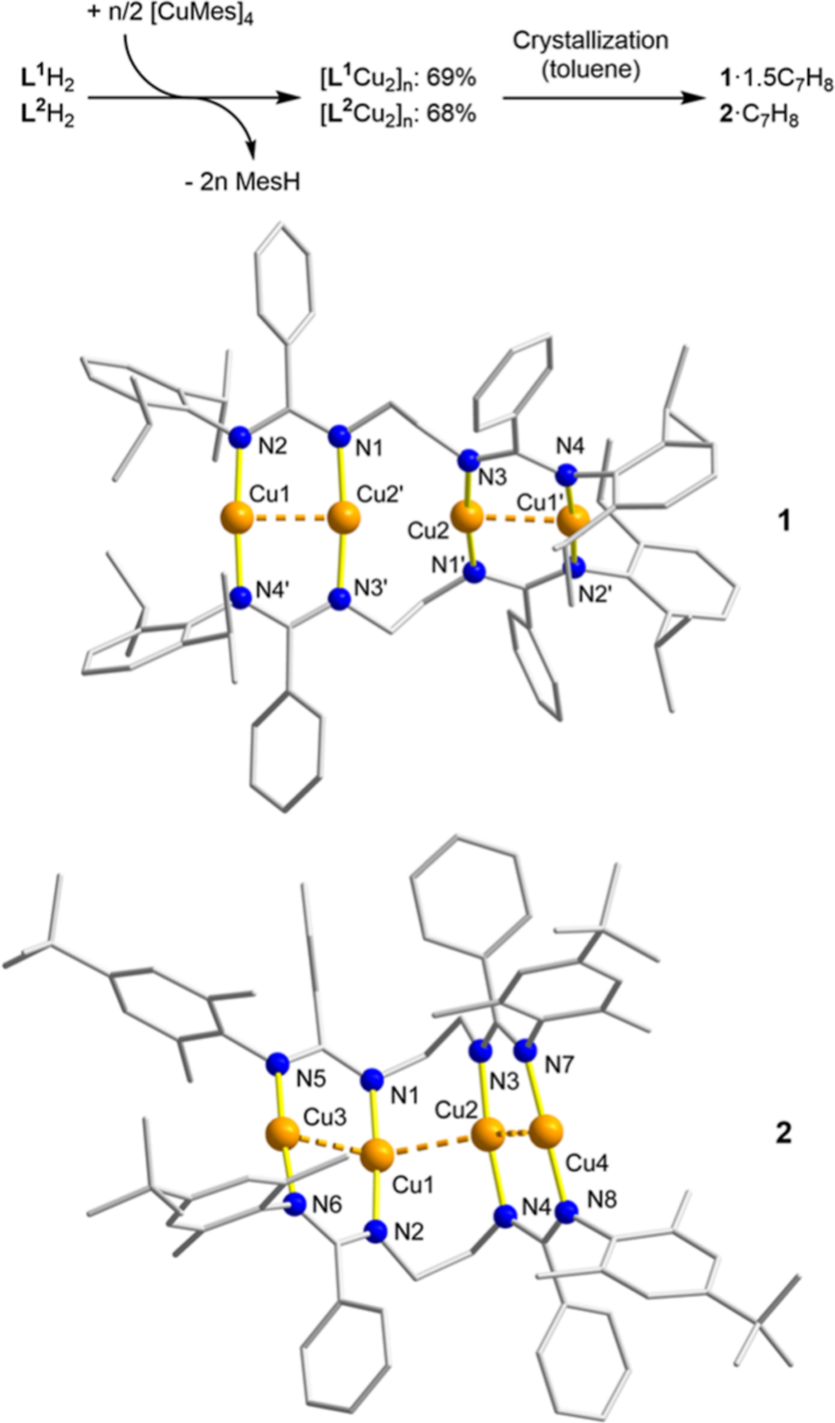
Synthesis of Complexes [**L**^**1**^Cu_2_] and [**L**^**2**^Cu_2_] Crystallization of **1**·1.5C_7_H_8_ and **2**·C_7_H_8_. Single-crystal XRD molecular structures of **1** and **2** (*P* (Δ) enantiomers). Toluene molecules and hydrogen atoms have been omitted for clarity. Symmetry operation used for **1** to generate equivalent atoms: *–*x** + 1, −**y** + 1, −*z* + 1 and (′) *x*, *–y* + 1, *z* + 1/2.

Ligands **L**^**1**^H_2_ and **L**^**2**^H_2_ undergo a clean conversion with two equivalents of mesitylcopper in toluene at −35 °C and subsequent warming to room temperature to form the corresponding homoleptic Cu^I^ complexes of the stoichiometric composition ligand/Cu of 1:2 as almost colorless to pale yellow microcrystalline solids in good yields ([**L**^**1**^Cu_2_]_*n*_: 69% and [**L**^**2**^Cu_2_]_*n*_: 68%, Scheme 4).

Both Cu^I^ bis(amidinates) [**L**^**1**^Cu_2_]_*n*_ and [**L**^**2**^Cu_2_]_*n*_ are reasonably air-stable in the solid state and decompose at temperatures above 240 °C into a brown oil. Decomposition in solution (C_6_D_6_) was observed over the course of a few days at room temperature, even in an argon atmosphere.

The IR spectra of [**L**^**1**^Cu_2_]_*n*_ and [**L**^**2**^Cu_2_]_*n*_ indicate complex formation by the absence of ν(N–H) stretching frequencies. Both ^1^H NMR and ^13^C NMR spectra in C_6_D_6_ show one set of resonance signals of the ligand frameworks originating from (**L**^**1**^)^2–^ and (**L**^**2**^)^2–^, respectively, and therefore indicate the presence of one symmetrical complex species in solution (Figures S44–S47). All aliphatic and aromatic signals of the ^1^H NMR spectra appear as narrow singlets or show well-resolved splitting patterns. Most characteristic for metal ion coordination are significant ^13^C NMR downfield-shifts of the CH_2_ signal by 14.8 ppm ([**L**^**1**^Cu_2_]_*n*_) and 12.1 ppm ([**L**^**2**^Cu_2_]_*n*_) as well as of the quaternary CN_2_ amidinate resonance signals by 21.8 ppm ([**L**^**1**^Cu_2_]_*n*_) and 20.0 ppm ([**L**^**2**^Cu_2_]_*n*_). Although the molecular ion peak is not observed, ESI(+) mass spectrometry evidenced common characteristic fragments [(**L**^**1,2**^H_2_)(**L**^**1,2**^H)Cu_2_]^+^ and [(**L**^**1,2**^H)_2_Cu]^+^ at *m*/*z* = 1297.7 and 1235.7, respectively.

Crystallization of [**L**^**1**^Cu_2_]_*n*_ and [**L**^**2**^Cu_2_]_*n*_ afforded the formation of complexes **1**·1.5C_7_H_8_ and **2**·C_7_H_8_ (Scheme 4). Single crystals suitable for XRD analyses were obtained from slowly evaporating (**1**·1.5C_7_H_8_) or saturated toluene solutions (**2**·C_7_H_8_) at room temperature or −35 °C, respectively. Under exposure to UV light, both crystalline materials exhibit bright blue emissions with maxima at ≈470 nm. Complex **1** crystallized in the monoclinic space group *C*2/*c* and half of one molecule of **1** was found in the asymmetric unit to translate into four molecules in the unit cell, together with overall six cocrystallizing toluene molecules (Figure S9 and Table S2). Complex **2** formed a solvate **2**·C_7_H_8_ in the monoclinic space group *P*2_1_/*n* with one molecule of **2** and toluene each being present in the asymmetric unit (four molecules were found in the unit cell, Figure S13 and Table S2).

Complex **1** consists of two sets of double-*μ*-1,3-amidinate-bridged Cu^I^_2_ segments that are terminally interconnected by two flexible ethylene linkers (Scheme 4 and Figure S7). Similar to **I**,^19^ the structural motif of these eight-membered dicopper(I)-diamidinate units resembles the one of well-established binuclear Cu^I^ diamidinate complexes.^18^

The peripheral Cu^I^ centers adopt distorted linear or T-shaped coordination geometries, the latter if d^10^···d^10^ contacts are considered as chemical bonds, as indicated by the N4′–Cu1–N2/N4–Cu1′–N2′ angles of 175.67(9)°. This deviation from linearity is more distinct for the inner cuprous ions (N3′–Cu2′–N1: 173.69(9)°). The terminal (CN_2_)Cu_2_ rings of **1** exhibit a substantial twist (N4′–Cu1–Cu2′–N1: −171.4(1)°) which is typical of dicopper-diamidinate complexes^18^ and was previously observed for complex **I** [N1–Cu1–Cu2′–N3′: 168.169(2)°].^19^ In contrast to the helically bent chain of four Cu^I^ ions in **I** [Cu_(out)_–Cu_(in)_–Cu_(in)_′: 139.79(3)°], complex **1** shows an almost ideal linear array of four Cu^I^ ions as reflected by an Cu_(out)_–Cu_(in)_–Cu_(in)_′ angle of 176.400(15)°, although the helical bending of the dibis(amidinate) ligand framework is even more pronounced in **1** than in **I** (Figure S19). This is obvious by the torsion angles of the *N*-donor atoms defining the central 10-membered −{*μ*-(Cu_(in)_)(N–CH_2_–CH_2_–N)_2_(Cu_(in)_)}– rings in both complexes (*P* (Δ) enantiomers, **1**: N3′–N1–N3–N1′, 40.31(6)°; **I**: N3–N2′–N3′–N2, −28.852(1)°).^19,39^ In contrast to **I**, the Cu^I^_4_ chain in **1** undergoes a formal relaxation that is accompanied by a disconnection of the central Cu_(in)_···Cu_(in)_′ contact, as evidenced by an intermetallic distance of 2.8702(6) Å, which is clearly longer than the sum of two copper van der Waals radii of 2.8 Å.^16^ The separated dicopper(I) units in **1** reveal slightly longer Cu^I^···Cu^I^ distances (2.4771(4) Å) than in **I** and **2** (2.4398(9)–2.4640(13) Å). Overall, the centrally disconnected (Cu_(out)_···Cu_(in)_)(Cu_(in)_′···Cu_(out)_′) chain in **1** represents a remarkable snapshot of the molecular dynamic process previously observed in solution for **I**.

The molecular structure of **2** is very similar to **I** and consists of two ethylene-interlinked sets of double-μ-1,3-amidinate-bridged dicopper(I) segments with short Cu^I^···Cu^I^ distances (2.4398(9) Å and 2.4579(9) Å) that are indicative of significant d^10^···d^10^ contacts (Scheme 4 and Figure S11). The two central Cu^I^ ions undergo cuprophilic interactions as well, although to a lower extent (Cu^I^···Cu^I^: 2.6464(9) Å), but they are more significant if this distance is compared to the inner Cu^I^···Cu^I^ separation of **I** (2.6796(17) Å). In consequence, a continuous array of four cuprous ions with intermetallic contact distances is observed. If these contacts are considered as chemical bonds, then the central Cu^I^ centers adopt distorted-seesaw coordination geometries, with smaller equatorial Cu_(out)_–Cu_(in)_–Cu_(in)_′ angles (131.37(3)° and 137.41(4)°) than in **I** (139.79(3)°), thus indicating a more twisted Cu^I^_4_ chain in **2** (Figure S19).

**Scheme 5:**
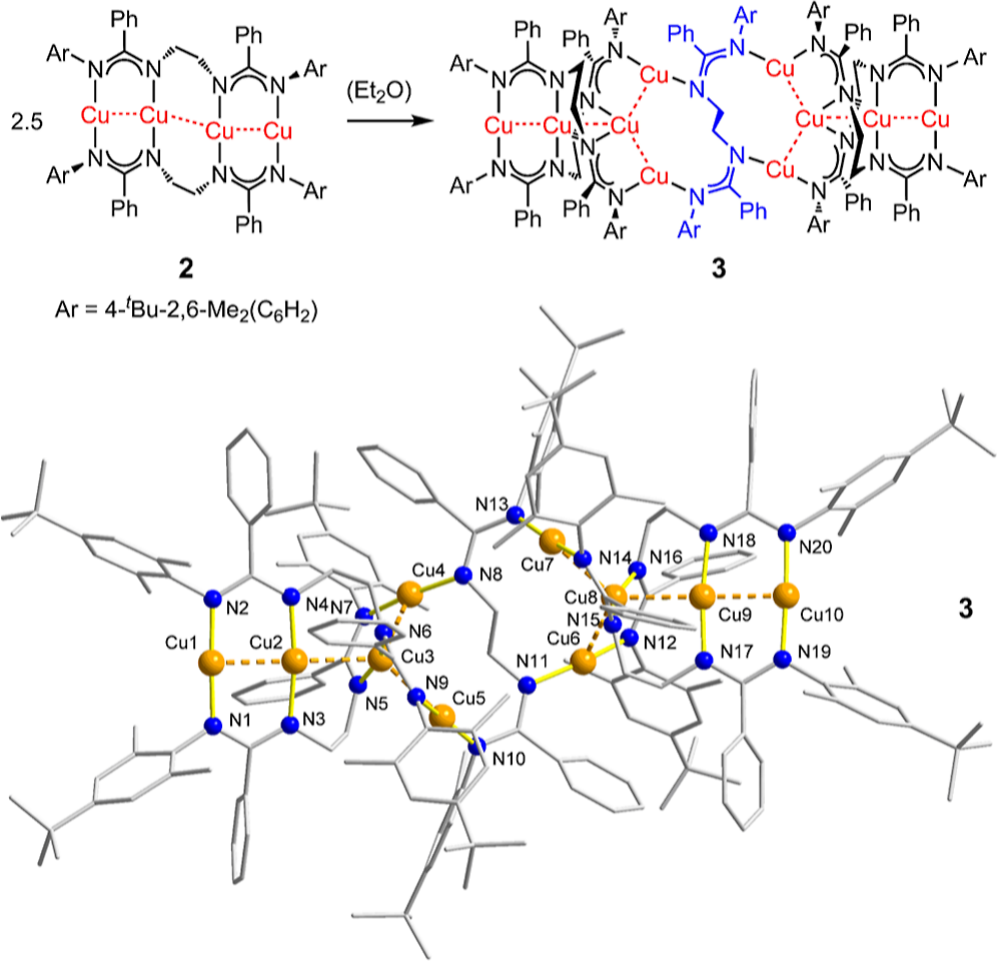
Formation of Complex **3**·5Et_2_O upon Crystallization from Diethyl Ether Single-crystal XRD molecular structure of **3** (*P* (Δ) enantiomer). Diethyl ether molecules and hydrogen atoms have been omitted for clarity. Symmetry operation used to generate equivalent atoms: *–x* + 1, *–y* + 1, *–z*.

We noticed that [**L**^**2**^Cu_2_]_*n*_ is substantially more soluble in common organic solvents (THF, toluene, C_6_D_6_) than [**L**^**1**^Cu_2_]_*n*_. It is even possible to obtain clear solutions of [**L**^**2**^Cu_2_]_*n*_ in diethyl ether. Upon slow evaporation at room temperature, pale yellow, almost colorless single crystals of a new complex **3** were obtained that emit green-yellowish light (*λ*_max(295K)_ ≈ 525 nm) under UV light exposure (Scheme 5). An X-ray crystallographic structure determination shows that **3** crystallized in space group *P*1̅ with two independent molecular units in the asymmetric unit and four molecules in the unit cell, together with overall 20 diethyl ether solvent molecules (Figure S17). The structure of **3**·5Et_2_O is represented by a nanoscaled molecular aggregate with an approximate length of 29 Å and a height of 20 Å consisting of two Y-shaped pentanuclear Cu^I^ clusters [**L**_2_^**2**^Cu_5_]^+^ that are interconnected by a flexible bis(amidinate) bridge (**L**^**2**^)^2–^ (Scheme 5, Figure S15, Table S2).

A density functional theory (DFT, PBE0) gas-phase investigation revealed an overall exothermic (Δ*H* = −15.46 kcal·mol^–1^) but endergonic (Δ*G* = 13.94 kcal·mol^–1^) aggregation process for 2.5 × **2** → **3**. This is consistent with our previous observation for the dimerization of **I** to **II**, in which also a negative formation enthalpy (Δ*H* = −4.76 kcal·mol^–1^) and positive free energy change (Δ*G* = 17.88 kcal·mol^–1^) was found for the gas phase.^19^ The aggregation energy is expected to be small for both transformations because the two cluster pairs (**2** and **3** as well as **I** and **II**, respectively) crystallize at the same temperature conditions and can even coexist through simultaneous formation (**I** and **II**). The PBE0 calculations were performed in the gas phase and therefore overestimate the entropy contribution to the free energy for the solid state. Generally, DFT underestimates the stabilization of the larger clusters **3** and **II** by additional van der Waals interactions which have been found as significant contributions in related binuclear bis(amidine) coinage complexes.^32^ Taking these factors into account, the calculated free energy of aggregation for **3** is within reason for its formation in the crystalline state.

The decanuclear Cu^I^ cluster **3** is likely formed from an intermediate dimeric Cu^I^_8_ assembly similar to **II** (Scheme 2) that subsequently incorporates a [**L**^**2**^Cu_2_] fragment by formal insertion into the original central pseudorhombic Cu^I^_4_ core. Two molecular units of [**L**_2_^**2**^Cu_4_] open one terminal (CN_2_)Cu_2_ ring to aggregate with one additional Cu^I^ ion each that is formally inserted into one Cu–N bond of each of the two diamidinate-dicuprous segments. This rearrangement of two formerly twisted [**L**_2_^**2**^Cu_4_] parent complexes relieves strain on the helically bent Cu^I^_4_ chain to yield a straight linear arrangement of three Cu^I^ ions within the two Y-shaped Cu^I^_5_ cluster compartments (Cu1–Cu2–Cu3: 176.64(3)°; Cu10–Cu9–Cu8: 177.37(3)°). Similar to complex **II**, the helical twist of the bis(amidinate) ligands is thereby enforced, as reflected by the significantly larger torsion angles within the double-bridge {−N–CH_2_–CH_2_–N−}_2_ moieties of **3** in comparison to **2** (**3**: N4–N3–N5–N6: 45.60(9)°;^39^ N15–N16–N18–N17: 45.07(9)°;^39^ and **2**: N1–N2–N4–N3: 27.2(1)°).^19^ To ensure balancing the charge and coordinative saturation of the two cuprous ions, an additional (**L**^**2**^)^2–^ bis(amidinate) acts as a bridging ligand for a total of four Cu^I^ centers. While the terminal cuprous centers of this central [**L**^**2**^Cu_4_]^2+^ unit are coordinated in the usual *E* fashion known from several dicopper-diamidinate complexes,^18^ the inner Cu^I^ centers adopt a unique side-on coordination originating from a *syn*/*syn* conformational orientation of the bis(amidinate) ligand as it is observed in the solid-state structure of **L**^**2**^H_2_ (*vide supra*). All five cuprous ions in each compartment undergo d^10^···d^10^ contact interactions, as indicated by intermetallic distances ranging between 2.4783(7) and 2.6664(8) Å. If these interactions are considered as chemical bonds, then the individual Cu^I^ centers exhibit T-shaped, square-planar, or trigonal-bipyramidal^40^ coordination geometries, respectively. Similar to the Cu^I^_8_ cluster **II**, the peripheral tethered dicopper-diamidinate units show elongated Cu^I^···Cu^I^ separations (Cu2···Cu3: 2.6077(7) Å; Cu9···Cu8: 2.6437(7) Å) to the neighbored Cu3/Cu8 centers in comparison with the intrasegmental Cu1(Cu10)···Cu2(Cu9) distances (2.4783(7) Å and 2.4874(7) Å, respectively). However, the corresponding distances to the central Cu^I^_4_ rhomb in **II** are significantly longer by about 0.06–0.09 Å.

All three complexes **1**–**3** crystallized as racemic mixtures of *P* (Δ) and *M* (Λ) isomers (Figures S9, S13, and S17). Since the arrays of Cu^I^ ions in **1** and **3** are strictly linear, the helicity of these complexes is exclusively determined by the bis(amidinate) ligand framework. This is in striking contrast to complex **2** in which the tetranuclear Cu^I^_4_ chain is also helically bent.

### DFT Calculations Related to the Structures of **1–3**

The ground-state optimized structures of **1** and **2** for the gas phase undergo notable geometrical changes relative to the X-ray structures that predominantly originate from the common flexible −{*μ*-(Cu_(in)_)(N–CH_2_–CH_2_–N)_2_(Cu_(in)_)}– rings (Figures S20 and S21). This is most obviously indicated by a bending tetracuprous chain in **1** upon computational geometry optimization, resulting in a deviation of 17.0–17.1° from approximate linearity of the crystal structure analysis (Table S3). This structural change is accompanied by a significant decrease of the central Cu2···Cu2′ distance by 0.225 Å, thus establishing a contact interaction of the originally disconnected (Cu^I^_(out)_ ··Cu^I^_(in)_)(Cu^I^_(in)_′···Cu^I^_(out)_′) chain. The geometry-optimized computational structure of **2** shows a tendency of decreased bending of the Cu^I^_4_ chain, as reflected by an increase of the Cu3–Cu1–Cu2 and Cu4–Cu2–Cu1 angles of 4.6 and 10.6°, respectively (Table S4). The existing central Cu1···Cu2 cuprophilic interaction remains intact (2.664 Å). This trend is consistent with previous DFT calculations on **I**, which suggests that both complexes **I** and **2** undergo crystal packing effects that support the helical bend of the common Cu^I^_4_ chain.^19^ Overall, the structural changes of the Cu^I^_4_ chain through computational geometry optimization are more pronounced in complex **1** than in **2**. In consequence, both complex molecules become more similar in terms of the central Cu^I^···Cu^I^ contact and helical bending in the gas phase when compared to the crystalline state. In contrast, the more rigid ligand scaffold of **3** allows for a reasonable agreement between the crystal structure analysis and the gas-phase geometry-optimized structure (Figure S22).

**Figure 1:**
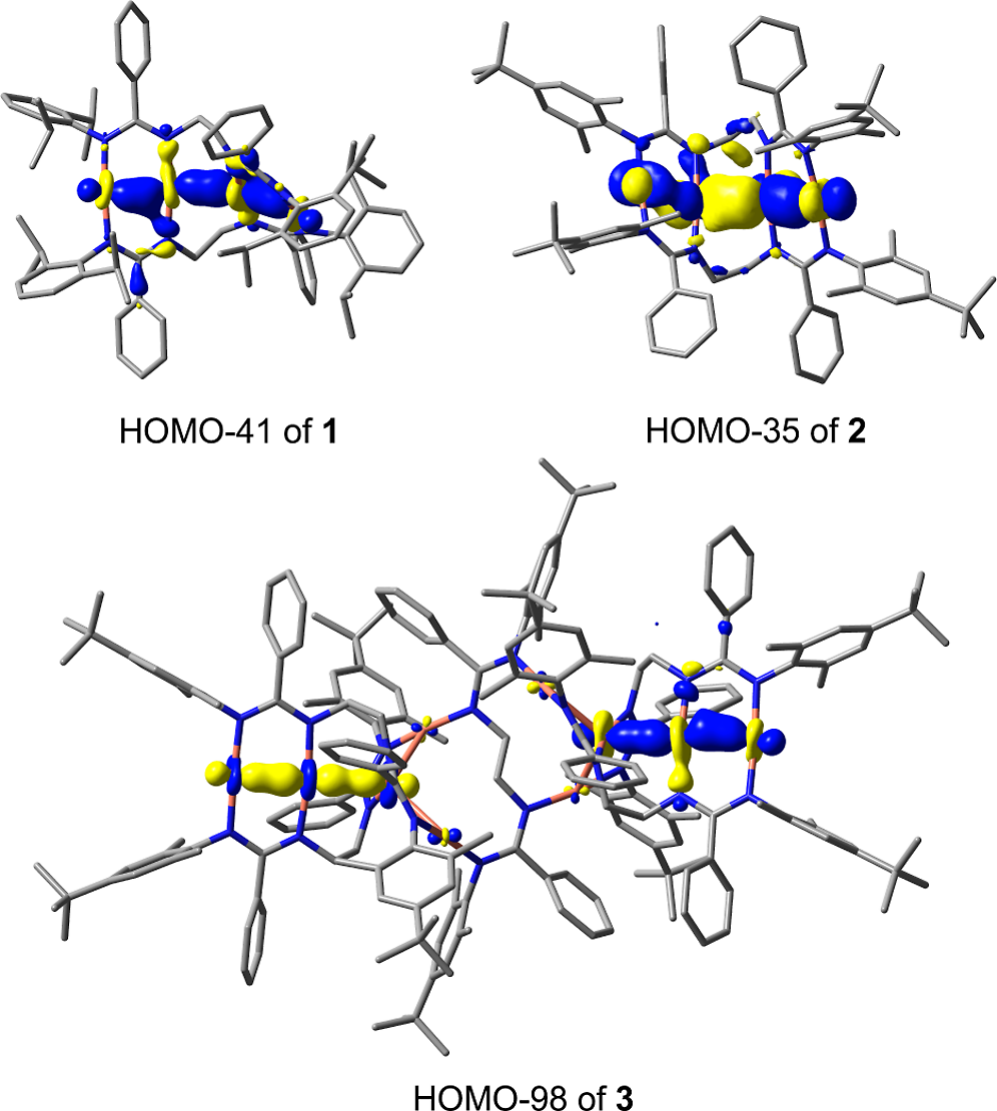
Isodensity plots of selected molecular orbitals of **1**–**3** showing electron delocalization across the Cu centers (isovalues = 0.04 au for **1** and **3**, and 0.02 au for **2**).

The connected chain character of the computational structure of **1** is confirmed by its orbital analysis (Figures 1 and S23–S25). Selected molecular orbitals clearly show delocalization of the *σ* bonding orbitals of the Cu atoms (MO 333 of **1**) as well as *π* bonding within the Cu^I^_4_ chain (MO 339 of **2**, see also Figures S26–S28). For the decanuclear cluster **3**, electron density is mainly observed in the linear tricuprous segments (Figures 1 and S29–S31). While this delocalization does not necessarily mean that there is a net bonding character between the Cu atoms, these occupied orbitals, in addition to Cu^I^···Cu^I^ contacts shorter than the sum of two copper van der Waals radii, indicate the existence of weak cuprophilic interactions.

### Photoluminescence Properties of **1–3** and Their Solvates

**Figure 2:**
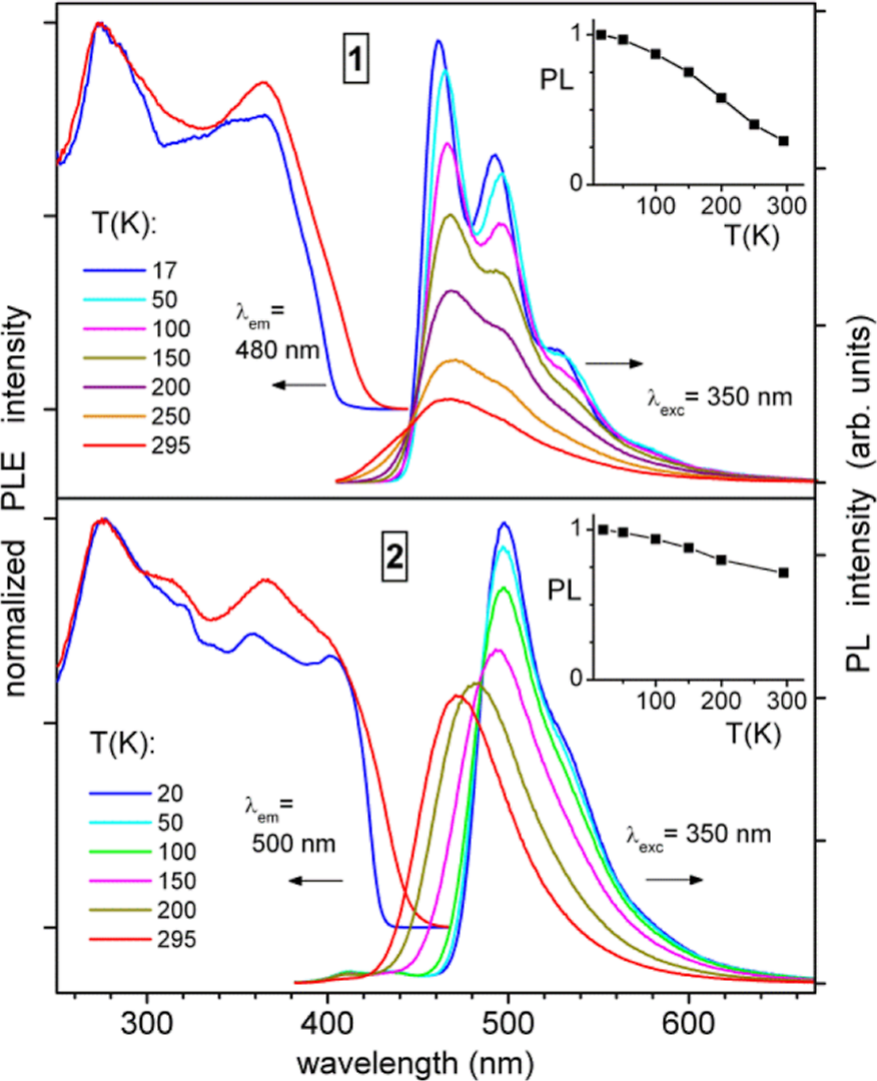
Temperature-dependent photoluminescence emission (PL) and excitation (PLE) spectra of polycrystalline complexes **1**·1.5C_7_H_8_ and **2**·C_7_H_8_. The inserts show an integral PL intensity (normalized to unity) as a function of temperature.

Figure 2 compares PL emission and excitation (PLE) spectra of the tetranuclear complexes **1**·1.5C_7_H_8_ and **2**·C_7_H_8_ in the temperature range of 17–295 K. The PLE onset at ≈430–450 nm at ambient temperature corresponds well to the pale yellow color of the crystalline materials. Blue/blue-green emission peaks were found at 467/461 and 472/495 nm for **1**·1.5C_7_H_8_ and **2**·C_7_H_8_, respectively, when measured at 295/20 K. Complex **1**·1.5C_7_H_8_ features a quantum yield of 6.5% at 295 K (**1**·C_7_H_8_: 17%) and was determined using an integrating sphere (excitation at 400 nm). A distinct feature of the low-temperature emission of **1**·1.5C_7_H_8_ is a pronounced vibronic progression with a characteristic frequency of ∼1400 cm^–1^, likely relating to a vibration of the amidinate bridge. A similar feature is also observed for **2**·C_7_H_8_, but only as a shoulder on the emission curve. The PL of **1**·1.5C_7_H_8_ and of **2**·C_7_H_8_ at low temperatures is phosphorescence as indicated by a long (tens of μs) emission decay under pulsed laser excitation (Figures S48 and S49). The PL decay of **1**·1.5C_7_H_8_ only moderately accelerates at 295 versus 18 K, roughly correlating with the decrease in PL intensity. In contrast, **2**·C_7_H_8_ shows a significant variation of the decay time, τ, following a temperature dependence characteristic for thermally activated delayed fluorescence (TADF), i.e., emission from the singlet S_1_ state thermally populated from the low-lying, energetically close triplet T_1_ state. Accordingly, the T_1_ phosphorescence of **2**·C_7_H_8_ observed below ∼120 K (average τ of 53 *μ*s) transforms to TADF by raising the temperature. The latter is dominating above 200 K, resulting in the effective PL lifetime of 1.4 *μ*s at 325 K. By applying the simple TADF model of the thermally equilibrated S_1_ and T_1_ states,^13a−13c^ the energy separation between these states, Δ*E*(S_1_–T_1_), and intrinsic S_1_ lifetime can be estimated as 840 cm^–1^ and 16 ns (Figure S49). The TADF mechanism also agrees with the emission blueshift (∼1000 cm^–1^) between 295 and <100 K.

Remarkably, after washing with hexanes and vacuum drying, the PL efficiencies of **1** and **2** at 295 K soar to 58 and 67% (*λ*_exc_ = 400 nm), respectively. This can be attributed to removal of cocrystallized solvent molecules (C_7_H_8_) which might contribute to nonradiative electronic relaxation. In comparison to **2**·C_7_H_8_, the emission of **2** shows a moderate redshift (*λ*_max(295K)_ = 495 nm, Figure S50). Due to such a high quantum yield, the perceived color of **2** in daylight turns to green, similar to “neon colors” known for highly efficient fluorophors.^41^ The parameters of the TADF-characteristic emission decay, in particular the estimate of Δ*E*(S_1_–T_1_), remain, however, standing (Figure S51).

**Figure 3:**
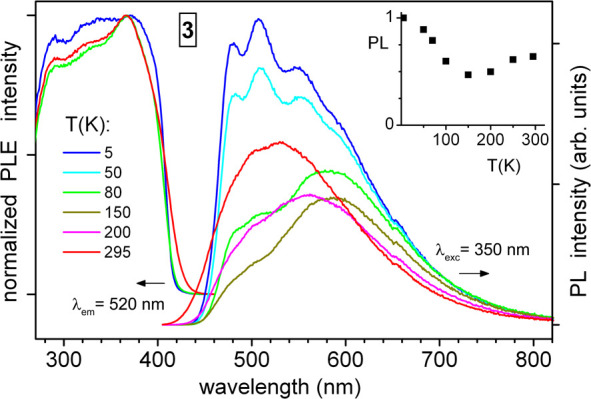
Temperature-dependent photoluminescence emission (PL) and excitation (PLE) spectra of polycrystalline complex **3**·5Et_2_O, excited. The inset shows an integral PL intensity (normalized to unity) as a function of temperature.

The decanuclear cluster complex **3**·5Et_2_O demonstrates similar solid-state PLE spectra as **1** and **2** (with the onset at ≈430 nm at 295 K), but green-yellowish phosphorescence which is spectrally very broad, tailing up to about 750 nm (Figure 3). Its quantum efficiency was determined as 12.5% at ambient temperature (*λ*_exc_ = 400 nm). The emission of **3**·5Et_2_O follows a rather unusual thermochromism pattern: its maximum red-shifts to ≈590 nm (and the emission color changes to orange) by cooling down to about 150 K, and blue-shifts back to 506 nm (the maximum of a moderately pronounced vibronic pattern similar to that in **1**) by further cooling below 50 K.

This change is accompanied by a specific “U-shaped” variation of the integral PL intensity: this first decreases by cooling down to 150 K, and then again increases at lower temperatures (Figure 3). The mechanistic details behind such behavior are presently not clear. Tentatively, we attribute this to complicated multichannel electronic relaxation in **3**·5Et_2_O, likely related to the large, double-unit core structure. Such picture appears to be supported by complicated PL kinetics observed for this cluster compound below ∼200 K. Especially in the intermediate temperature range of 100–200 K, the PL decay is clearly nonexponential and dependent on the emission wavelength (Figure S52).

Additional experimental data on the relation between a structure and photophysical properties were delivered by high pressure measurements in a diamond anvil cell up to 30 kbar (the maximum level probed in this work). Within this pressure range, **1**, **2** (chosen as the most bright emitter), and **3**·5Et_2_O display significant redshifts of both excitation and emission spectra, accompanied by acceleration of the PL decay (Figures S53–S55). Such shifts can be expected for the excited states of MLCT character, extending over a molecular framework (see DFT and TD-DFT Calculations Related to the Photophysical Properties of **1–3** below). Specifically, the PLE onsets approximately linearly shift up to 50–70 nm under 25–30 kbar pressure, corresponding to energy shifts of −(10–15) meV/kbar. The effect on the phosphorescence corresponds to a shift of about −10 meV/kbar for **1** and **2**, and −4 meV/kbar for **3**·5Et_2_O. Somewhat unexpectedly, we found for complexes **1**, **2**, and **3**·5Et_2_O a high resilience to (quasi)hydrostatic pressure (up to 30 kbar). After pressure release, the PL spectra, emission intensity, and decay almost recover for **1**, and practically completely recover for **2** and **3**·5Et_2_O. The higher pressure resilience of the latter most likely correlate with the less void space in their crystal structures (see above). The decanuclear cluster appears thereby to be the most pressure-resilient structure. Regardless of the spectral shifts, application of high pressure only moderately reduces its emission intensity (Figure S55). An interesting pressure effect is also observed regarding the TADF emission of **2**. Its redshift notably increases and intensity decreases above ∼15 kbar (Figure S54). Even more remarkable are the fast PL decay and the appearance of a prompt fluorescence component in decay traces above ∼15 kbar. These observations suggest that TADF is not sustained in **2** under high pressure (but recovers after pressure release).

### DFT and TD-DFT Calculations Related to the Photophysical Properties of **1–3**

**Figure 4:**
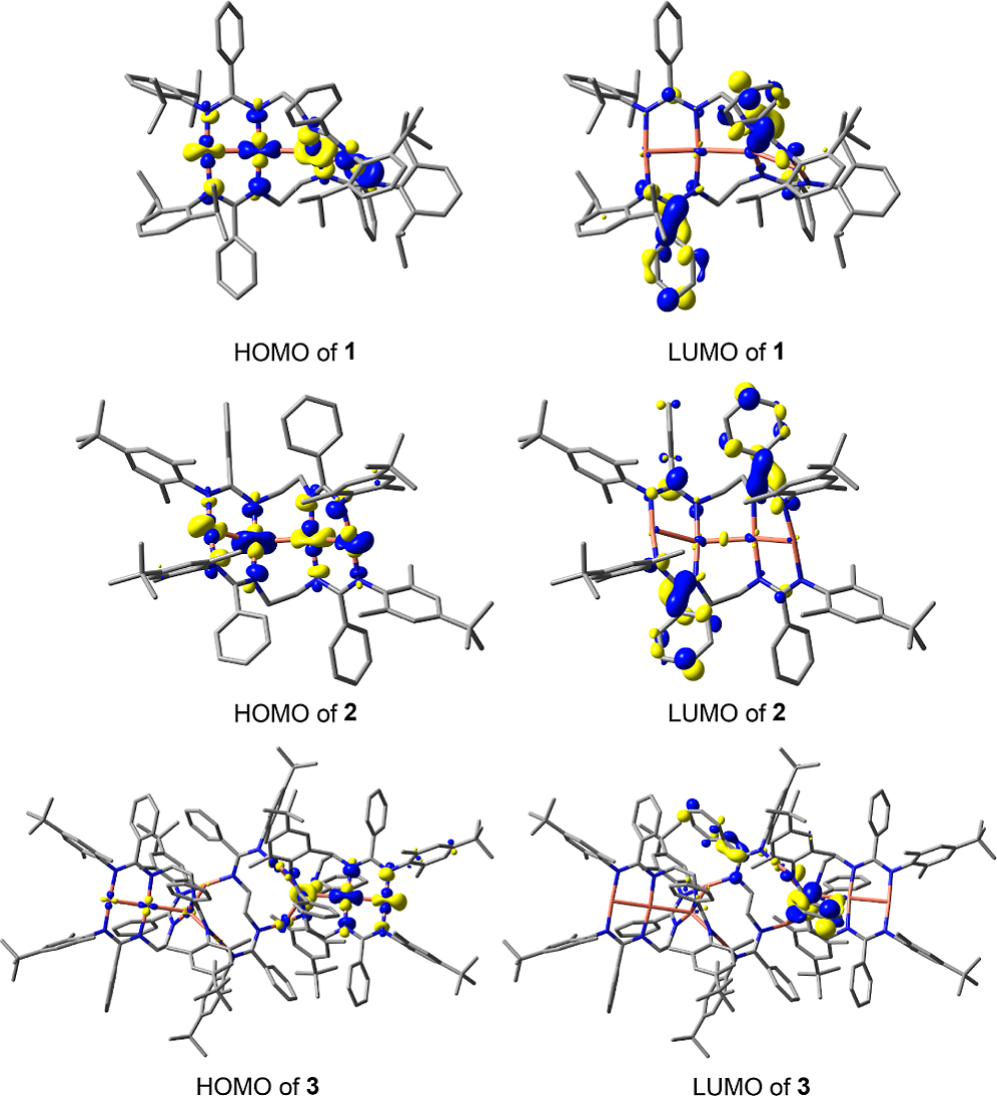
Isodensity plots of the highest occupied molecular orbitals (HOMOs) and lowest unoccupied molecular orbitals (LUMOs) of **1**–**3** (iso value = 0.04 au).

Inspection of the frontier orbitals of **1**–**3** reveals the charge-transfer character of the lowest excited states of all three complexes, with major contributions from MLCT (Figure 4). The HOMOs of all three complexes are largely derived from the Cu^I^ 3d atomic orbitals but also contain contributions from the amidinate *N*-donor atoms. Very little electron density is observed on the terminal aromatic substituents of **1**–**3**. The LUMOs are predominantly located on the phenyl groups and the sp^2^-hybridized amidinate carbon atoms. TD-DFT and natural transition orbital (NTO) calculations (Figure S32) indicate that the experimentally observed S_1_ ↔ S_0_ and T_1_ ↔ S_0_ transitions correlate with leading electron/hole NTOs that describe the excitation character with a weight of over 84% for **1** and **2**, and 20.3–97.5% for **3**. NTOs more compactly represent the character of the excitation than the canonical molecular orbitals depicted in Figure 4. The NTO pairs of both complexes **1** and **2** clearly show electron density at the central Cu^I^_(in)_···Cu^I^_(in)_′ contact (Figure S32) that is apparently induced upon closure of the Cu^I^_4_ chain in the case of complex **1**. This observation is further supported by a significant decrease of the central Cu^I^_(in)_···Cu^I^_(in)_′ contact distances in **1** and **2** upon excitation to the S_1_ and T_1_ states. By contrast, complex **3** exhibits substantial ligand-to-ligand charge transfer (LLCT) for T_1_. On the basis of these computational results, the lowest singlet and triplet excited states of **1**–**3** are assigned as S_1_ = ^1^MLCT (dπ*) T_1_ = ^3^MLCT, (dπ*) states. The T_1_ state of **3** is dominated by LLCT (pπ*) transitions.

The calculated S_1_–T_1_ adiabatic energy separations of **1** and **2** are very similar for the gas phase (**1**: 2071 cm^–1^ and **2**: 2180 cm^–1^), although each of them has a different ground-state geometry (Figure S33). Nevertheless, these geometry-optimized structures are more similar to each other than in the solid state (*vide infra*). The low-lying excited states are ordered in a similar way with differences in absolute energies no larger than 0.03 eV. This suggests that complexes **1** and **2** would be expected to have very similar photophysical properties in the gas phase. These computational results appear to be in contradiction to the experimental data because they show that only **2** is capable of thermally accessing its singlet excited state. However, the molecular structures of the two complexes are significantly different in the crystalline state. The structural changes of their Cu^I^_4_ chains through computational geometry optimization for the gas phase are more pronounced in complex **1** than in **2** (*vide supra*). On the other hand, there are smaller structural changes for **1** observed upon photoexcitation in the gas phase (Figures S34 and S35 and Tables S3 and S4). In the S_1_ structure of **1**, the Cu_(out)_–Cu_(in)_–Cu_(in)_′ angles are increased by 12.6 and 14.3°, while **2** shows a more substantial increase of 13.1 and 21.4° in the first excited singlet state. The effect is an induced relaxation toward linearity in both structures. In the T_1_ state, complex **1** adopts a more linear structure in one half of the molecular structure in comparison to the S_0_ ground state, as indicated by an increase of the corresponding Cu_(out)_–Cu_(in)_–Cu_(in)_′ angle of 9.7° (to 169.1°). The clearly more bent ground-state structure of complex **2** becomes distinctly more linear in the T_1_ state (by +20.9° relative to S_0_), effectively showing the same degree of linearity as in **1** (Cu_(out)_–Cu_(in)_–Cu_(in)_′: 168.9°). Due to Jahn–Teller distortion, the second half of both molecular structures of **1** and **2** is evidently more distorted (the Cu_(out)_–Cu_(in)_–Cu_(in)_′ angle for **1** is 149.4 and 133.6° for **2**). The central Cu_(in)_···Cu_(in)_′ distances in **1** and **2** become smaller in the S_1_ and T_1_ states and they are also very similar to each other (for S_1_, **1**: 2.446 Å and **2**: 2.488 Å; for T_1_, **1**: 2.561 Å and **2**: 2.593 Å, Tables S3 and S4).

Overall, upon photoexcitation, gas phase calculations indicate that complex **1** undergoes fewer structural changes than **2**, from a slightly bent ground-state structure to an almost linear arrangement in the S_1_ state. The S_1_ structure of **2** is formed by larger structural rearrangements than in **1** and resembles the S_1_ structure of **1**. For the first triplet excited state, both structures are even more similar to each other. In the gas phase, such structural rearrangements are relatively unconstrained, which leads to similar computed vertical and adiabatic Δ*E*(S_1_–T_1_) values for **1** and **2** in the gas phase calculations. However, in the solid state, intermolecular interactions place steric constraints that prevent the molecules from undergoing large-scale motions, resulting in more linear structures of the S_1_ and T_1_ states of **1** and **2**. An analysis of the available void space of the crystal packings of **1**·1.5C_7_H_8_, **2**·C_7_H_8_, and **3**·5Et_2_O, using spherical probes (radius: 1.2 Å), confirms that **1**·1.5C_7_H_8_ offers appreciably more void space (10.3%) than **2**·C_7_H_8_ (3.7%, Figures S10 and S14).^42^ This is consistent with the hypothesis that molecular motion in the crystal structure of **2**·C_7_H_8_ is more restricted than in **1**·1.5C_7_H_8_. For this reason, there is strong evidence that the origin of the different photophysical properties of **1** and **2** is attributed to the constrained environment of the crystalline state.

In general, Δ*E*(S_1_–T_1_) increases with increasing frontier orbitals overlap. As demonstrated, HOMOs and LUMOs of **1** and **2** are similar in the gas phase, which explains very similar S_1_–T_1_ adiabatic energy separations. In the crystal lattice, complex **1** can more easily accommodate structural changes upon photoexcitation than complex **2**, which has less void space available. Therefore, complex **2** cannot undergo the observed relaxation from a bent to a more linear structure in the crystal packing to the same extent as in the gas phase. This implies a smaller HOMO–LUMO overlap and a reduced S_1_–T_1_ gap for **2** in comparison to **1**, being consistent with the observed experimental Δ*E*(S_1_–T_1_) value for **1**.

To provide computational support to this hypothesis, the molecular structures of **1** and **2** were recalculated with geometric constraints on the copper atoms. Specifically, the three interatomic Cu···Cu separations and the two Cu_(out)_–Cu_(in)_–Cu_(in)_′ angles were constrained to the values of the crystal structure analyses. As a result, for **2**, Δ*E*(S_1_–T_1_) decreased from 1745 cm^–1^ (Figure S33) to 1447 cm^–1^. By contrast, the S_1_–T_1_ energy separation of **1** only moderately increased from 1756 to 1758 cm^–1^. This is conclusive because complex **1** is already almost linear both in solid state and in the gas phase, whereas complex **2** cannot adopt a similar, more linear geometry through molecular motion, which is limited by less available void space in the crystal lattice.

## Conclusions

**Scheme 6:**
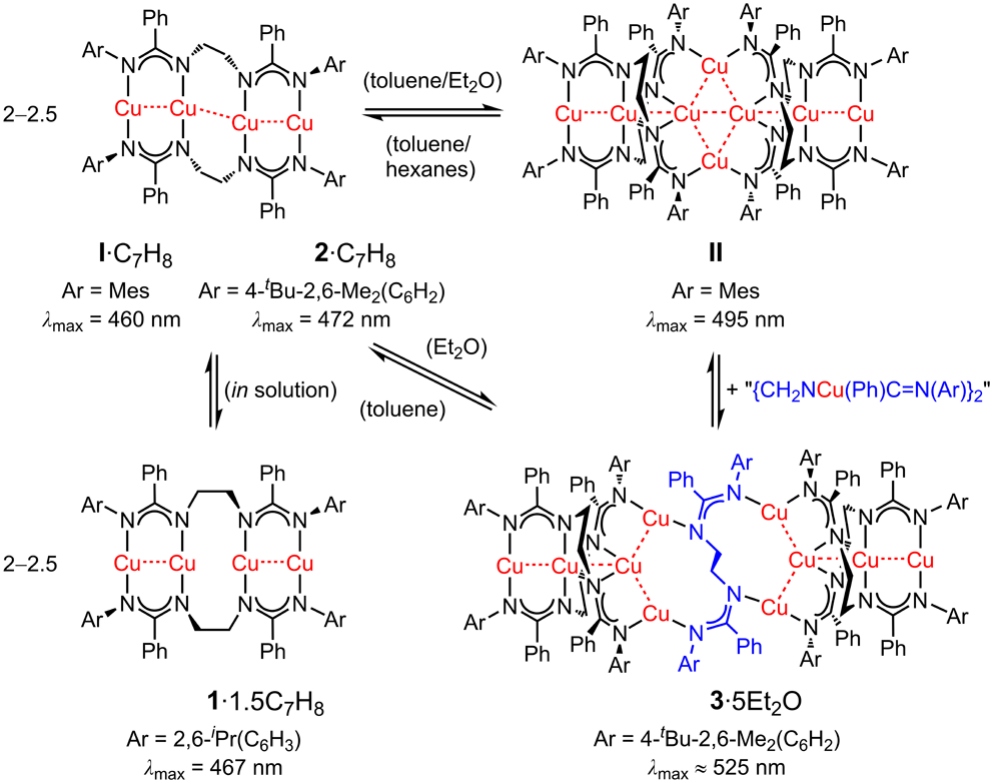
Overview and Interrelation of Complexes **I**, **II**, and **1**–**3**

We have demonstrated that flexible ethylene-bridged bis(amidinates) cleanly convert mesitylcopper into homoleptic Cu^I^ bis(amidinates) that form linear tetranuclear clusters with a disconnected (Cu^I^_(out)_···Cu^I^_(in)_)(Cu^I^_(in)_′···Cu^I^_(out)_′) chain or continuous Cu^I^_4_ chains that are helically bent. Upon crystallization from toluene/diethyl ether mixtures or pure diethyl ether, larger octa- and decanuclear cluster assemblies are formed. This interrelation is shown in Scheme 6. All clusters **I**, **II**, and **1**–**3** are potent blue or green/yellow light emitters in the solid state and exhibit red-shifted emissions with increasing aggregation size.

Based on DFT/TD-DFT calculations for the gas phase, it could be shown that the observed emission of **1** and **2** originates from MLCT transitions. This central Cu^I^_(in)_···Cu^I^_(in)_′ contact, connecting the two discrete Cu^I^_2_ diamidinate segments to a twisted molecular torsion spring, is, in the crystalline state, already present (complex **2**) or, based on the correlation of the TD-DFT calculations with the emission behavior, established upon photoexcitation (complex **1**).

The choice of terminal aromatic substituents in combination with solvent and solvent polarity allows for controlling defined molecular structures through crystallization and consequently adjustable emission properties. These factors not only influence the size of the clusters (and therefore the emission wavelengths) but also the available void space in the crystal lattice (and therefore phosphorescence or TADF behavior).

Future work will be devoted to two major directions: first, control of the aggregation size of the Cu^I^ bis(amidinate) clusters through solubility and solvent polarity. Second, control of the void space in the crystal lattices through ligand design and cocrystallization of suitable solvent molecules that reduce the available void space to promote TADF behavior by reduced molecular motion of the embedded emissive clusters.

## Experimental Section and Computational Details

### General Procedures

All synthetic procedures were carried out by using Schlenk or glovebox techniques under an atmosphere of dry argon. Glassware and NMR tubes were heat-sealed with a heat gun under vacuum. *Caution! Extreme care should be taken both in the handling of the cryogen liquid nitrogen and its use in the Schlenk line trap to avoid the condensation of oxygen from air. Solvents*: prior to use, diethyl ether (EMD, ≥99.0%), hexanes (mixture of isomers, BDH, ≥98.5%), and toluene (BDH, ≥99.5%) were freshly distilled from sodium/benzophenone. Alternatively, the aforementioned solvents were purified using a PPT Solvent Purification System. Dichloromethane (VWR, ≥99.5%) was freshly distilled from CaH_2_. *Deuterated solvents*: DMSO-*d*_6_ (Cambridge Isotope Laboratories, Inc., D, 99.9 + 0.03% v/v tetramethylsilane, TMS) was used as purchased. C_6_D_6_ (Cambridge Isotope Laboratories, Inc., D, 99.5%) was distilled from sodium. *Reactants*: triethylamine (Alfa Aesar, 99%) was distilled from sodium. Ethylene diamine (Acros, 99+%) was dried over molecular sieves (3 Å). 2,6-Diisopropylaniline (Alfa Aesar, 90+%), 2,6-dimethyl-4-^*t*^butylaniline (Matrix Scientific, 97%), benzoic acid (Alfa Aesar, ≥99.5%), *N*,*N′*-carbonyldiimidazole (Oakwood Chemicals), and PCl_5_ (Alfa Aesar, 98%) were used as purchased. *N*,*N′*-1,2-ethanediylbis(benzenecarboximidoyl chloride)^19,21^ and mesitylcopper^35^ were prepared according to literature procedures.^35a^ Elemental analyses were performed by Atlantic Microlab, Inc. Melting points were determined with an SRS (Stanford Research Systems) Digi Melt instrument using open capillaries; values are uncorrected (the heating rate was 2 K/min). NMR measurements were recorded on a Bruker Avance III 400 spectrometer at ambient probe temperatures unless noted at 400.1 MHz (^1^H) and 100.6 MHz (^13^C), respectively. ^13^C NMR resonances were obtained with proton broadband decoupling and referenced to the solvent signals of DMSO-*d*_6_ at 39.5 and C_6_D_6_ at 128.0 ppm (^1^H NMR: 2.50 (DMSO), and 7.15 (benzene), respectively). ^13^C NMR assignments are based on COSY, NOESY, HSQC, and HMBC 2D experiments. Mass-spectrometric analyses were performed on a Bruker Ultraflex II TOF instrument (MALDI), on a Waters Q-Tof API US quadrupole time-of-flight MS system (low-resolution ESI), and on a Thermo Orbitrap Velos Pro MS system (high-resolution ESI). IR spectra were recorded on a PerkinElmer Spectrum One FT-IR spectrometer equipped with a Universal ATR Sampling Accessory.

### General Procedure for the Preparation of **L**^**1**^H_2_ and **L**^**2**^H_2_

A solution of arylamine (**L**^**1**^H_2_: 1.130 g, 6.37 mmol; **L**^**2**^H_2_: 0.980 g, 5.53 mmol) in toluene (15 mL) was added dropwise to a solution of *N*,*N′*-1,2-ethanediylbis(benzenecarboximidoyl chloride) (**L**^**1**^H_2_: 0.973 g, 3.19 mmol; **L**^**2**^H_2_: 0.844 g, 2.77 mmol) in toluene (60 mL) at 0 °C with stirring. The reaction mixture was allowed to warm to room temperature for 30 min, then heated to reflux for 18 h, and subsequently cooled to room temperature. The resulting precipitate was isolated by filtration, washed with toluene (3 × 3 mL), and then suspended in a mixture of CH_2_Cl_2_ (60 mL) and 1 M aqueous solution of NaOH (60 mL). The organic phase was separated, first washed with 1 M aqueous solution of NaOH (2 × 40 mL), subsequently with deionized water (5 × 40 mL), then with brine (2 × 50 mL), and finally dried over anhydrous Na_2_SO_4_. After filtration, the solution was concentrated by rotary evaporation to about 5 mL. Storage at −35 °C overnight resulted in the formation of small colorless crystals. These were isolated by filtration and dried in oil pump vacuum for 18 h.

#### **L**^**1**^H_2_

Yield: 1.138 g, (1.94 mmol, 61%). mp 145.8–146.9 °C; Anal. Calcd for C_40_H_50_N_4_: C, 81.87; H, 8.59; N, 9.55. Found: C, 81.93; H, 8.39; N, 9.59. ^1^H NMR (400.1 MHz, C_6_D_6_): δ 1.00 (s, ≈12H; CH_3_, ^*i*^Pr), 1.24 (s, ≈12H; CH_3_′, ^*i*^Pr), 3.27 (broad s with broad shoulder, ≈5H; CH_2_ and CH, ^*i*^Pr), 3.78 (broad s, ≈3H; CH_2_), 5.13 (broad s, ≈2H; NH), 6.84, 7.06 (2 × s, 16H; CH, Dipp, Ph, overlaid by residual benzene signal), 7.81 (broad, CH, Dipp, Ph). ^1^H NMR (400.1 MHz, DMSO-*d*_6_): δ 0.86 (s, 12H; CH_3_, ^*i*^Pr), 1.06 (s, 12H; CH_3_′, ^*i*^Pr), 3.01 (s, 4H; CH, ^*i*^Pr), 3.36–3.67 (broad m, 4H; CH_2_), 6.79–6.87 (m, 6H; CH, Dipp, Ph), ≈7.04–7.22 (m, 12H; CH, Dipp, Ph, NH). ^13^C{^1^H} NMR (100.6 MHz, C_6_D_6_): δ 22.6 (CH_3_, ^*i*^Pr), 24.3 (CH_3_′, ^*i*^Pr), 28.7 (CH, ^*i*^Pr), 42.9 (CH_2_), 122.9, 123.3, 129.4 (CH, Dipp, Ph, two CH signals are likely overlaid by another signal or too broad to be detected), 135.2 (C, Dipp, Ph), 138.7 (C, *o*-Dipp), 145.7 (C, Dipp, Ph), 154.9 (C, CN_2_). ^13^C{^1^H} NMR (100.6 MHz, DMSO-*d*_6_): δ 22.1 (CH_3_, ^*i*^Pr), 23.7 (CH_3_′, ^*i*^Pr), 27.5 (CH, ^*i*^Pr), 41.2 (broad, CH_2_), 121.2, 122.2, 127.7, 128.8 (CH, Dipp, Ph, one CH signal is overlaid by another signal or too broad to be detected), 134.9 (C, Dipp, Ph), 137.6 (C, *o*-Dipp), 145.7 (C, Dipp, Ph) 154.0 (C, CN_2_). MS (ESI(+)): *m*/*z* (relative intensity): 587 (7) [M + H]^+^, 294 (100) [M + 2H]^2+^. HRMS (ESI(+)): *m*/*z* calcd for C_40_H_50_N_4_ [M + H]^+^, 587.4108; found, 587.4107. IR (neat, cm^–1^): 3368, 3306 (w, ν(N–H)), 3058 (w, ν(CH)), 2958, 2932, 2868 (m, ν(CH)), 1620 (vs), 1600, 1582, 1518 (s), 1494, 1452, 1432, 1392, 1374 (m), 1360 (w), 1312, 1300, 1282, 1260 (m), 1226, 1206, 1178 (w), 1140 (m), 1108, 1098, 1074, 1052, 1024, 1012, 934, 920, 902, 834, 806 (w), 766 (vs), 738 (w), 700 (vs).

#### **L**^**2**^H_2_

Yield: 1.033 g, (1.76 mmol, 64%). mp 158.0–159.3 °C. Anal. Calcd for C_40_H_50_N_4_: C, 81.87; H, 8.59; N, 9.55. Found: C, 81.21; H, 8.48; N, 9.31. ^1^H NMR (400.1 MHz, C_6_D_6_): δ 1.26 (s, 18H; CH_3_, ^*t*^Bu), 2.16 (s, 12H; CH_3_, 4-^*t*^Bu-2,6-*Me*_2_(C_6_H_2_)), 2.99 (broad s, ≈1H; CH_2_), 3.76 (s, ≈3H; CH_2_), 5.43 (broad s, ≈2H; NH), 6.77 (s, 5H; CH, Ph), 7.02 (s, 4H; CH, 4-^*t*^Bu-2,6-Me_2_(C_6_*H*_2_)), ≈7.02–7.15, 7.83 (broad, 10H; CH, Ph, overlaid by C_6_H_6_ signal). ^1^H NMR (400.1 MHz, DMSO-*d*_6_, 60 °C): δ 1.20 (s, 18H; CH_3,_^*t*^Bu), 1.96 (s, 12H; CH_3_, 4-^*t*^Bu-2,6-*Me*_2_(C_6_H_2_)), 3.63 (broad s, 4H; CH_2_), 6.83 (s, 4H; CH, 4-^*t*^Bu-2,6-Me_2_(C_6_*H*_2_)), 7.02 (broad s, 2H; NH), 7.24–7.28 (m, 10H; CH, Ph). ^13^C{^1^H} NMR (100.6 MHz, C_6_D_6_): δ 19.4 (CH_3_, 4-^*t*^Bu-2,6-*Me*_2_(C_6_H_2_)), 31.8 (CH_3_, ^*t*^Bu), 34.0 (C, ^*t*^Bu), 43.1 (CH_2_), 125.1 (CH, (4-^*t*^Bu-2,6-Me_2_(C_6_H_2_)), 127.6 (CH, Ph), ≈128 (CH, Ph, overlaid by C_6_D_6_/residual C_6_H_6_ signals), 129.3 (CH, Ph), 136.2 (C, *i*-Ph, *i*-,*o*-C, 4-^*t*^Bu-2,6-Me_2_(C_6_H_2_)), 143.9 (C, *p*-C, 4-^*t*^Bu-2,6-Me_2_(C_6_H_2_)), 146.1 (C, *i*-Ph, *i*-,*o*-C, 4-^*t*^Bu-2,6-Me_2_(C_6_H_2_)), 156.4 (C, CN_2_) (one quaternary ^13^C resonance is overlaid by another signal or too broad to be detected). ^13^C{^1^H} NMR (100.6 MHz, DMSO-*d*_6_, 60 °C): δ 18.5 (CH_3_, 4-^*t*^Bu-2,6-*Me*_2_(C_6_H_2_)), 31.2 (CH_3_, ^*t*^Bu), 33.3 (C, ^*t*^Bu), 41.1 (CH_2_), 123.9 (CH, (4-^*t*^Bu-2,6-Me_2_(C_6_H_2_)), 127.1, 127.6 (CH, *o*-,*m*-Ph), 129.0 (CH, *p*-Ph), 135.3, 142.9, 144.5 (C, *i*-Ph, 4-^*t*^Bu-2,6-Me_2_(C_6_H_2_)), 155.7 (C, CN_2_). MS (ESI(+)): *m*/*z* (relative intensity): 587 (20) [M + H]^+^, 531 (6) [M – ^*t*^Bu]^2+^, 294 (100) [M + 2H]^2+^. HRMS (ESI(+)): *m*/*z* calcd for C_40_H_50_N_4_ [M + H]^+^, 587.4108; found 587.4103. IR (neat, cm^–1^): 3404 (m, ν(N–H)), 3058, 3051, 3033, 2991 (w, ν(CH)), 2949, 2934, 2920 (m, ν(CH)), 2901, 2869, 2863 (w, ν(CH)), 1635 (vs), 1598 (s), 1576, 1558, 1553 (w), 1505 (s), 1479 (vs), 1461 (m), 1441 (s), 1408, 1391, 1374 (w), 1359 (m), 1323 (w), 1292 (vs), 1231 (w), 1211 (m), 1176 (w), 1147 (m), 1115 (s), 1074, 1038, 1029 (w), 992 (m), 945, 937, 920, 894, 884 (w), 872 (s), 849, 801 (w), 777, 754 (s), 700 (vs).

### General Procedure for the Preparation of **1–3** ([**L**^**1**^Cu_2_]_*n*_, [**L**^**2**^Cu_2_]_*n*_)

To a stirred solution of mesitylcopper (100 mg, 0.547 mmol) in toluene (10 mL) was added a solution of **L**^**1,2**^H_2_ (161 mg, 0.274 mmol) in toluene (10 mL) dropwise at −35 °C by means of a cannula. The reaction mixture was allowed to warm to room temperature and stirred for 1 d at this temperature. In the following, the reaction mixture was filtered through a pad of diatomaceous earth (1 cm × 1 cm). The resulting solution was evaporated to dryness by using oil pump vacuum. The resulting solid was subsequently washed with cold (−35 °C) diethyl ether (3 × 2 mL) and hexanes (3 × 2 mL) and then isolated by filtration. Drying in oil pump vacuum for about 18 h resulted in a colorless ([**L**^**1**^Cu_2_]_*n*_) or a bright citreous powder ([**L**^**2**^Cu_2_]_*n*_). Crystallization of [**L**^**1**^Cu_2_]_*n*_ from toluene yielded **1**·1.5C_7_H_8_. Crystallization of [**L**^**2**^Cu_2_]_*n*_ from toluene yielded **2**·C_7_H_8_ and from diethyl ether **3**·5Et_2_O, respectively (see the crystallographic section for details).

#### [**L**^**1**^Cu_2_]_*n*_

Yield: 134 mg, (0.188 mmol, 69%). mp 240–260 °C (decomposition into a brown oil). Anal. Calcd for C_80_H_96_N_8_Cu_4_: C, 67.48; H, 6.80; N, 7.87. Found: C, 67.15; H, 6.86; N, 6.87. ^1^H NMR (400.1 MHz, C_6_D_6_): δ 1.25 (s, 24H; CH_3_, ^*i*^Pr), 1.26 (s, 24H; CH_3_’, ^*i*^Pr), 3.44 (s, 8H; CH_2_), 3.71 (dq, ^3^*J*_H,H_ = 6.6 Hz, 8H; CH, ^*i*^Pr), 6.68 (t, ^3^*J*_H,H_ = 7.3 Hz, 4H; *p*-Ph), 6.85 (s with broad shoulder, 20H; *m*-Ph, *m*-, *p*-Dipp), 7.05 (broad s, 8H; *o*-Ph). ^13^C{^1^H} NMR (100.6 MHz, C_6_D_6_): δ 22.3 (CH_3_, ^*i*^Pr), 24.7 (CH_3_′, ^*i*^Pr), 28.3 (CH, ^*i*^Pr), 57.7 (C, CH_2_), 123.0 (CH, *m*-Dipp), 124.9 (CH, *p*-Dipp), 127.8–128.5 (3 × CH, *o*-, *m*-, *p*-Ph, overlaid by C_6_D_6_/residual C_6_H_6_ signals), 136.7 (C, *i*-Ph), 142.9 (C, *o*-Dipp), 143.4 (C, *i*-Dipp), 176.7 (C, CN_2_). MS (ESI(+)): *m*/*z* (relative intensity): 1297.7 (0.1) [(**L**^**1**^H_2_)(**L**^**1**^H)Cu_2_]^+^, 1235.7 (5.3) [(**L**^**1**^H)_2_Cu]^+^, 649.3 (6.8) [(**L**^**1**^H)Cu + H]^+^, 587.4 (79.8) [**L**^**1**^H_2_ + H]^+^, 294.2 (100) [**L**^**1**^H_2_ + 2H]^2+^. IR (neat, cm^–1^): 3059, 3026 (w, ν(CH)), 2958 (m, ν(CH)), 2925, 2918, 2888, 2869, 2865, 2860, 2848 (w, ν(CH)), 1603, 1579 (w), 1487, 1430 (vs), 1380, 1359, 1342 (m), 1319, 1257 (s), 1234, 1210 (m), 1178, 1157 (w), 1140 (m), 1096 (vs), 1080, 1076, 1056, 1040 (s), 1026 (vs), 933, 919, 866 (m), 846 (w), 820 (s), 793 (vs), 786 (vs), 767 (s), 753, 745 (m), 726 (s), 700, 693 (vs), 661 (s).

#### [**L**^**2**^Cu_2_]_*n*_

Yield: 127 mg, (0.178 mmol, 68%). mp 240–260 °C (decomposition into a brown oil). Anal. Calcd for C_80_H_96_N_8_Cu_4_: C, 67.48; H, 6.80; N, 7.87. Found: C, 67.33; H, 6.72; N, 7.85. ^1^H NMR (400.1 MHz, C_6_D_6_): δ 1.02 (s, 36H; CH_3_, ^*t*^Bu), 2.65 (s, 24H; CH_3_, 4-^*t*^Bu-2,6-*Me*_2_(C_6_H_2_)), 3.21 (s, 8H; CH_2_), 6.68 (t, ^3^*J*_H,H_ = 7.4 Hz, 4H; *p*-Ph), 6.77–6.81 (m, 8H; *m*-Ph, overlaid by *m*-CH signal, 4-^*t*^Bu-2,6-Me_2_(C_6_*H*_2_)), 6.81 (s, 8H; *m*-CH, 4-^*t*^Bu-2,6-Me_2_(C_6_*H*_2_)), overlaid by *m*-CH signal, Ph), 7.10 (d, ^3^*J*_H,H_ = 7.4 Hz, 8H; *o*-CH, Ph). ^13^C{^1^H} NMR (100.6 MHz, C_6_D_6_): δ 20.5 (CH_3_, 4-^*t*^Bu-2,6-*Me*_2_(C_6_H_2_)), 31.3 (CH_3_, ^*t*^Bu), 33.8 (C, ^*t*^Bu), 55.2 (CH_2_), 125.0 (*m*-CH, 4-^*t*^Bu-2,6-Me_2_(C_6_H_2_)), 126.9 (CH, *o*-Ph), 127.6 (CH, *m*-Ph), 127.6–128.2 (CH, *p*-Ph, overlaid by C_6_D_6_/residual C_6_H_6_ signals), 132.2 (*o*-C, 4-^*t*^Bu-2,6-Me_2_(C_6_H_2_)), 137.2 (C, *i*-Ph), 144.2 (*i*-C, 4-^*t*^Bu-2,6-Me_2_(C_6_H_2_)), 145.7 (*p*-C, 4-^*t*^Bu-2,6-Me_2_(C_6_H_2_)), 176.4 (C, CN_2_). MS (ESI(+)): *m*/*z* (relative intensity): 1359.6 (<0.1) [(**L**^**2**^H)_2_Cu_3_]^+^, 1297.7 (0.3) [(**L**^**2**^H_2_)(**L**^**2**^H)Cu_2_]^+^, 1235.7 (3.6) [(**L**^**2**^H)_2_Cu]^+^, 1173.8 (0.4) [(**L**^**2**^H_2_)_2_ + H]^+^, 649.3 (6.0) [(**L**^**2**^H)Cu + H]^+^, 587.4 (81.5) [**L**^**2**^H_2_ + H]^+^, 531.3 (7.7) [**L**^**2**^H – ^*t*^Bu + H]^+^, 294.2 (100) [**L**^**2**^H_2_ + 2H]^2+^. IR (neat, cm^–1^): 3060, 3050, 3028 (w, ν(CH)), 2961, 2950 (m, ν(CH)), 2932, 2927, 2917, 2903, 2869, 2863, 2849, 2843 (w, ν(CH)), 1604, 1580, 1559 (w), 1514 (s), 1489, 1429 (vs), 1393 (m), 1370 (w), 1361, 1350 (m), 1342 (s), 1304 (m), 1286 (w), 1267, 1250 (m), 1218 (s), 1177 (w), 1153 (m), 1117 (w), 1092 (m), 1073, 1063 (w), 1038 (w), 1027 (m), 989, 953, 943, 921 (w), 873 (m), 862, 849 (w), 805 (m), 780 (w), 765 (s), 728 (m), 703 (vs), 681 (m), 665 (w).

### Photoluminescence Measurements

PL measurements were performed on a Horiba Jobin Yvon Fluorolog-322 spectrometer. Solid samples (crystalline powders) were measured as dispersions in a thin layer of viscous perfluoropolyether oil between two quartz plates. The latter were mounted in one of two closed-cycle optical cryostats (operating temperature ranges: ≈20–300 and 3–300 K). All emission spectra were corrected for the wavelength-dependent response of the spectrometer and detector (in relative photon flux units). Emission decay traces were recorded by connecting a Fluorolog photomultiplier to a fast digital oscilloscope (via a 50, 500, or 2500 Ω load depending on the decay time scale) and using a nitrogen laser for pulsed excitation (337 nm, ∼2 ns, ∼5 *μ*J per pulse). PL quantum yields of **1–3** at ambient temperature were determined using an integrating sphere out of optical PTFE, which was installed into the sample chamber of the spectrometer. The accuracy of the quantum yield measurements is estimated as ±10%. PL of microcrystals of **1–3** under high hydrostatic pressure was recorded on the Fluorolog spectrometer at ambient temperature using a diamond anvil cell (DAC) with ≈0.8 mm anvils (Diacell Ltd.). Perfluoropolyether or mineral oil was applied as a pressure transmitting medium. Similar to PL measurements with the optical cryostat, the emission from DAC was collected at ≈30° relative to the excitation beam. Further experimental details can be found in ref (43).

### X-ray Crystallography

Colorless plates of **L**^**1**^H_2_, tabular yellow plates of **2**·C_7_H_8_, and bright yellow blocks of **3**·5Et_2_O suitable for XRD analysis were obtained from concentrated diethyl ether (**L^1^**H_2_, −35 °C, and **3**·5Et_2_O, room temperature) and toluene (**2**·C_7_H_8_, −35 °C) solutions, respectively. Single crystals of **L**^**2**^H_2_ and **1**·1.5C_7_H_8_ were grown as colorless blocks from a slowly evaporating diethyl ether (**L**^**2**^H_2_) or toluene (**1**·1.5C_7_H_8_) solutions at room temperature. X-ray data for **L**^**1**^H_2_, **L**^**2**^H_2_, **1**·1.5C_7_H_8_, **2**·C_7_H_8_, and **3**·5Et_2_O were collected on a Bruker Venture X-ray diffractometer (Cu Ka radiation, *λ* = 1.54178 Å or Mo Ka radiation, *λ* = 0.71073 Å) by using *ω* and *φ* scans at 100 K (**L**^**1**^H_2_, **L**^**2**^H_2_, **1**·1.5C_7_H_8_, and **3**·5Et_2_O) or 140 K (**2**·C_7_H_8_, Table S1). The integrated intensities for each reflection were obtained by reduction of the data frames with the program APEX3.^44^ Cell parameters were obtained and refined with 41,386 (11,722 unique, **L**^**1**^H_2_), 13,487 (3379 unique, **L**^**2**^H_2_), 9294 (9294 unique, **1**·1.5C_7_H_8_), and 42,319 (9791 unique, **2**·C_7_H_8_) and 764,415 (79,089 unique, **3**·5Et_2_O) reflections, respectively. The integrated data for **L**^**1**^H_2_, **L**^**2**^H_2_, and **2**·C_7_H_8_ and **3**·5Et_2_O were corrected for absorption by using SADABS.^45^ For **1**·1.5C_7_H_8_, TWINABS^46^ was used for integrated data correction as well as to generate hklf4 and hklf5 files, containing the reflection from the major component only (hklf4) and from both components (hklf5). While the hklf4 file was used for structure solution, hklf5 data served for the final least-squares refinement. The structures were solved by direct methods and refined (weighted least-squares refinement on *F*^2^) by using SHELXL.^47^ The hydrogen atoms were placed in idealized positions and refined by using a riding model. Non-hydrogen atoms were refined with anisotropic thermal parameters. For **L**^**2**^H_2_, residual electron peaks indicated the presence of partially occupied and/or disordered solvent molecules which could not be successfully modeled. These were masked in the final refinement cycles for **L**^**2**^H_2_ using the program Olex2.^48^ Solvent molecules in **1**·1.5C_7_H_8_, **2**·C_7_H_8_ and **3**·5Et_2_O were successfully modeled using typical restraints (for example, SADI, SIMU). In **1**·1.5C_7_H_8_, the toluene molecules are disordered by symmetry, with one in 0.5 occupancy disordered about an inversion center, and another in 0.25 occupancy disordered about a 2-fold rotation axis. In **3**·5Et_2_O, the site occupancies of disordered, overlapping solvent molecules, as well as those of some disordered *^t^*Bu groups were refined as free variables. For all structures, the absence of additional symmetry and void was confirmed using PLATON (ADDSYM).^49^

### Computational Details

The crystallographically determined structures of **1**, **2**, and **3** were geometry-optimized using DFT with the PBE1PBE hybrid functional (also known as PBE0).^50^ The choice of functional was motivated by previous computations on Cu(I) systems that indicate reasonable results with such hybrid functionals.^51^ The Cu atoms were described using the 6-311+G* basis set, while C, N, and H atoms were described using 6-31G*. The same basis set was used in our previous report on complexes **I** and **II**.^19^ No symmetry was enforced in the calculations. Frequency calculations were run to compute thermal corrections to the potential energy for the optimized structures.

Singlet and triplet excited state energies were computed for molecules **1** and **2** using the time-dependent DFT (TD-DFT) approach using the same functional and basis set. Excited state geometry optimizations were performed employing the appropriate excited-state gradients. Natural transition orbitals (NTOs) were computed to provide a more compact description of the excitation orbital character.^52^

## Supporting Information Available

Crystallographic data of **L**^**1**^H_2_, **L**^**1**^H_2_, **1**·1.5C_7_H_8_, **2**·C_7_H_8_, and **3**·5Et_2_O; computational details of **1**–**3**; ^1^H and ^13^C{^1^H} NMR spectra of **L**^**1**^H_2_, **L**^**1**^H_2_, and **1–2**; and photoluminescence spectra of **1–3** (PDF)
Computed structure coordinates (ZIP)
