# Supplementary material for: Twisted and Disconnected Chains: Flexible Linear Tetracuprous Arrays and a Decanuclear CuI Cluster as Blue- and Green/Yellow-Light Emitters
Source: Inorg Chem. 2024 Jun 27;63(28):12943–57. doi: 10.1021/acs.inorgchem.4c01646 (PMC11256752; doi:10.1021/acs.inorgchem.4c01646)
Supplement: Supplementary file 1 — ic4c01646_si_001.pdf [file ic4c01646_si_001.pdf]

Supporting Information

Twisted and Disconnected Chains: Flexible Linear Tetracuprous Arrays and a Decanuclear Cu^{I} Cluster as Blue- and Green/Yellow-Light Emitters

Janet Arras,[a] Alvaro Calderón-Díaz,[a] Sergei Lebedkin,[b] Samer Gozem,[c] Colin D. McMillen,[d] Nattamai Bhuvanesh,[e] and Michael Stollenz*,[a]

a) Department of Chemistry and Biochemistry, Kennesaw State University, 370 Paulding Avenue NW, MD#1203, Kennesaw, Georgia 30144, USA. b) Institute of Nanotechnology, Karlsruhe Institute of Technology (KIT), Herrmann-von-Helmholtz-Platz 1, 76344 Eggenstein-Leopoldshafen, Germany. c) Department of Chemistry, Georgia State University, 145 Piedmont Ave SE, Atlanta, Georgia 30303, P. O. Box 3945, Atlanta, Georgia 30302-3945, USA. d) Department of Chemistry, Clemson University, 379 Hunter Laboratories, Clemson, SC 29634-0973, USA. e) Department of Chemistry, Texas A&M University, P.O. Box 30012, College Station, Texas 77842-3012, USA.

*E-mail: Michael.Stollenz@kennesaw.edu.

Table of Contents

Figures S1–S19. Crystallographic structure representations[S1].. S2
Tables S1/S2. Crystal data and refinement details for **L**$^1H_2$, **L**$^2H_2$, **1**·$1.5C_7H_8$, **2**·C_7H_8, and **3**·$5Et_2O$.. S24
Figures S20–S22. Overlay representations of XRD and geometry-optimized structures.................. S26
Figures S23–S31. Isodensity plots of molecular orbitals for **1–3**.. S28
Figure S32. NTO pairs for the ground-state optimized structures of **1–3**................................ S37
Figure S33. Schematic representation of the electronic states of **1** and **2** as harmonic potentials........ S38
Figures S34/S35. Overlay representations of the geometry-optimized $S_0/S_1/T_1$ structures of **1** and **2**.. S39
Tables S3/S4. Selected calculated and measured distances and angles for **1** and **2**.................... S40
Figures S36–S47. NMR spectra of **L**$^1H_2$, **L**$^2H_2$, and **1–3**..S42
Figures S48/S49. Emission decay traces of **1**·$1.5C_7H_8$ and **2**·C_7H_8..S48
Figure S50. Temperature-dependent PL emission and excitation spectra of **2** after vacuum drying.....S50
Figure S51. Emission decay traces of **2**·after vacuum drying..S51
Figure S52. Emission decay traces of **3**·$5Et_2O$..S52
Figures S53–S55. PL emission/excitation spectra/decay traces of **1**, **2**, **3**·$5Et_2O$ under high pressure. S53
Table S5. Photophysical parameters for polycrystalline complexes **1**·$1.5C_7H_8$, **2**·C_7H_8, and **3**·$5Et_2O$. S56
References and Footnotes...S57

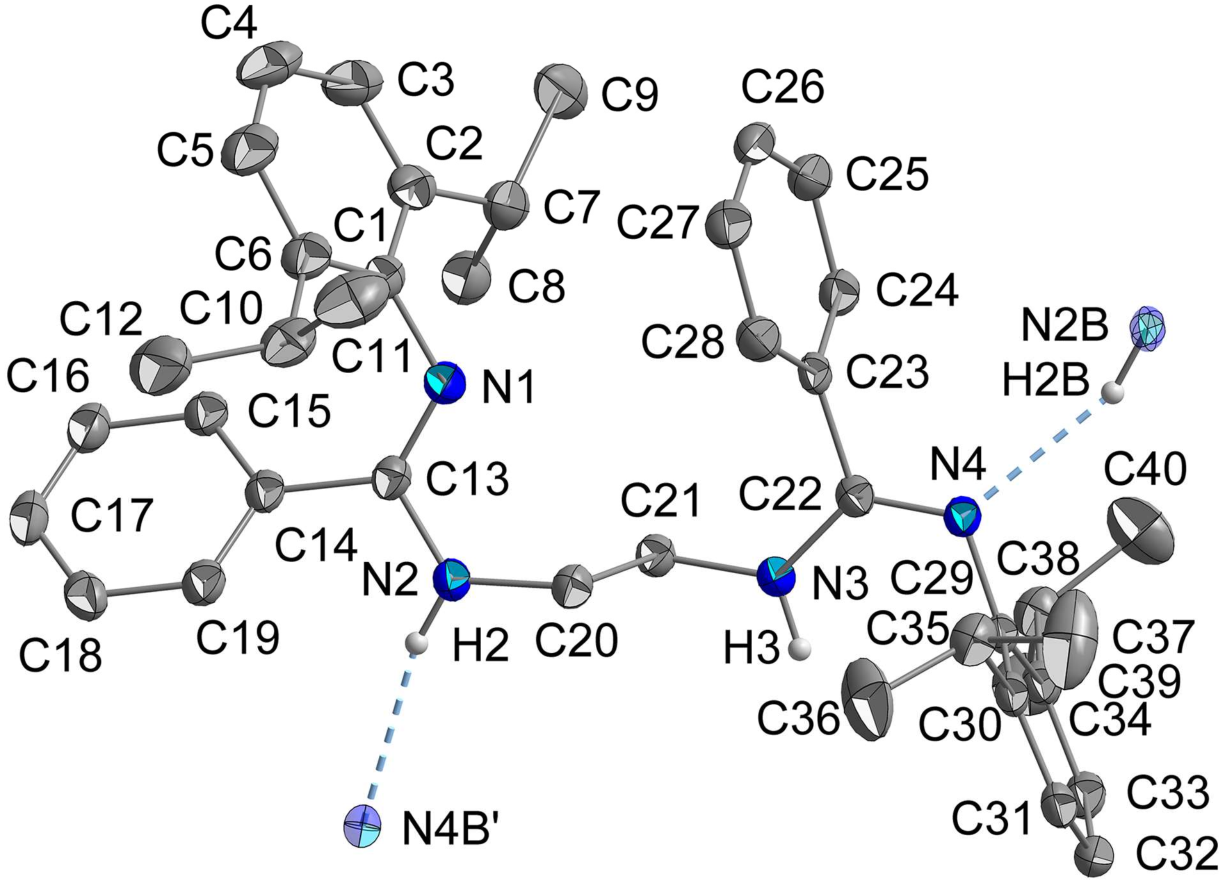

Figure S1: Molecular structure of $\mathbf{L^1}H_2$ showing ellipsoids with anisotropic displacement factors of 50% probability. Hydrogen atoms except for NH functionalities have been omitted for clarity. Selected interatomic distances (Å), bond angles (deg), and torsion angles (deg): C20–C21 1.519(3), N2–C20 1.454(2), N2–C13 1.366(2), N1–C13 1.290(3), N1–C1 1.416(2), N3–C21 1.467(2), N3–C22 1.354(2), N4–C22 1.294(2), N4–C29 1.426(2), N2–C20–C21 112.53(15), C13–N2–C20 121.92(16), N1–C13–N2 119.47(17), C1–N1–C13 121.22(17), N3–C21–C20 111.16(15), C22–N3–C21 126.83(15), N4–C22–N3 125.58(17), C29–N4–C22 118.46(16), N2–C20–C21–N3 –173.15(15), C13–N2–C20–C21 –84.8(2), N1–C13–N2–C20 6.0(3), C22–N3–C21–C20 –102.8(2), N4–C22–N3–C21 –176.70(17). Hydrogen bonds (Å) and associated angles (deg): N2···N4B' 3.235(2), N2B···N4 3.110(2), H2···N4B' 2.37, H2B···N4 2.28, N2–H2···N4B' 167.7, N2B–H2B···N4 157.3. Symmetry operation used to generate equivalent atoms: (') $x - 1$, y, z.

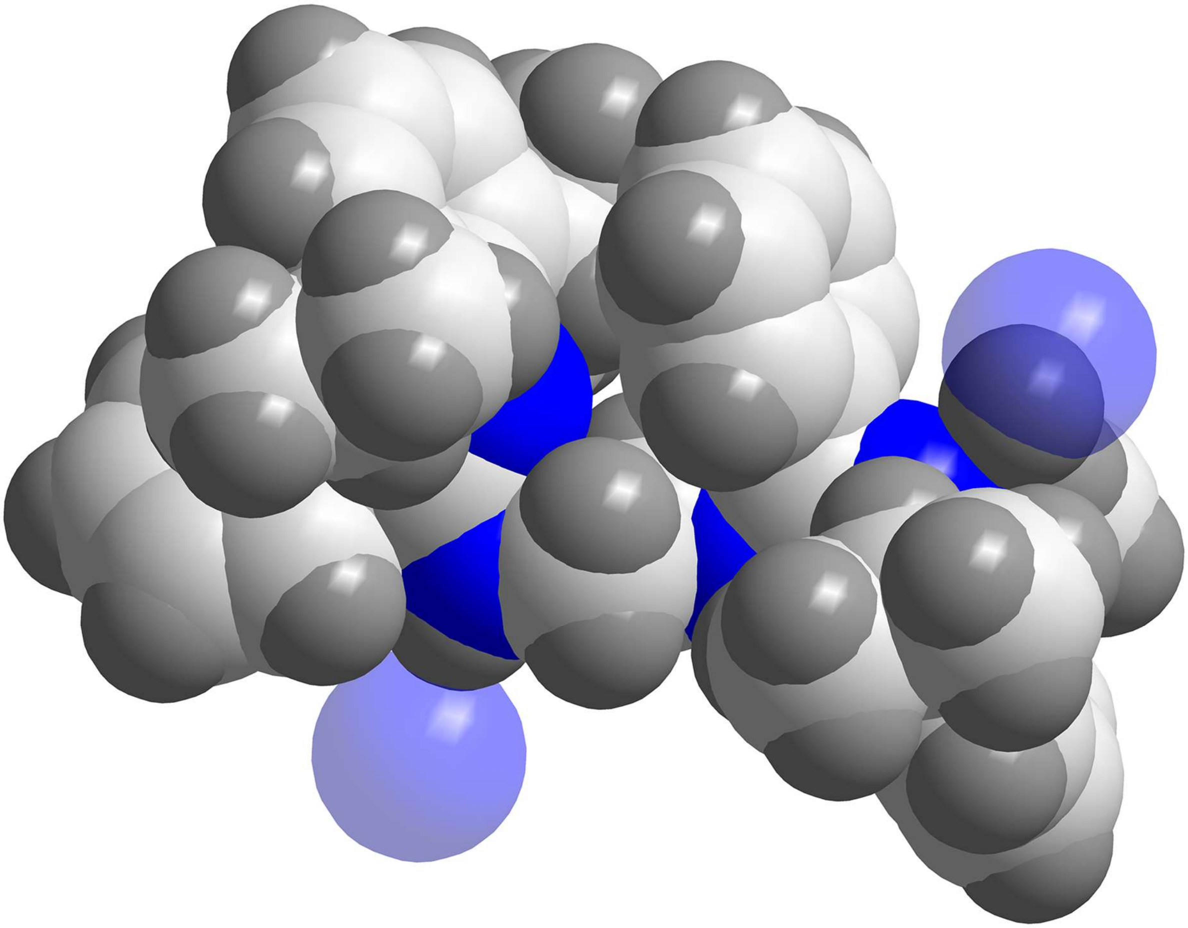

Figure S2: Space filling representation of the molecular structure of $\mathbf{L^1}H_2$.

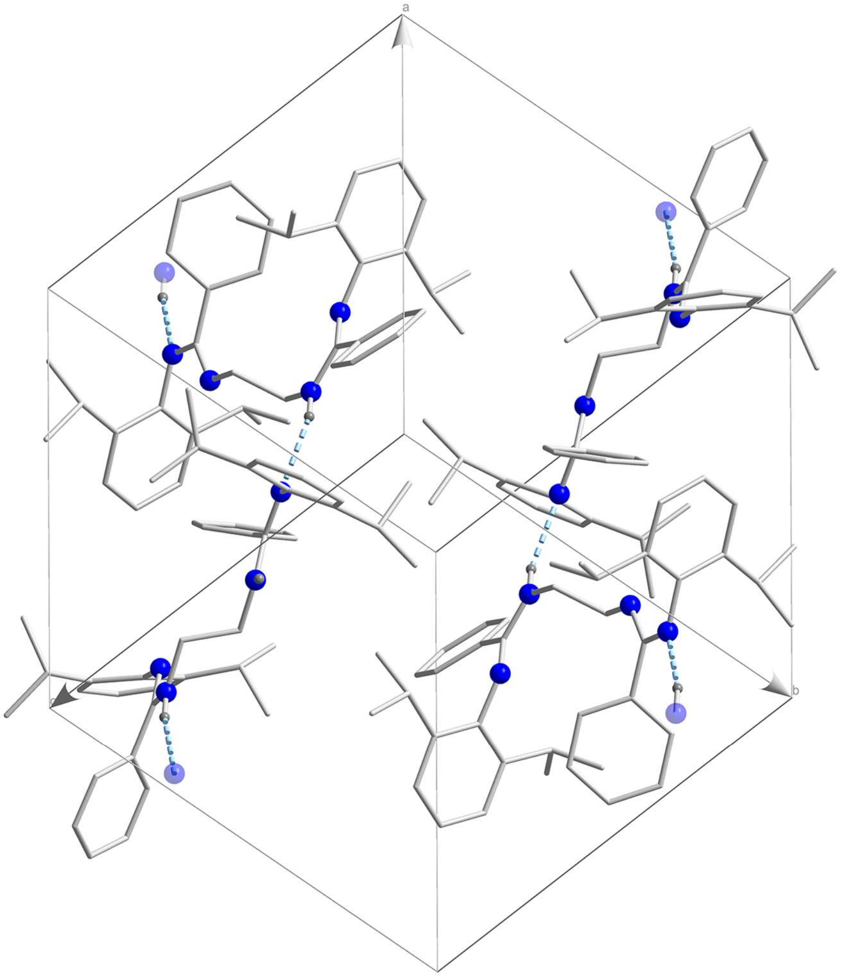

Figure S3: Crystal packing diagram and unit cell of $\mathbf{L^1}H_2$. Hydrogen atoms except for NH functionalities have been omitted for clarity.

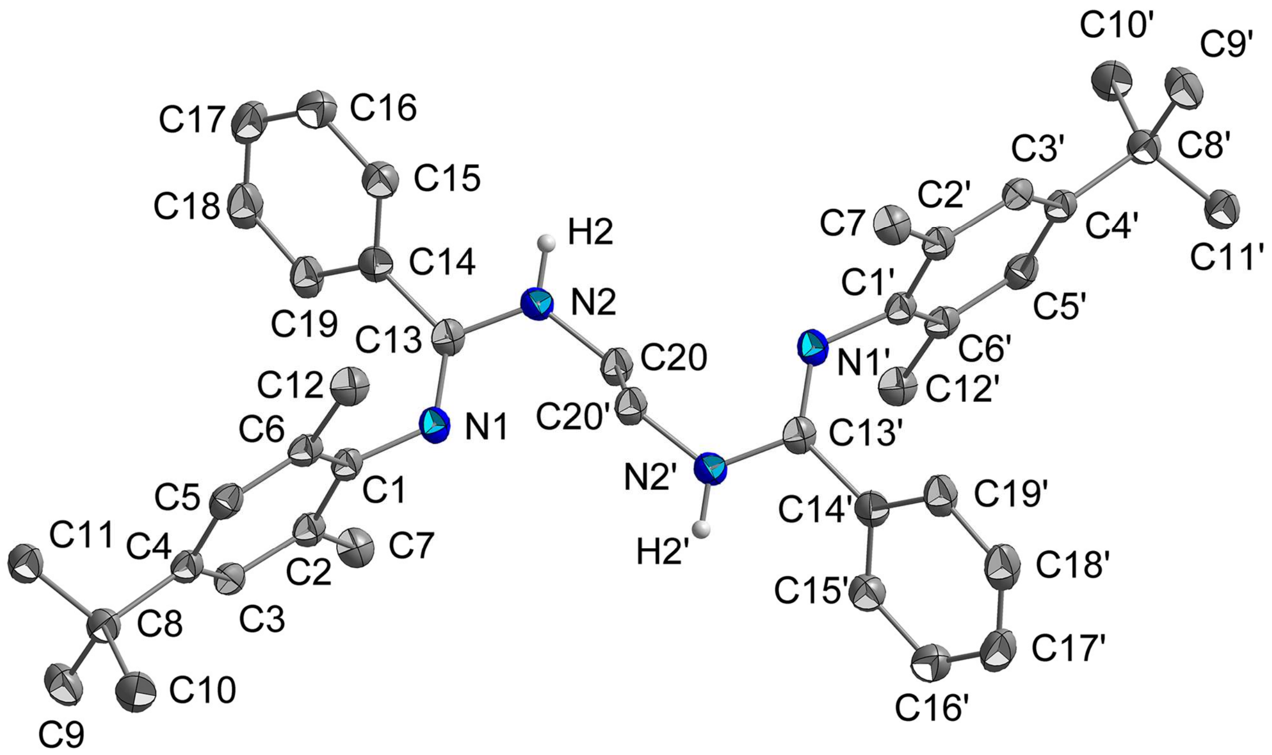

Figure S4: Molecular structure of $\mathbf{L^2}H_2$ showing ellipsoids with anisotropic displacement factors of 50% probability. Hydrogen atoms except for NH functionalities have been omitted for clarity. Selected interatomic distances (Å), bond angles (deg), and torsion angles (deg): C20–C20' 1.527(3), N2–C20 1.457(2), N2–C13 1.366(2), N1–C13 1.283(2), N1–C1 1.412(2), N2–C20–C20' 111.64(18), C13–N2–C20 121.10(14), N1–C13–N2 119.29(15), C1–N1–C13 123.67(14), N2–C20–C20'–N2' 180.0(1),[S2] C13–N2–C20–C20' –74.9(2), N1–C13–N2–C20 5.3(2). Symmetry operation used to generate equivalent atoms: (') $-x + 5/3$, $-y + 4/3$, $-z + 1/3$.

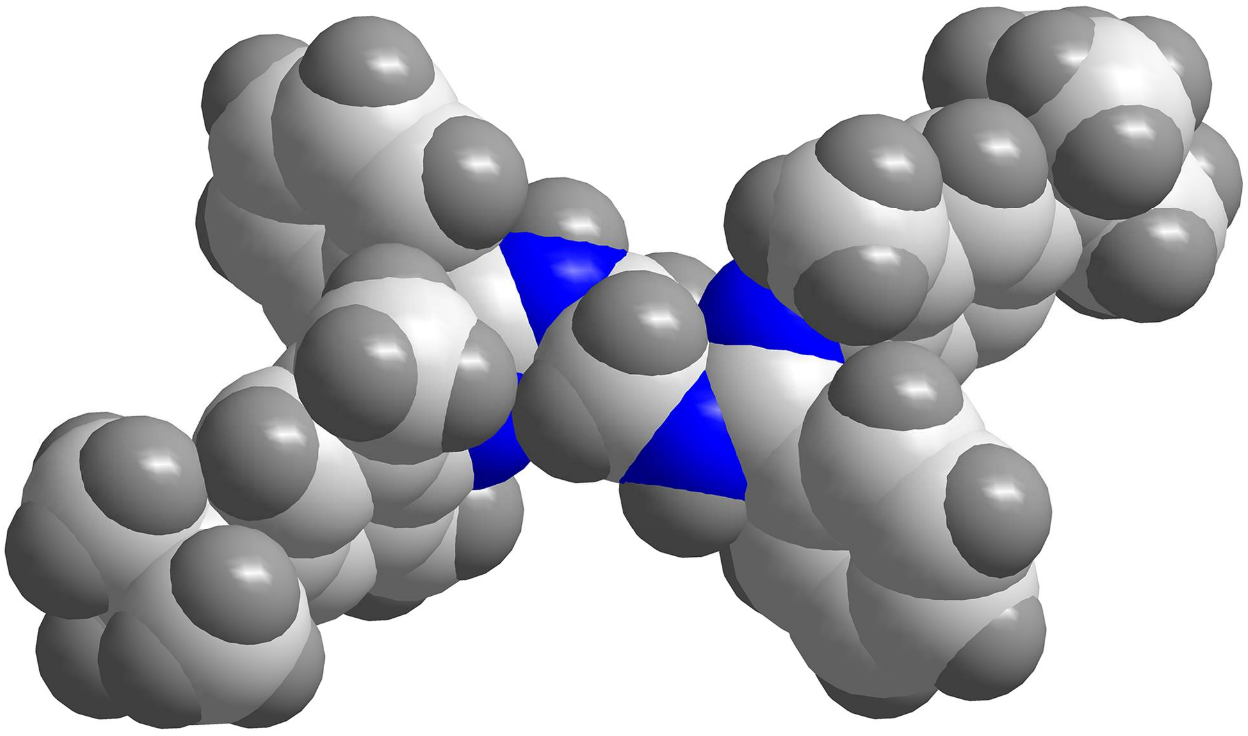

Figure S5: Space filling representation of the molecular structure of $\mathbf{L^2}H_2$. Hydrogen atoms except for NH functionalities have been omitted for clarity.

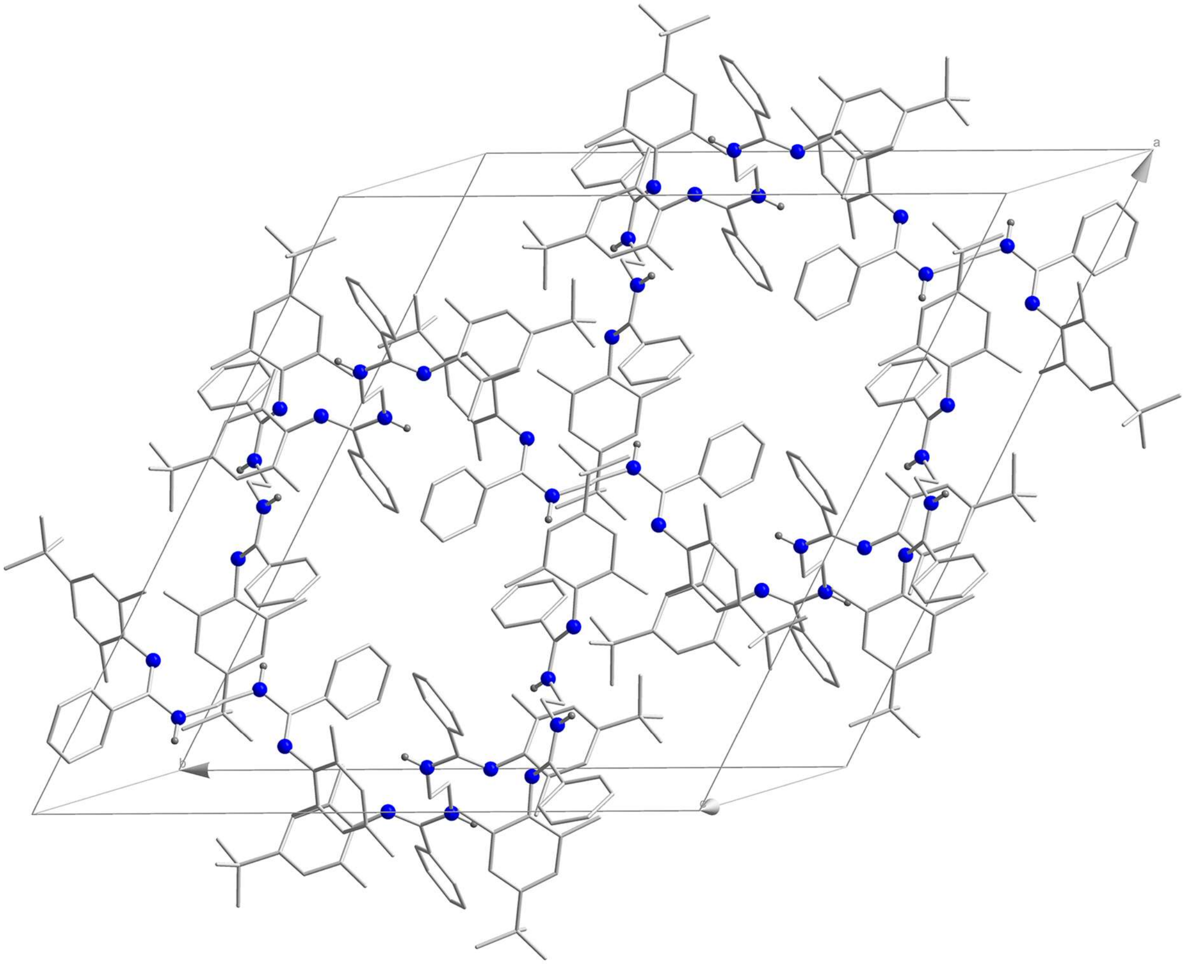

Figure S6: Crystal packing diagram and unit cell of $\mathbf{L^2}H_2$. Hydrogen atoms except for NH functionalities have been omitted for clarity.

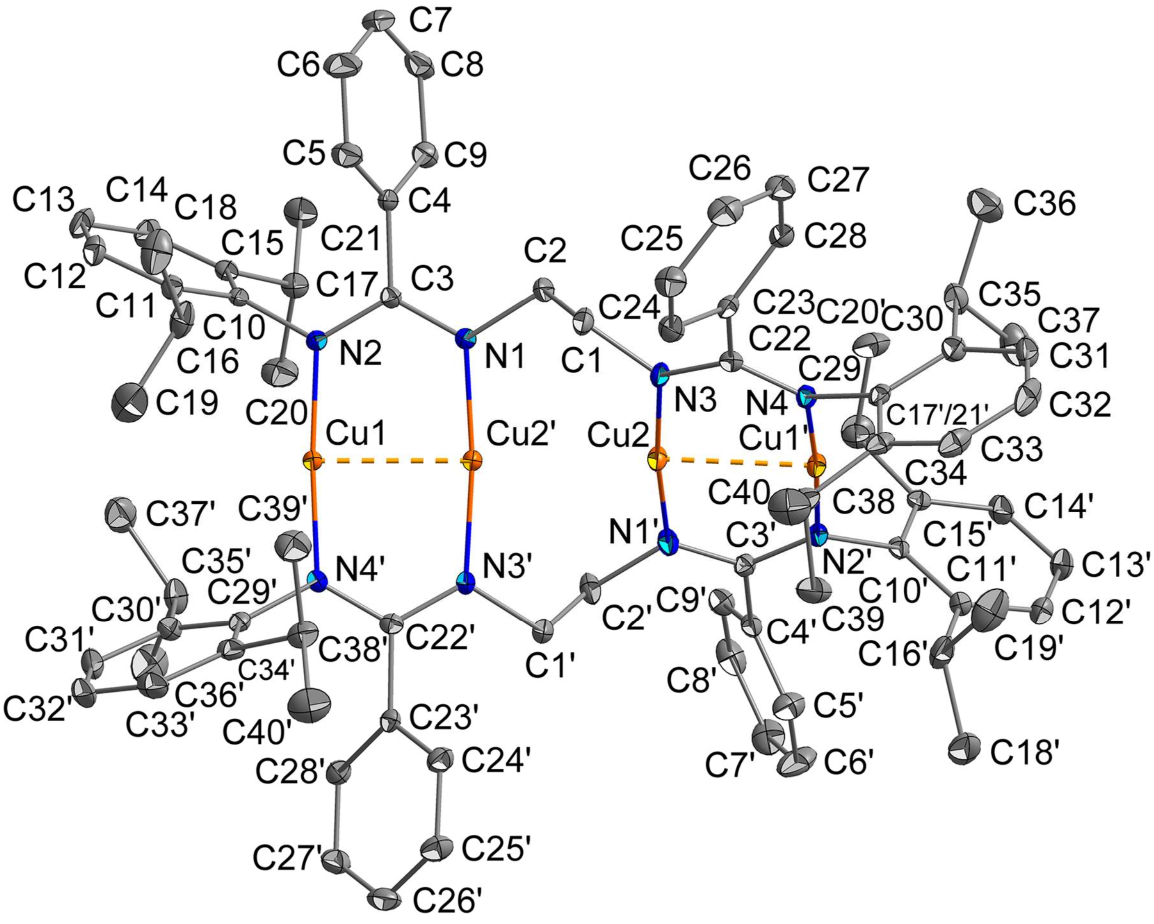

Figure S7: Molecular structure of **1** (P (Δ) enantiomer) showing ellipsoids with anisotropic displacement factors of 50% probability. Hydrogen atoms have been omitted for clarity. Selected interatomic distances (Å), bond angles (deg), and torsion angles (deg): Cu1···Cu2' 2.4771(4), Cu2···Cu2' 2.8702(6), Cu1–N4' 1.870(2), Cu1–N2 1.8648(19), Cu2–N3 1.894(2), Cu2'–N1 1.888(2), C2–C1 1.532(4), N1–C2 1.473(3), C3–N1 1.333(3), N2–C3 1.336(3), C10–N2 1.434(3), N3–C1 1.466(3), C22–N3 1.330(3), N4–C22 1.333(3), C29–N4 1.431(3), Cu1–Cu2'–Cu2 176.400(15), N4'–Cu1–Cu2' 87.80(6), N2–Cu1–Cu2' 87.94(6), N4'–Cu1–N2 175.67(9), Cu1–N4'–C22' 121.62(16), Cu1–N2–C3 121.66(17), Cu1–N4'–C29' 116.06(16), Cu1–N2–C10 117.58(15), N3'–Cu2'–Cu1 86.84(6), N1–Cu2'–Cu1 86.85(6), N3'–Cu2'–N1 173.69(9), N3'–Cu2'–Cu2 93.91(6), N1–Cu2'–Cu2 92.40(6), Cu2'–N3'–C22' 121.20(17), Cu2'–N1–C3 121.71(16), Cu2'–N3'–C1' 118.79(16), Cu2'–N1–C2 118.72(16), N3–C1–C2 111.9(2), C22–N3–C1 119.9(2), N4–C22–N3 121.1(2), C29–N4–C22 121.7(2), N1–C2–C1 111.9(2), C3–N1–C2 119.3(2), N2–C3–N1 120.8(2), C10–N2–C3 120.5(2), N4'–Cu1–Cu2'–N3' 8.8(1),[S2] N4'–Cu1–Cu2'–N1 –171.4(1),[S2] N3'–Cu2'–Cu2–N3 –135.9(1),[S2] N1–Cu2'–Cu2–N1' –136.2(1),[S2] Cu1–N4'–C29'–C34' –102.6(2),[S2] Cu1–N4'–C29'–C30' 72.9(3),[S2] Cu1–N2–C10–C11 74.6(3),[S2] Cu1–N2–C10–C15 –100.7(2),[S2] N1–C2–C1–N3 103.2(2),[S2] C3–N1–C2–C1 120.2(3),[S2] C22–N3–C1–C2 133.7(2).[S2] Symmetry operation used to generate equivalent atoms in the crystal packing (P (Δ) enantiomer): $-x+1, -y+1, -z+1$ and ('): $x, -y+1, z+1/2$.

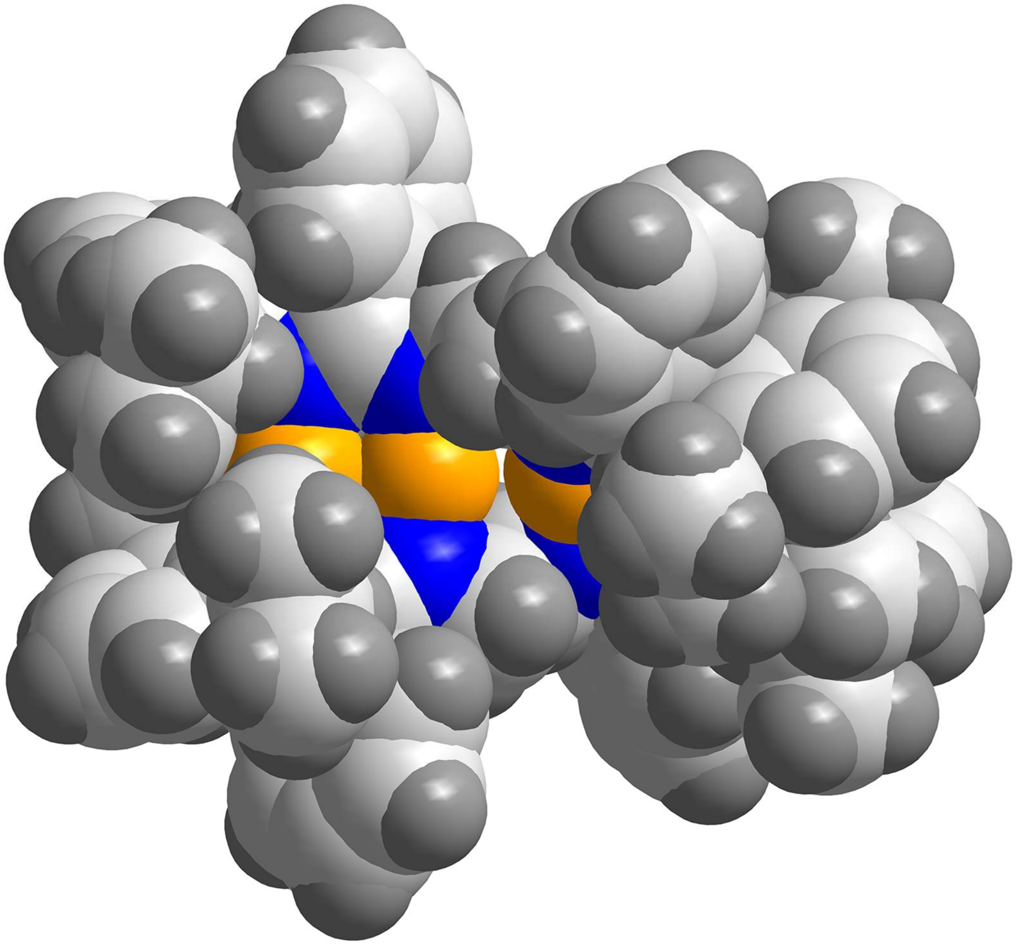

Figure S8: Space filling representation of the molecular structure of **1** (P (Δ) enantiomer).

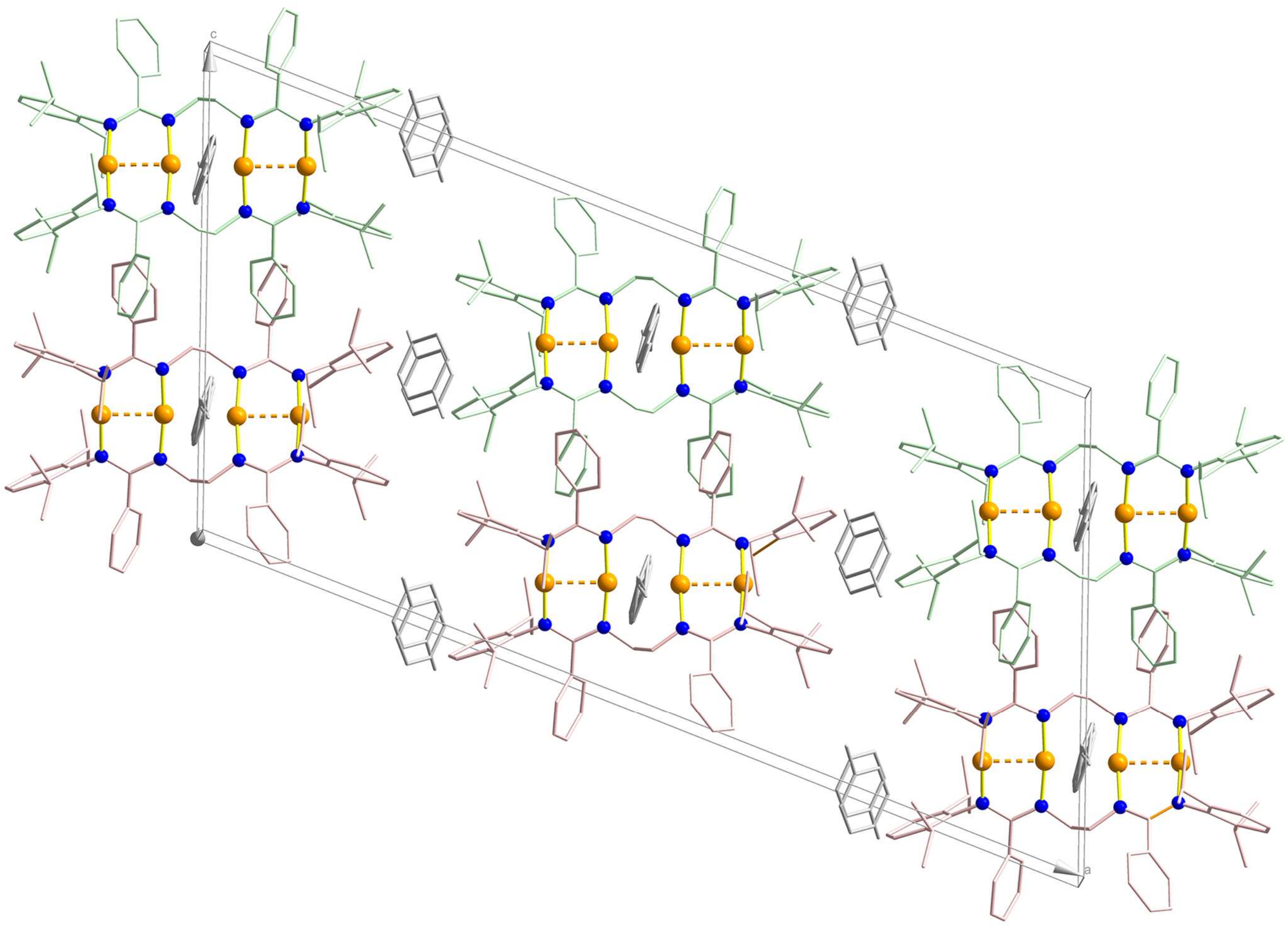

Figure S9: Crystal packing diagram and unit cell of **1**·1.5C_7H_8 showing a racemate consisting of *P*(Δ, green)- and *M*(Λ, salmon)-helices. Hydrogen atoms have been omitted for clarity.

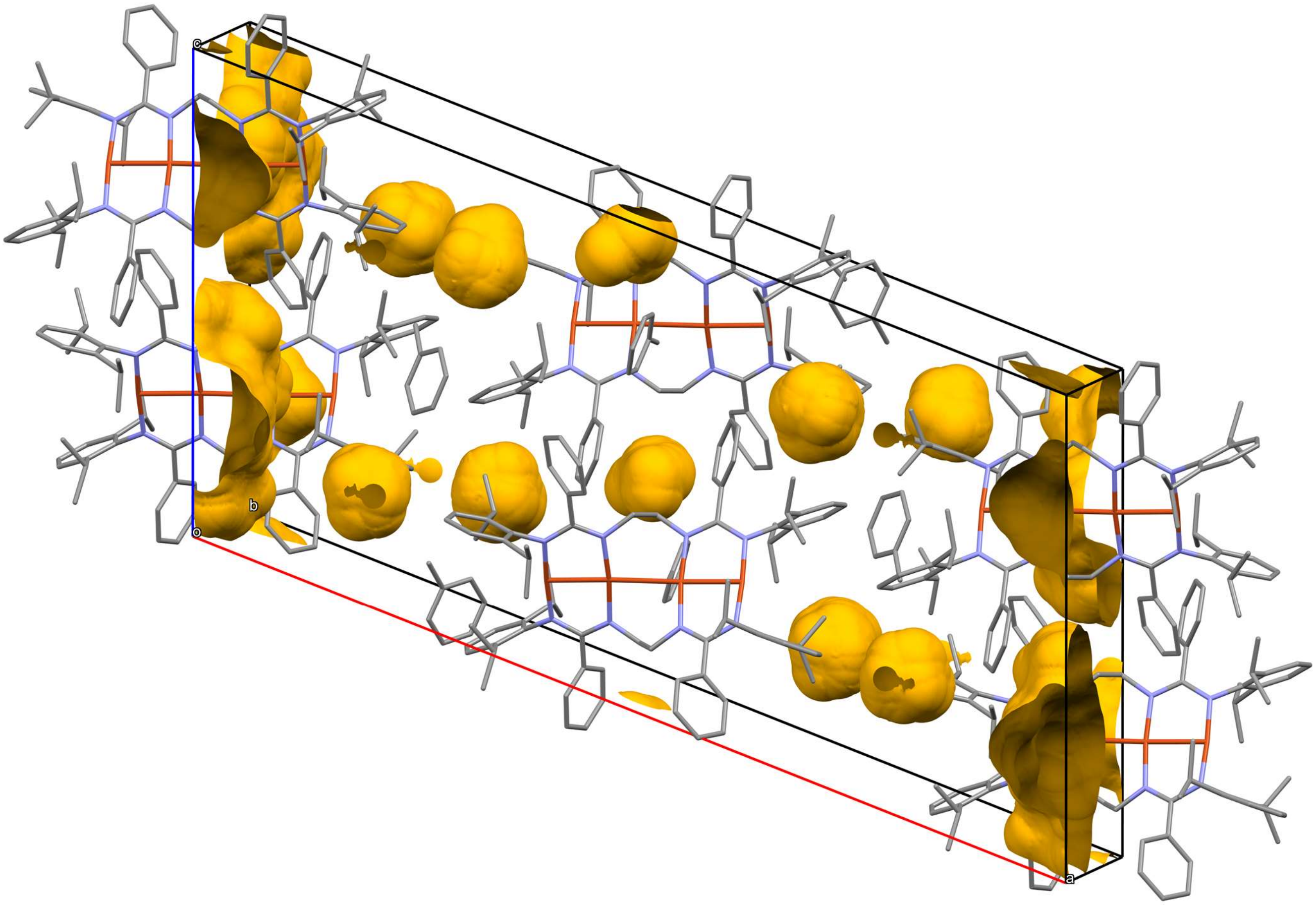

Figure S10: Crystal packing diagram and unit cell of **1**·$1.5C_7H_8$ showing void spaces (spherical probe radius: 1.2 Å).[S3] Hydrogen atoms have been omitted for clarity.

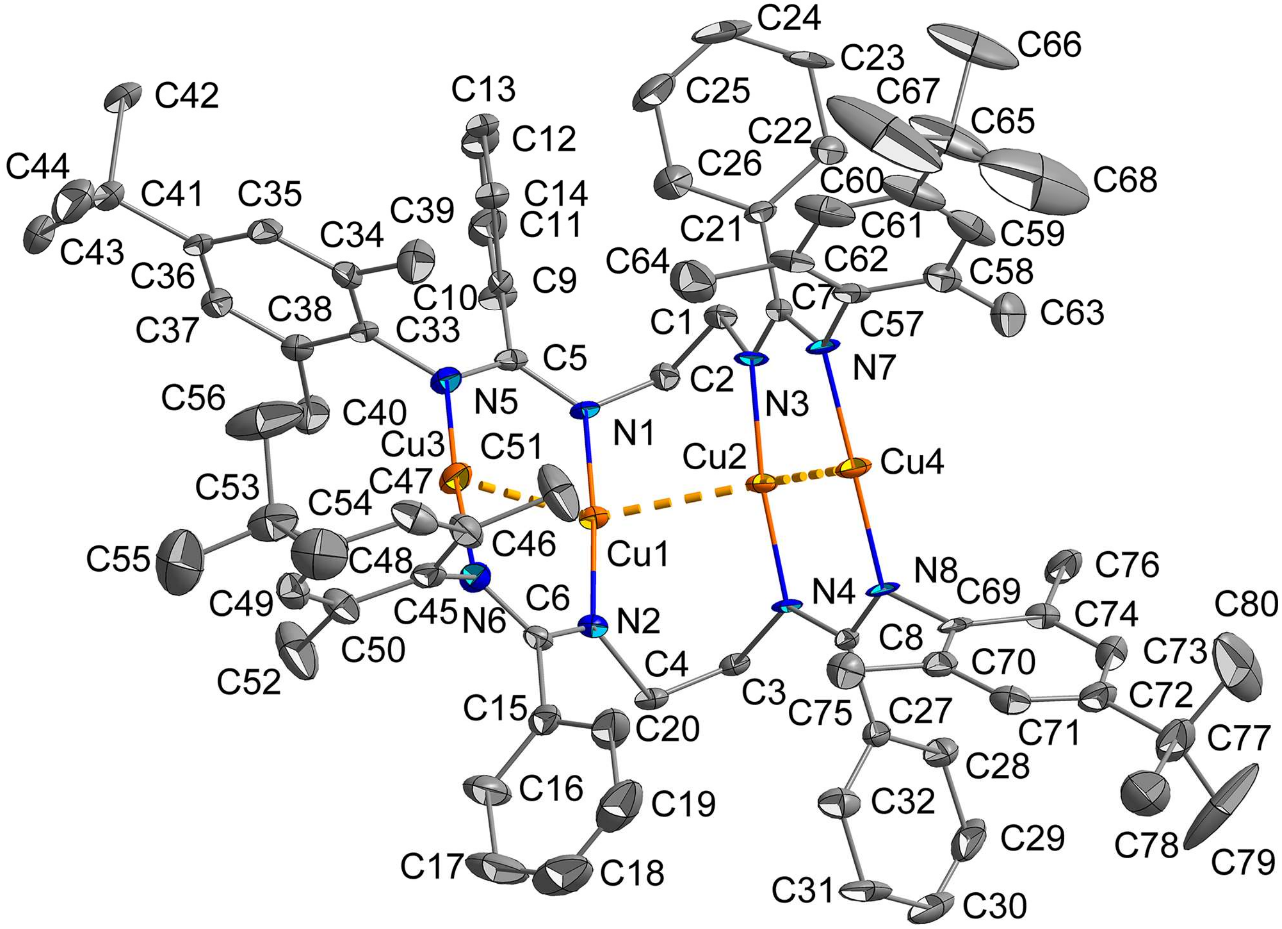

Figure S11: Molecular structure of **2** (*P* (Δ) enantiomer) showing ellipsoids with anisotropic displacement factors of 50% probability. Hydrogen atoms have been omitted for clarity. Selected interatomic distances (Å), bond angles (deg), and torsion angles (deg): Cu3···Cu1 2.4398(9), Cu4···Cu2 2.4579(9), Cu1···Cu2 2.6464(9), Cu3–N5 1.870(4), Cu3–N6 1.858(4), Cu1–N1 1.867(4), Cu1–N2 1.875(4), C3–C4 1.518(7), N2–C4 1.469(6), C6–N2 1.324(6), N6–C6 1.341(6), C45–N6 1.443(7), N4–C3 1.468(6), C8–N4 1.326(6), N8–C8 1.327(6), C69–N8 1.446(6), Cu3–Cu1–Cu2 131.37(3), N5–Cu3–Cu1 88.30(13), N6–Cu3–Cu1 86.50(13), N5–Cu3–N6 174.78(19), Cu3–N5–C5 121.1(3), Cu3–N6–C6 123.3(4), Cu3–N5–C33 117.1(3), Cu3–N6–C45 114.3(3), N1–Cu1–Cu3 87.62(13), N2–Cu1–Cu3 88.52(13), N1–Cu1–N2 175.50(18), N1–Cu1–Cu2 90.57(12), N2–Cu1–Cu2 93.72(13), Cu1–N1–C5 121.4(3), Cu1–N2–C6 120.5(4), Cu1–N1–C2 117.6(3), Cu1–N2–C4 119.4(3), N1–C2–C1 113.7(4), C5–N1–C2 120.6(4), N5–C5–N1 120.6(5), C33–N5–C5 121.7(4), N3–C1–C2 113.8(4), C7–N3–C1 120.9(4), N7–C7–N3 121.0(5), C57–N7–C7 120.2(4), Cu4–N8 1.884(4), Cu4–N7 1.879(4), Cu2–N4 1.880(4), Cu2–N3 1.879(4), C2–C1 1.507(7), N3–C1 1.473(7), C7–N3 1.326(6), N7–C7 1.337(7), C57–N7 1.434(7), N1–C2 1.461(6), C5–N1 1.344(6), N5–C5 1.326(6), C33–N5 1.432(6), Cu4–Cu2–Cu1 137.41(4), N8–Cu4–Cu2 88.18(13), N7–Cu4–Cu2 86.27(13), N8–Cu4–N7 173.60(19), Cu4–N8–C8 121.0(3), Cu4–N7–C7 122.7(3), Cu4–N8–C69 119.4(3), Cu4–N7–C57 117.1(3), N4–Cu2–Cu4

87.00(13), N3–Cu2–Cu4 89.03(13), N4–Cu2–N3 173.28(18), N4–Cu2–Cu1 90.52(13), N3–Cu2–Cu1 96.07(13), Cu2–N4–C8 122.1(3), Cu2–N3–C7 119.8(4), Cu2–N4–C3 117.2(3), Cu2–N3–C7 119.8(4), N4–C3–C4 112.0(4), C8–N4–C3 120.6(4), N8–C8–N4 120.8(4), C69–N8–C8 118.9(4), N2–C4–C3 112.2(4), C6–N2–C4 120.1(4), N6–C6–N2 119.4(5), C45–N6–C6 121.5(4), N5–Cu3–Cu1–N1 –7.2(2),[S2] N5–Cu3–Cu1–N2 170.5(2),[S2] N1–Cu1–Cu2–N4 151.8(2),[S2] N2–Cu1–Cu2–N3 151.7(2),[S2] Cu3–N5–C33–C38 98.1(5),[S2] Cu3–N5–C33–C34 –75.6(5),[S2] Cu3–N6–C45–C50 –86.0(5),[S2] Cu3–N6–C45–C46 84.8(5),[S2] N1–C2–C1–N3 –58.7(6),[S2] C5–N1–C2–C1 –81.7(6),[S2] C7–N3–C1–C2 161.2(5),[S2] N8–Cu4–Cu2–N4 5.7(2),[S2] N8–Cu4–Cu2–N3 –168.9(2),[S2] Cu4–N8–C69–C74 –83.5(5),[S2] Cu4–N8–C69–C70 93.1(5),[S2] Cu4–N7–C57–C58 67.9(5),[S2] Cu4–N7–C57–C62 –107.9(5),[S2] N4–C3–C4–N2 58.8(5),[S2] C8–N4–C3–C4 86.5(6),[S2] C6–N2–C4–C3 –154.1(5).[S2]

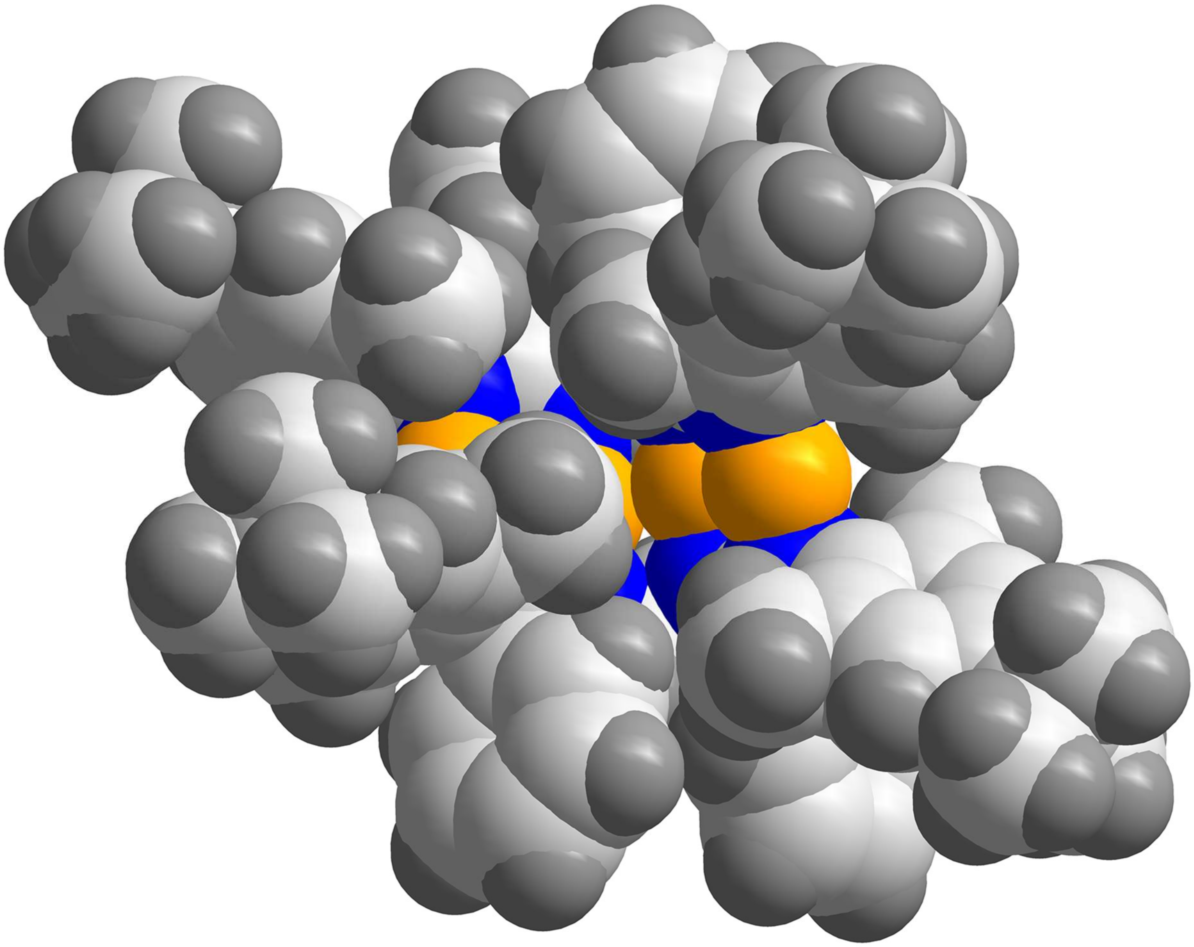

Figure S12: Space filling representation of the molecular structure of **2** (*P* (Δ) enantiomer).

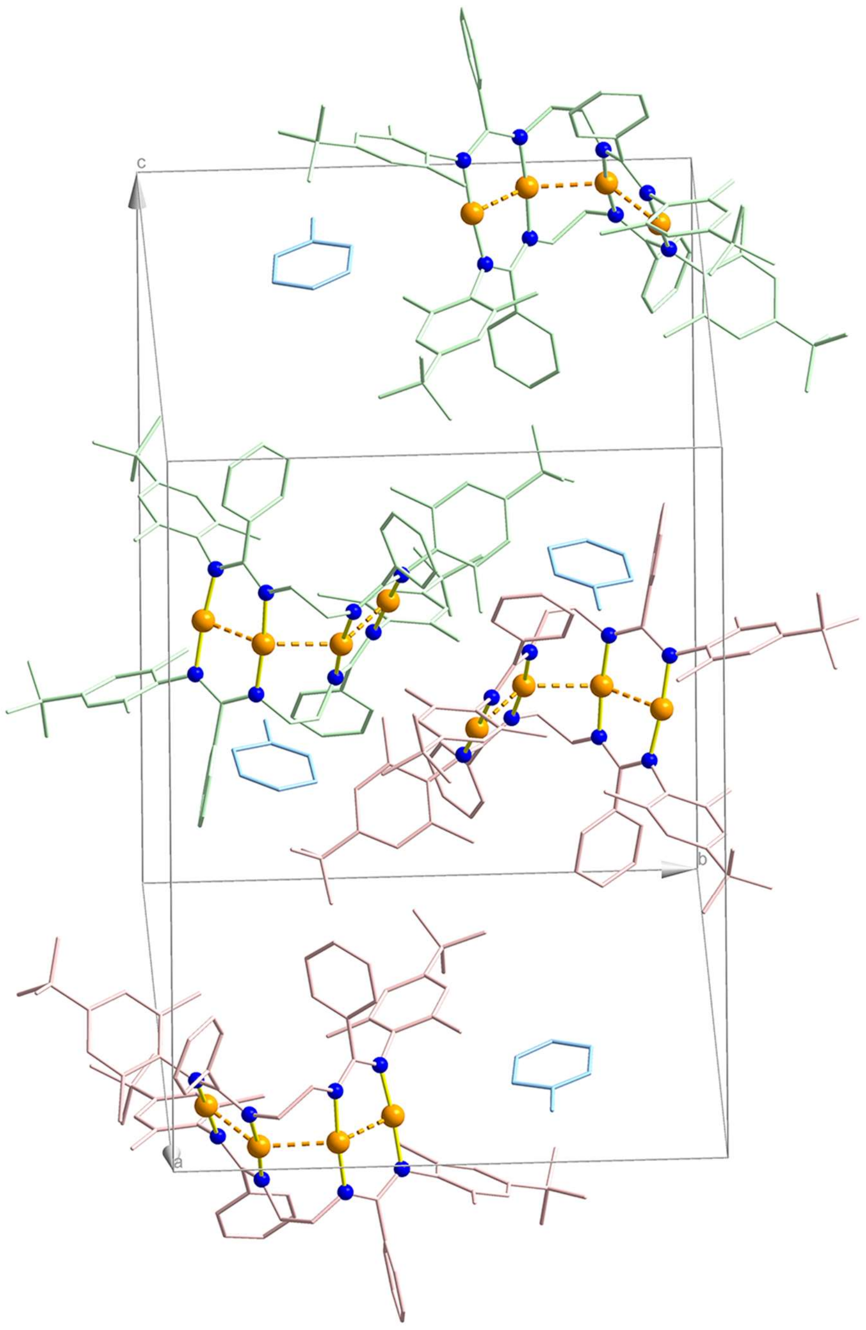

Figure S13: Crystal packing diagram and unit cell of **2**·C_7H_8 showing a racemate consisting of *P*(Δ, green)- and *M*(Λ, salmon)-helices. Hydrogen atoms have been omitted for clarity.

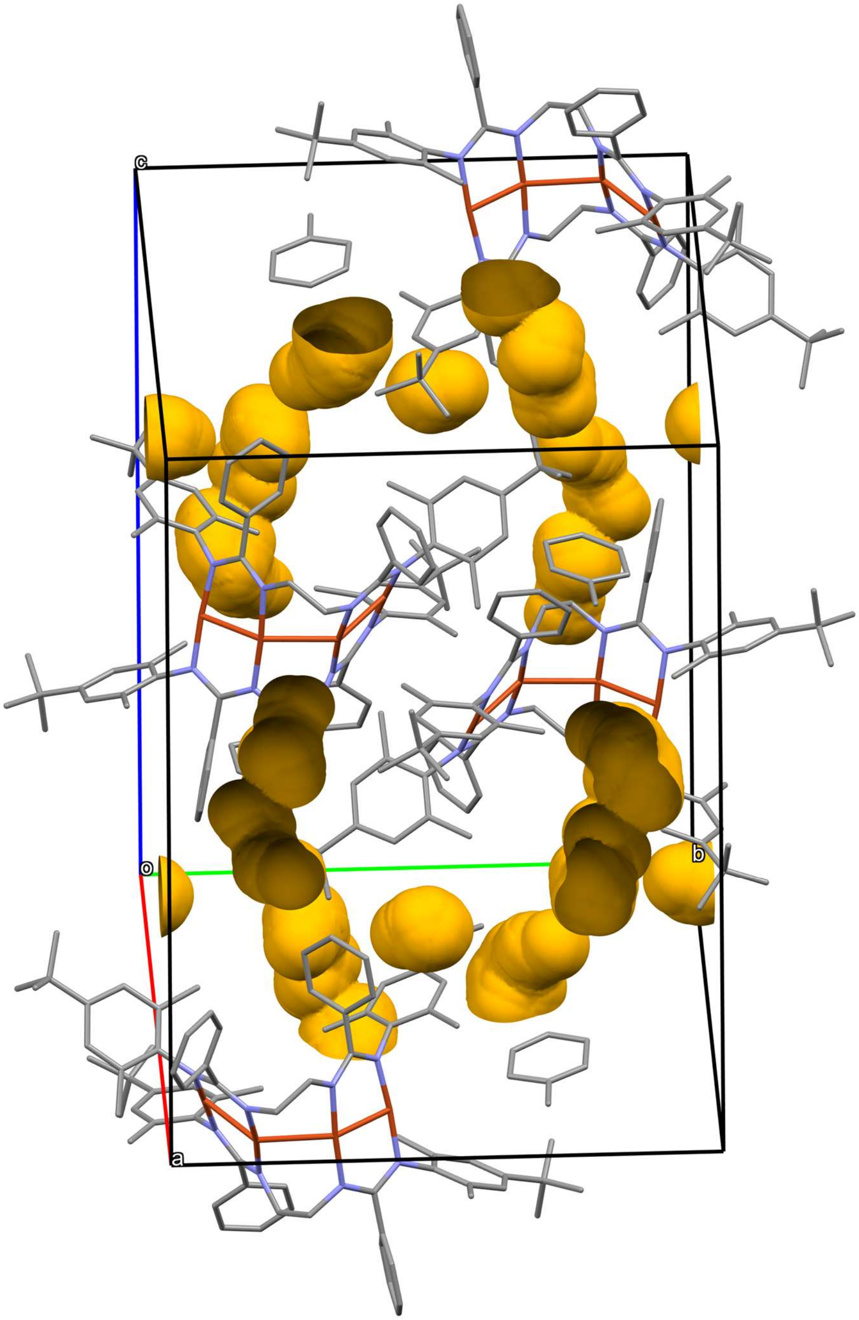

Figure S14: Crystal packing diagram and unit cell of **2**·C_7H_8 showing void spaces (spherical probe radius: 1.2 Å).[S3] Hydrogen atoms have been omitted for clarity.

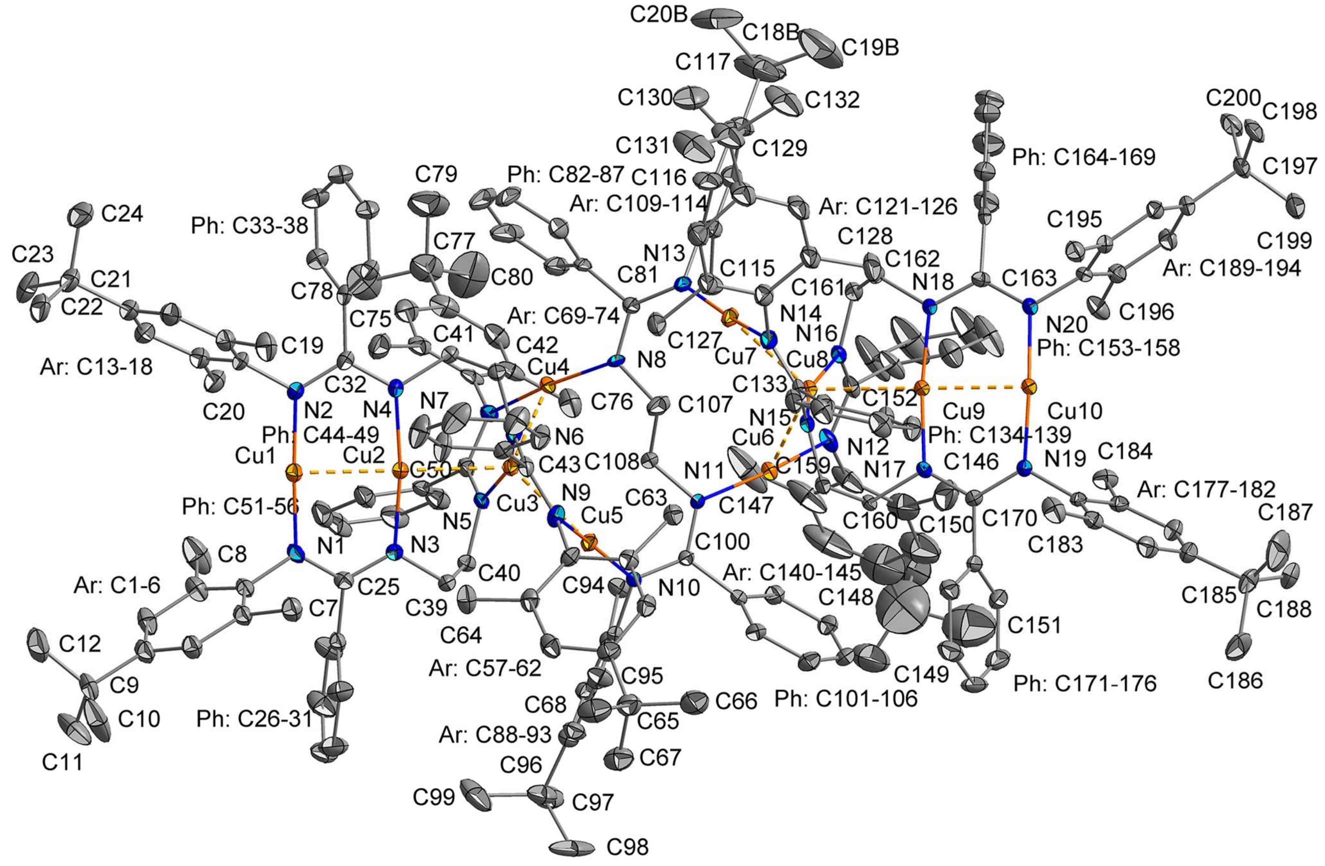

Figure S15: Molecular structure of **3** (*P* (Δ) enantiomer) showing ellipsoids with anisotropic displacement factors of 50% probability. Hydrogen atoms have been omitted for clarity. Selected interatomic distances (Å), bond angles (deg), and torsion angles (deg): Cu1···Cu2 2.4783(7), Cu2···Cu3 2.6077(7), Cu3···Cu4 2.6117(8), Cu3···Cu5 2.6664(8), Cu10···Cu9 2.4874(7), Cu9···Cu8 2.6437(7), Cu8···Cu6 2.6529(8), Cu8···Cu7 2.6559(8), Cu1–N1 1.869(4), Cu1–N2 1.871(3), Cu2–N3 1.907(3), Cu2–N4 1.909(3), Cu3–N5 1.887(3), Cu3–N6 1.885(3), Cu4–N7 1.870(3), Cu4–N8 1.888(4), Cu5–N9 1.884(3), Cu5–N10 1.877(3), Cu10–N20 1.874(3), Cu10–N19 1.878(3), Cu9–N18 1.897(3), Cu9–N17 1.897(3), Cu8–N16 1.888(4), Cu8–N15 1.888(4), Cu6–N12 1.871(4), Cu6–N11 1.883(3), Cu7–N14 1.882(4), Cu7–N13 1.878(4), C41–C42 1.519(6), N4–C41 1.465(5), C32–N4 1.332(5), N2–C32 1.336(5), C13–N2 1.446(5), N6–C42 1.471(5), C43–N6 1.320(5), N9–C43 1.328(5), C57–N9 1.436(5), C39–C40 1.523(6), N3–C39 1.469(5), C25–N3 1.326(5), N1–C25 1.342(5), C1–N1 1.440(5), N5–C40 1.465(5), C50–N5 1.326(5), N7–C50 1.341(5), C69–N7 1.432(5), C161–C162 1.522(7), N18–C162 1.470(5), C163–N18 1.325(5), N20–C163 1.340(5), C189–N20 1.438(5), N16–C161 1.472(5), C152–N16 1.315(6), N12–C152 1.340(6), C140–N12 1.426(6), C159–C160 1.526(6), N17–C160 1.469(5), C170–N17 1.330(5), N19–C170 1.339(5), C177–N19 1.440(5), N15–C159 1.460(5), C133–N15 1.334(6), N14–C133 1.337(6), C121–N14 1.428(6), Cu1–Cu2–Cu3 176.64(3), Cu2–Cu3–Cu4 119.74(3), Cu2–Cu3–Cu5

121.98(3), Cu4–Cu3–Cu5 118.20(2), Cu10–Cu9–Cu8 177.37(3), Cu9–Cu8–Cu6 118.87(3), Cu9–Cu8–Cu7 124.16(3), Cu6–Cu8–Cu7 116.81(2), N1–Cu1–Cu2 87.77(11), N2–Cu1–Cu2 88.99(11), N1–Cu1–N2 175.36(16), Cu1–N1–C25 122.2(3), Cu1–N2–C32 121.7(3), N3–Cu2–Cu1 87.30(10), N4–Cu2–Cu1 85.83(10), N3–Cu2–N4 172.73(14), Cu2–N4–C41 116.3(3), N4–C41–C42 111.8(3), C41–N4–C32 120.1(3), N4–C32–N2 119.9(4), C32–N2–C13 120.3(3), Cu2–N3–C39 118.6(3), N3–C39–C40 113.8(3), C39–N3–C25 119.8(3), N3–C25–N1 120.6(4), C25–N1–C1 119.4(4), N7–Cu4–Cu3 83.95(11), N8–Cu4–Cu3 100.01(12), N7–Cu4–N8 170.38(16), Cu4–N7–C50 122.6(3), Cu4–N7–C69 117.3(3), N6–Cu3–Cu4 105.25(11), N5–Cu3–Cu4 83.33(10), N6–Cu3–N5 161.52(14), Cu3–N5–C50 121.0(3), Cu3–N5–C40 117.6(3), C40–N5–C50 120.5(3), C50–N7–C69 119.9(3), N9–Cu5–Cu3 81.13(11), N10–Cu5–Cu3 112.67(10), N9–Cu5–N10 166.20(15), Cu5–N9–C43 125.8(3), Cu3–N5–C50 121.0(3), N5–Cu3–Cu5 106.84(10), N6–Cu3–Cu5 83.82(10), Cu3–N6–C43 120.7(3), Cu3–N6–C42 117.8(3), C42–N6–C43 121.2(3), N6–C43–N9 119.0(4), C43–N9–C57 124.8(4), N20–Cu10–Cu9 88.88(10), N19–Cu10–Cu9 87.55(10), N20–Cu10–N19 174.53(16), Cu10–N20–C163 120.6(3), Cu10–N19–C170 122.7(3), N18–Cu9–Cu10 86.18(10), N17–Cu9–Cu10 87.08(10), N18–Cu9–N17 173.08(14), Cu9–N17–C160 117.1(3), N17–C160–C159 112.2(3), C160–N17–C170 120.0(3), N17–C170–N19 120.0(4), C170–N19–C177 119.8(3), Cu9–N18–C162 117.4(3), N18–C162–C161 113.1(4), C162–N18–C163 119.9(3), N18–C163–N20 121.5(4), C163–N20–C189 117.9(3), N12–Cu6–Cu8 81.66(12), N11–Cu6–Cu8 110.39(11), N12–Cu6–N11 163.16(16), Cu6–N12–C152 124.9(3), Cu6–N12–C140 113.7(3), N16–Cu8–Cu6 84.07(11), N15–Cu8–Cu6 105.59(11), N16–Cu8–N15 160.24(14), Cu8–N15–C133 119.9(3), Cu8–N15–C159 117.5(3), C159–N15–C133 122.1(4), C133–N14–C121 124.4(4), N14–Cu7–Cu8 81.40(11), N13–Cu7–Cu8 110.58(12), N14–Cu7–N13 167.91(16), Cu7–N14–C133 124.5(3), Cu8–N16–C152, N16–Cu8–Cu6 84.07(11), N15–Cu8–Cu7 82.71(11), Cu8–N15–C133 119.9(3), Cu8–N15–C159 117.5(3), C159–N15–C133 122.1(4), N15–C133–N14 118.0(4), C133–N14–C121 124.4(4), Cu4–N8–C107 118.1(3), N8–C107–C108 110.2(4), C107–N8–C81 119.6(4), N8–C81–N13 , C81–N13–C109 115.6(4), Cu7–N13–C81 126.3(3), Cu6–N11–C108 112.7(3), N11–C108–C107 112.3(4), C108–N11–C100 119.6(3), N11–C100–N10 124.5(4), C100–N10–C88 117.6(3), Cu5–N10–C100 121.8(3), N1–Cu1–Cu2–N3 2.6(2),[S2] N1–Cu1–Cu2–N4 –179.7(2),[S2] N3–Cu2–Cu3–N6 –126.9(2),[S2] N4–Cu2–Cu3–N5 –123.4(2),[S2] Cu1–N1–C1–C2 –94.2(5),[S2] Cu1–N1–C1–C6 80.3(5),[S2] Cu1–N2–C13–C14 104.2(5),[S2] Cu1–N2–C13–C18 –69.7(5),[S2] N4–C41–C42–N6 62.3(4),[S2] C32–N4–C41–C42 120.7(4),[S2] N3–C39–C40–N5 60.3(5),[S2] C25–N3–C39–C40 109.1(4),[S2] N7–Cu4–Cu3–N5 21.8(2),[S2] N7–Cu4–Cu3–N6 –141.5(2),[S2] Cu4–N7–C69–C70 –105.0(4),[S2] Cu4–N7–C69–C74 72.5(5),[S2] N9–Cu5–Cu3–N6 21.5(2),[S2] N9–Cu5–Cu3–N5 –143.1(2),[S2] Cu5–N9–C57–C58 69.7(4),[S2] Cu5–N9–C57–C62 –98.7(4),[S2] N20–Cu10–Cu9–N18 –0.6(2),[S2] N20–Cu10–Cu9–N17 177.8(2),[S2] N18–Cu9–Cu8–N16 52.8(2),[S2] N17–Cu9–Cu8–N15 54.7(2),[S2] Cu10–N20–C189–C194 85.9(4),[S2] Cu10–N20–C189–C190 –93.4(4),[S2] Cu10–N19–C177–C178 106.0(4),[S2]

Cu10–N19–C177–C182 –68.1(4),[S2] N17–C160–C159–N15 60.7(4),[S2] C170–N17–C160–C159 120.0(4),[S2] N18–C162–C161–N16 60.8(5),[S2] C163–N18–C162–C161 107.2(5),[S2] N12–Cu6–Cu8–N16 21.3(2),[S2] N12–Cu6–Cu8–N15 –141.1(2),[S2] Cu6–N12–C140–C141 –108.4(5),[S2] Cu6–N12–C140–C145 63.3(6),[S2] N14–Cu7–Cu8–N15 25.6(2),[S2] N14–Cu7–Cu8–N16 –137.7(2),[S2] Cu7–N14–C121–C122 66.0(5),[S2] Cu7–N14–C121–C126 –103.8(5),[S2] Cu4–N8–C107–C108 19.3(5),[S2] N8–C107–C108–N11 167.7(4),[S2] C107–N8–C81–N13 –4.1(7),[S2] N8–C81–N13–C109 151.1(5),[S2] C81–N13–C109–C110 –61.5(6),[S2] C81–N13–C109–C114 123.4(5),[S2] Cu7–N13–C81–N8 –29.7(7),[S2] Cu6–N11–C108–C107 –62.6(4),[S2] C108–N11–C100–N10 –16.5(6),[S2] N11–C100–N10–C88 142.9(4),[S2] C100–N10–C88–C89 –59.7(5),[S2] C100–N10–C88–C93 127.2(4),[S2] Cu5–N10–C100–N11 –42.3(5)[S2]. Symmetry operation used to generate equivalent atoms in the crystal packing (*P* (Δ) enantiomer): $-x+1, -y+1, -z$.

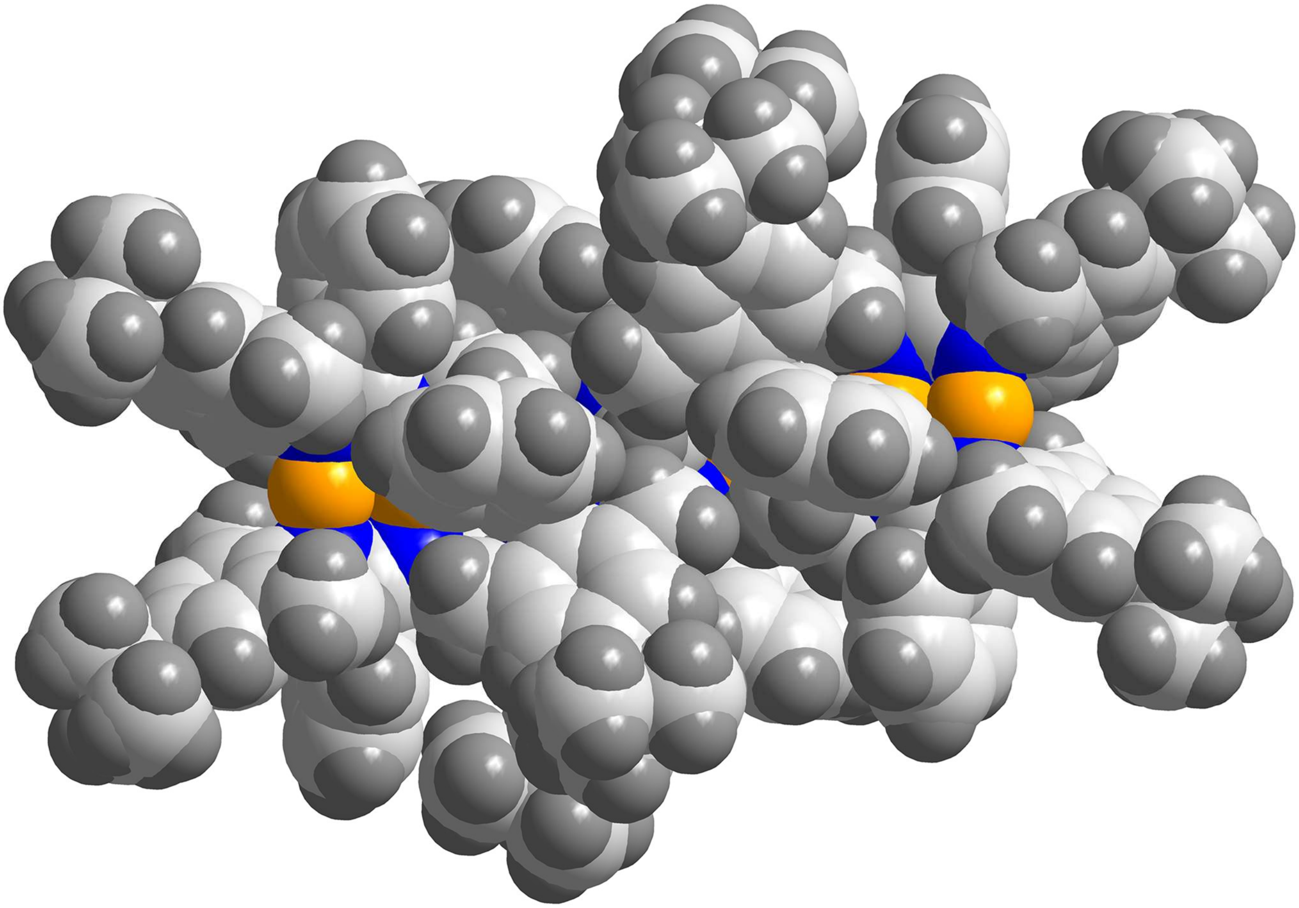

Figure S16: Space filling representation of the molecular structure of **3** (P (Δ) enantiomer).

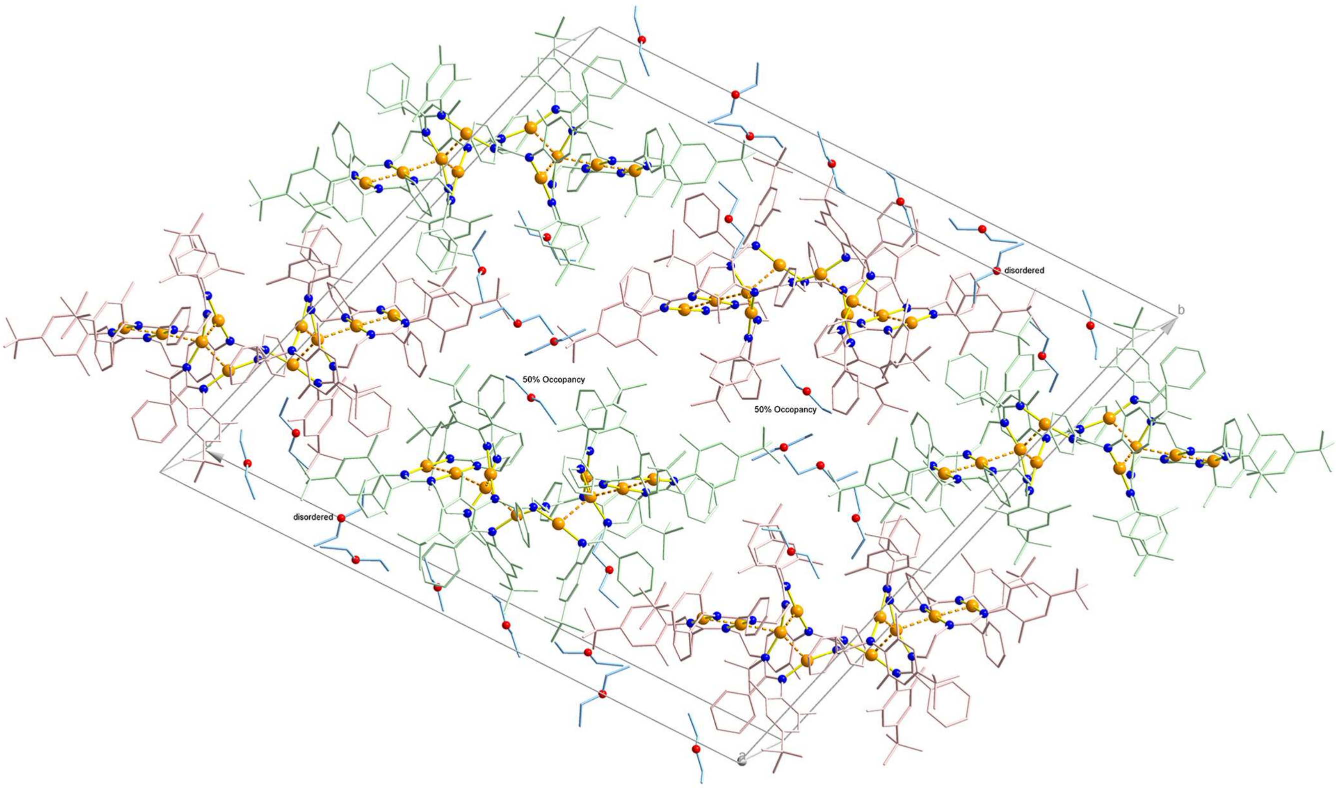

Figure S17: Crystal packing diagram and unit cell of **3**·$5Et_2O$ showing a racemate consisting of *P*(Δ, green)- and *M*(Λ, salmon)-helices. Hydrogen atoms have been omitted for clarity. Four equivalent diethyl ether molecules are disordered and only their dominant conformation is depicted. Two additional diethyl ether molecules in the unit cell are half occupied.

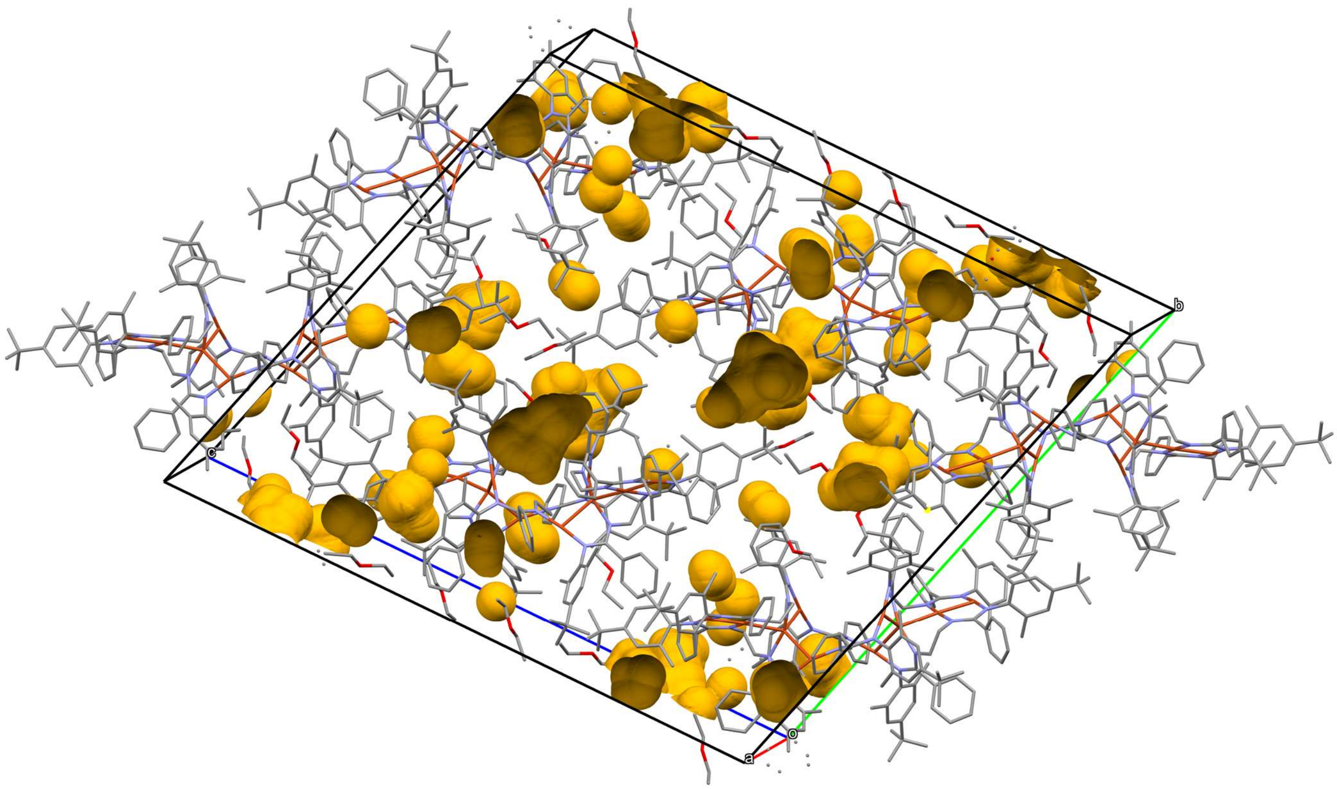

Figure S18: Crystal packing diagram and unit cell of **3**·5Et_2O showing void spaces (spherical probe radius: 1.2 Å).[S3] Hydrogen atoms have been omitted for clarity.

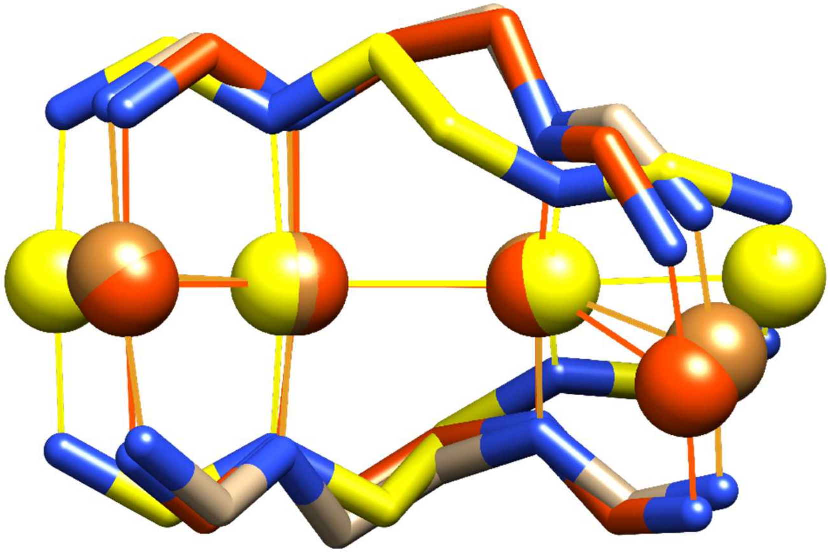

Figure S19: Overlay representation of the molecular XRD structures of **I** (brown),[S4] **1** (yellow), and **2** (red).[S5] Aryl substituents and hydrogen atoms have been omitted for clarity.

Table S1. Crystal data and refinement details for $\mathbf{L^1}H_2$ and $\mathbf{L^2}H_2$.

	$\mathbf{L^1}H_2$	$\mathbf{L^2}H_2$
Empirical formula	$C_{40}H_{50}N_4$	$C_{40}H_{50}N_4$
M_r	586.84	586.84
Crystal size [mm]	0.291× 0.061 × 0.036	0.173 × 0.11 × 0.102
Crystal system	triclinic	trigonal
Space group	$P\bar{1}$	$R\bar{3}$
a [Å], α [°]	13.4802(3), 70.3060(10)	25.3118(7), 90
b [Å], β [°]	16.4348(4), 84.2230(10)	25.3118(7), 90
c [Å], γ [°]	16.6146(4), 85.3130(10)	14.4924(4), 120
V [Å^3]	3443.47(14)	8041.1(5)
Z	4	9
$\rho_{calcd.}$ [g cm^{-3}]	1.132	1.091
$F(000)$	1272	2862
μ [mm^{-1}]	0.502	0.484
T_{max}/T_{min}	0.5835 / 0.5160	0.4684 / 0.3867
hkl range	–15 +14, ±19, ±19	±30, –30 +27, ±17
θ range [°]	2.833 – 65.527	3.49 – 70.20
Measured refl.	41386	13487
Unique refl. [R_{int}]	11722 [0.0471]	3379 [0.1187]
Data / restr. / param.	11722 / 0 / 809	3379 / 0 / 205
Goodness-of-fit	1.060	1.046
$R1$ ($I>2\sigma(I)$)	0.0508	0.0521
$wR2$ (all data)	0.1149	0.1379
Resid. electron dens. [e Å^{-3}]	0.279 / –0.202	0.285 / –0.385

Table S2. Crystal data and refinement details for **1**·1.5C_7H_8, **2**·C_7H_8, and **3**·5Et_2O.

	**1**·1.5C_7H_8	**2**·C_7H_8	**3**·5Et_2O
Empirical formula	$C_{80}H_{96}N_8Cu_4 \cdot 1.5C_7H_8$	$C_{80}H_{96}N_8Cu_4 \cdot C_7H_8$	$C_{200}H_{240}N_{20}Cu_{10} \cdot 5C_4H_{10}O$
M_r	1562.00	1515.94	3930.10
Crystal size [mm]	0.102 × 0.116 × 0.181	0.025 × 0.106 × 0.261	0.038 × 0.092 × 0.172
Crystal system	monoclinic	monoclinic	triclinic
Space group	*C*2/*c*	*P*2₁/*n*	$P\bar{1}$
a [Å], *α* [°]	37.269(3), 90	18.0873(11), 90	16.417(2), 106.510(5)
b [Å], *β* [°]	12.5811(12), 110.617(3)	18.2870(11), 102.700(2)	34.771(5), 91.083(5)
c [Å], *γ* [°]	19.2360(17), 90	24.8053(15), 90	39.151(6), 96.396(5)
V [Å^3]	8441.8(13)	8003.9(8)	21264.(5)
Z	4	4	4
$\rho_{calcd.}$ [g cm^{-3}]	1.229	1.258	1.228
F(000)	3292	3192	8320
μ [mm^{-1}]	1.042	1.097	1.036
T_{max}/T_{min}	1.0000 / 0.9323	1.0000 / 0.7982	0.9620 / 0.8420
hkl range	–47 +44, 0 +16, 0 +24	±19, ±19, –26 +24	±19, ±42, ±47
θ range [°]	2.385 – 27.128	2.502 – 22.012	2.051 – 25.500
Measured refl.	9294	42319	764415
Unique refl. [R_{int}]	9294 [0.0524]	9791 [0.0786]	79089 [0.0597]
Data / restr. / param.	9294 / 156 / 553	9791 / 58 / 913	79089 / 545 / 4896
Goodness-of-fit	1.124	1.020	1.210
$R1$ (I>2σ(I))	0.0340	0.0483	0.0630
$wR2$ (all data)	0.1039	0.1266	0.1322
Resid. electron dens. [e Å^{-3}]	1.271 / –0.408	1.038 / –0.606	1.427 / –0.710

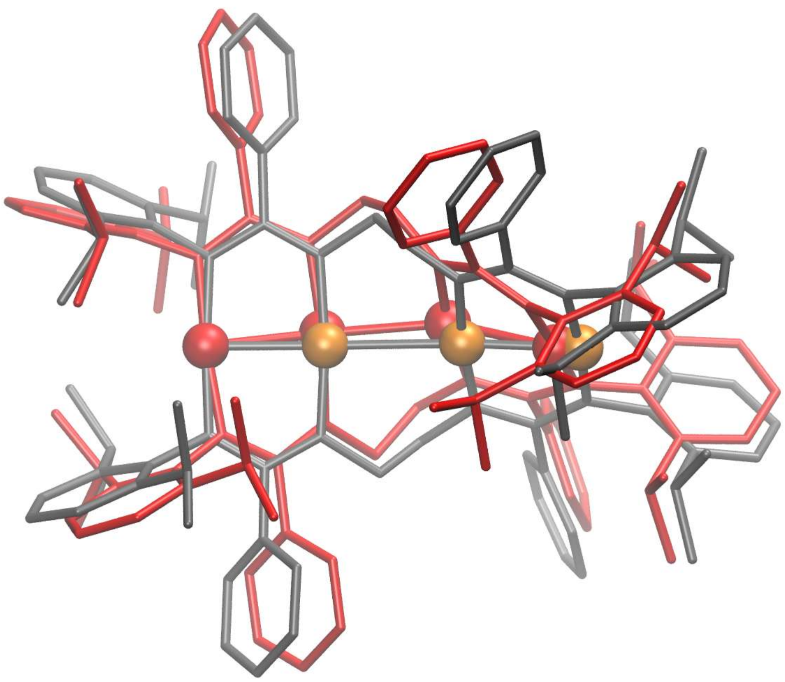

Figure S20: Overlay representation of the molecular XRD (gray/orange) and geometry-optimized computational structures (red) of **1** (PBE1PBE).[S6]
Hydrogen atoms have been omitted for clarity.

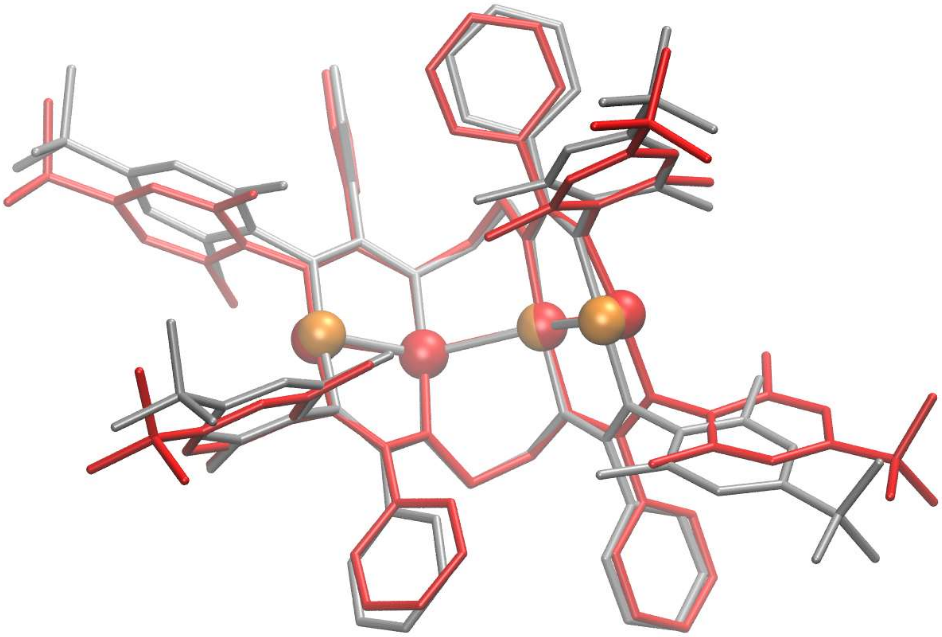

Figure S21: Overlay representation of the molecular XRD (gray/orange) and geometry-optimized computational structures (red) of **2** (PBE1PBE).[S6]
Hydrogen atoms have been omitted for clarity.

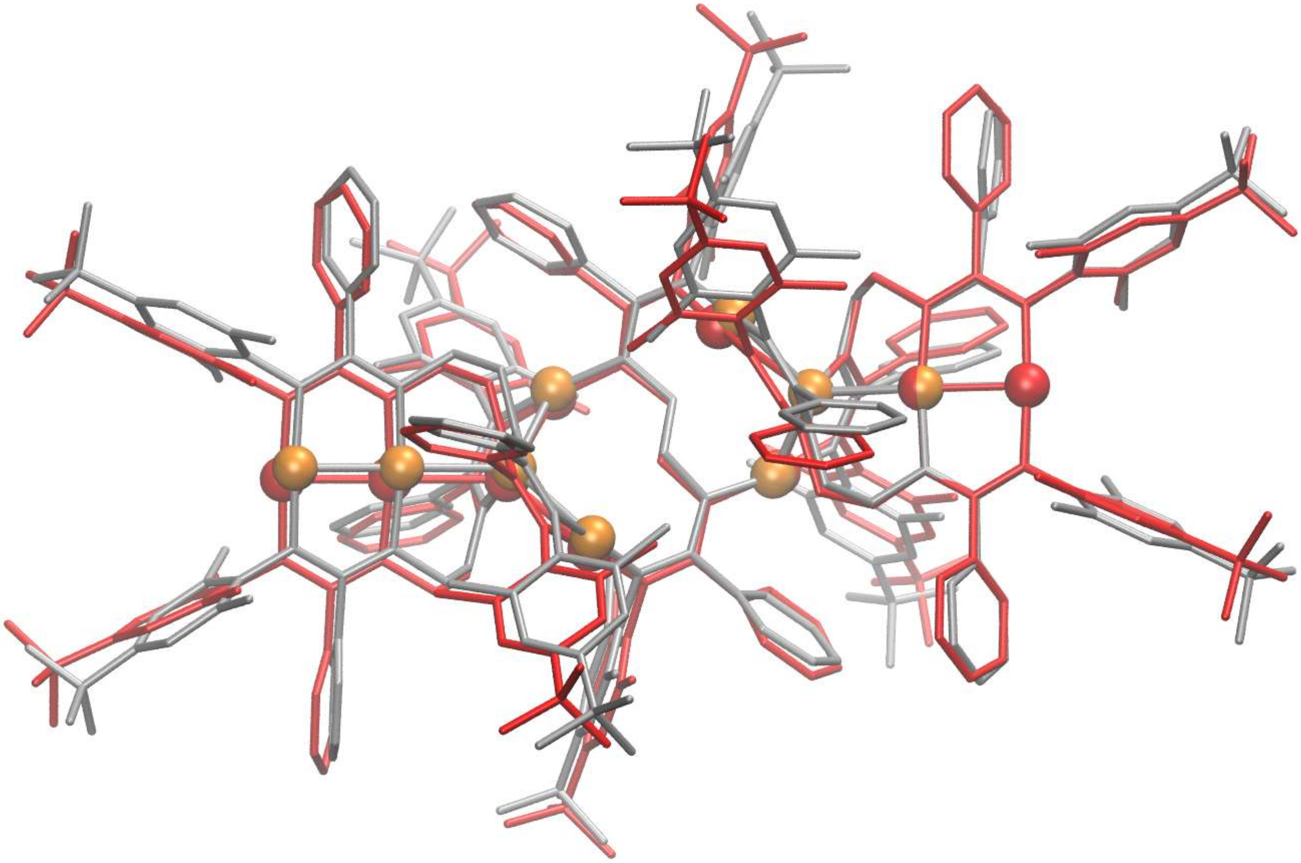

Figure S22: Overlay representation of the molecular XRD (gray/orange) and geometry-optimized computational structures (red) of **3** (PBE1PBE).[S6]
Hydrogen atoms have been omitted for clarity.

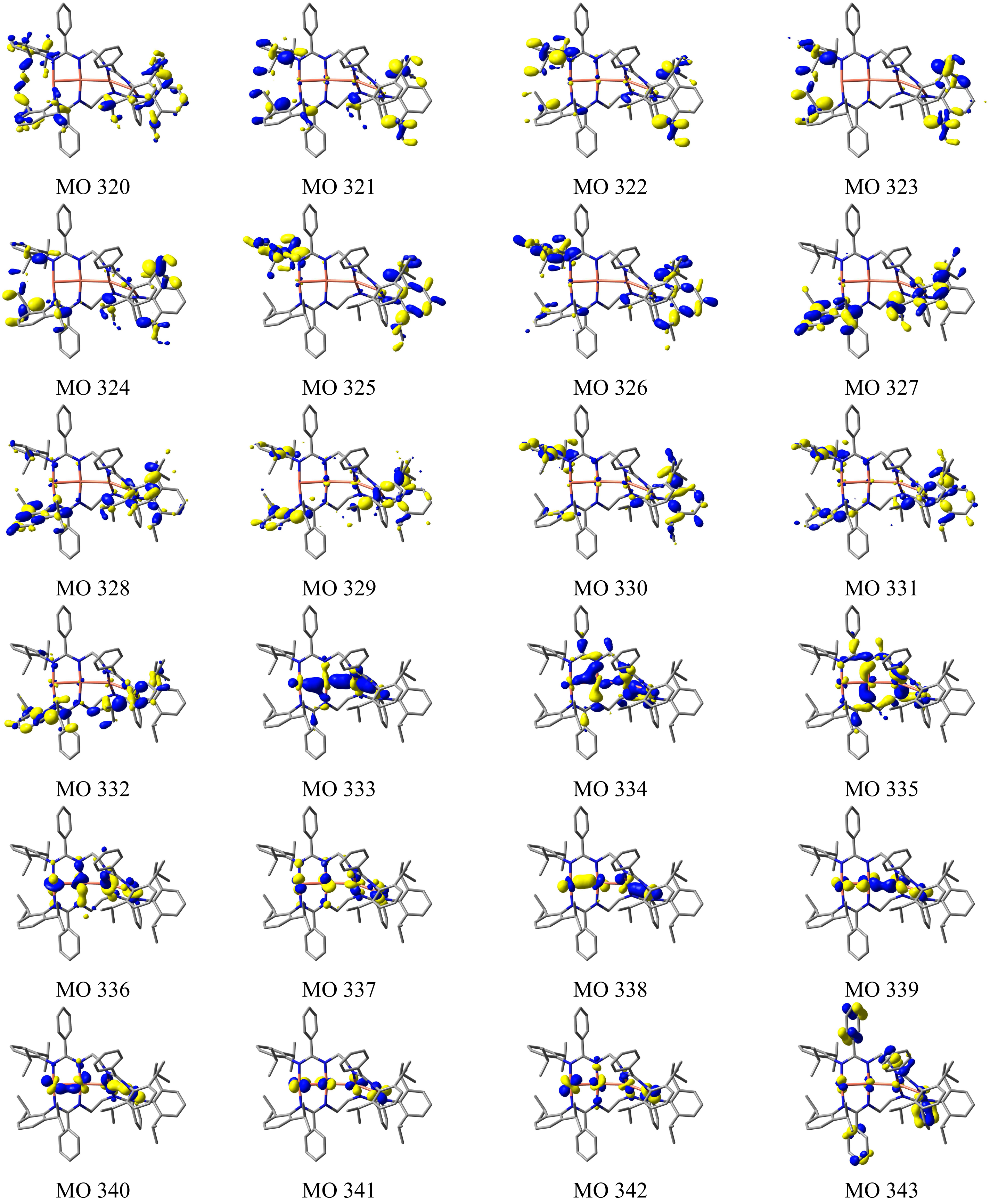

Figure S23: Isodensity plots of orbitals 320 (HOMO-54) through 343 (HOMO-31) for **1** (isovalue = 0.04 a.u.).

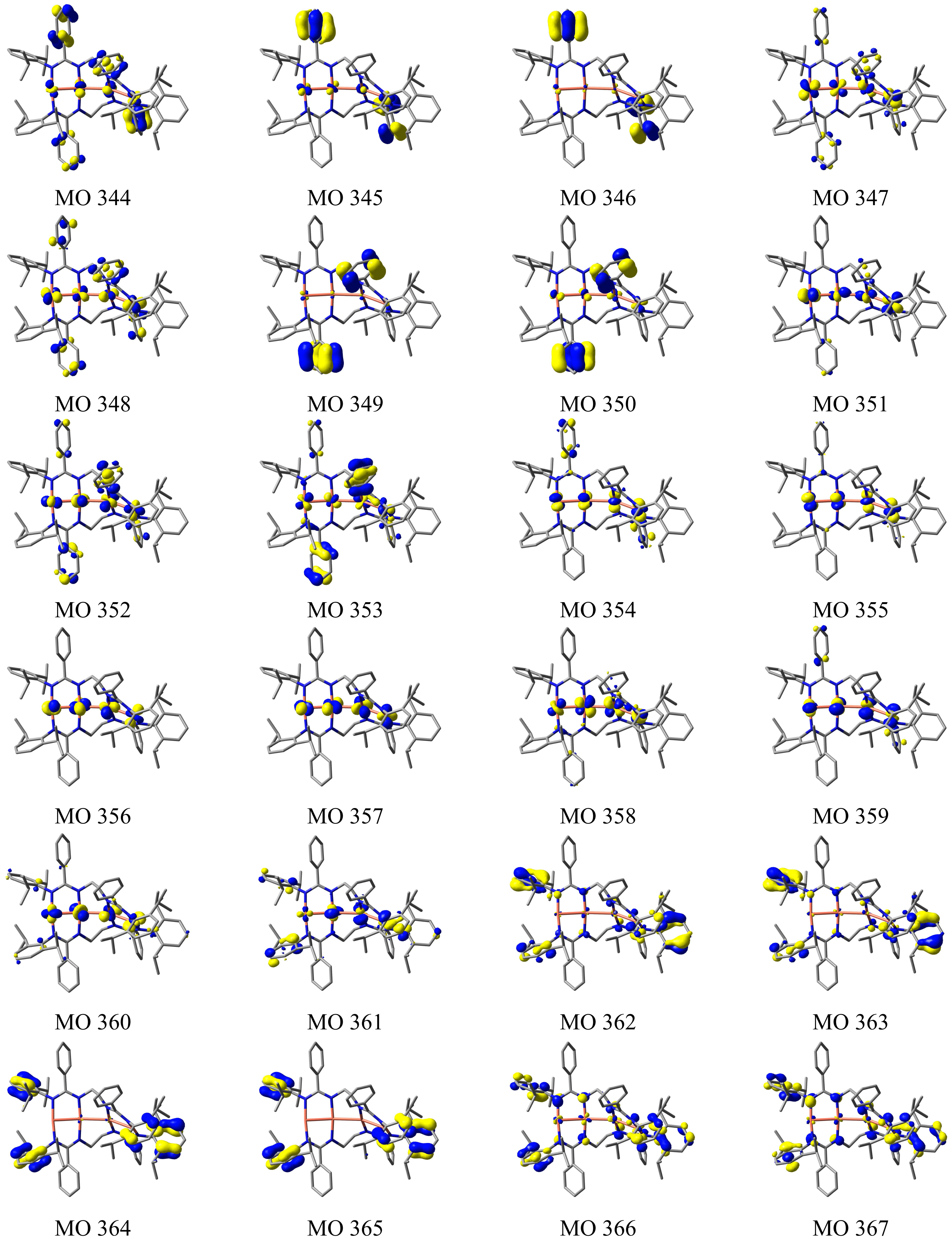

Figure S24: Isodensity plots of orbitals 344 (HOMO-30) through 367 (HOMO-7) for **1** (isovalue = 0.04 a.u.).

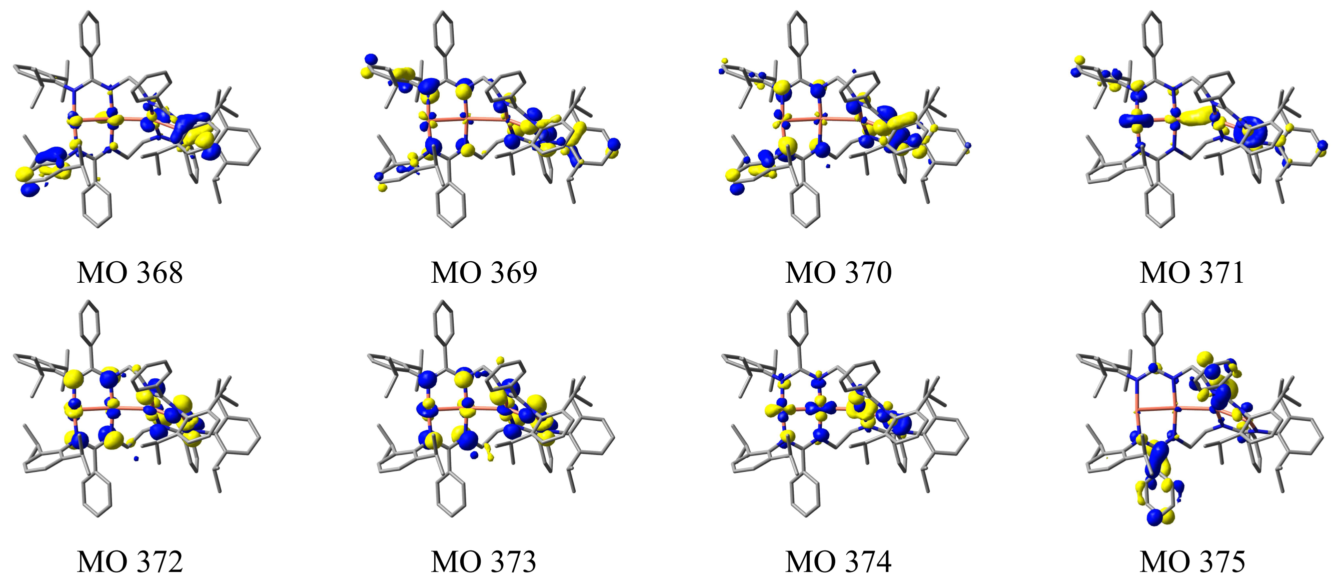

Figure S25: Isodensity plots of orbitals 368 (HOMO-6) through 375 (LUMO) for **1** (isovalue = 0.04 a.u.).

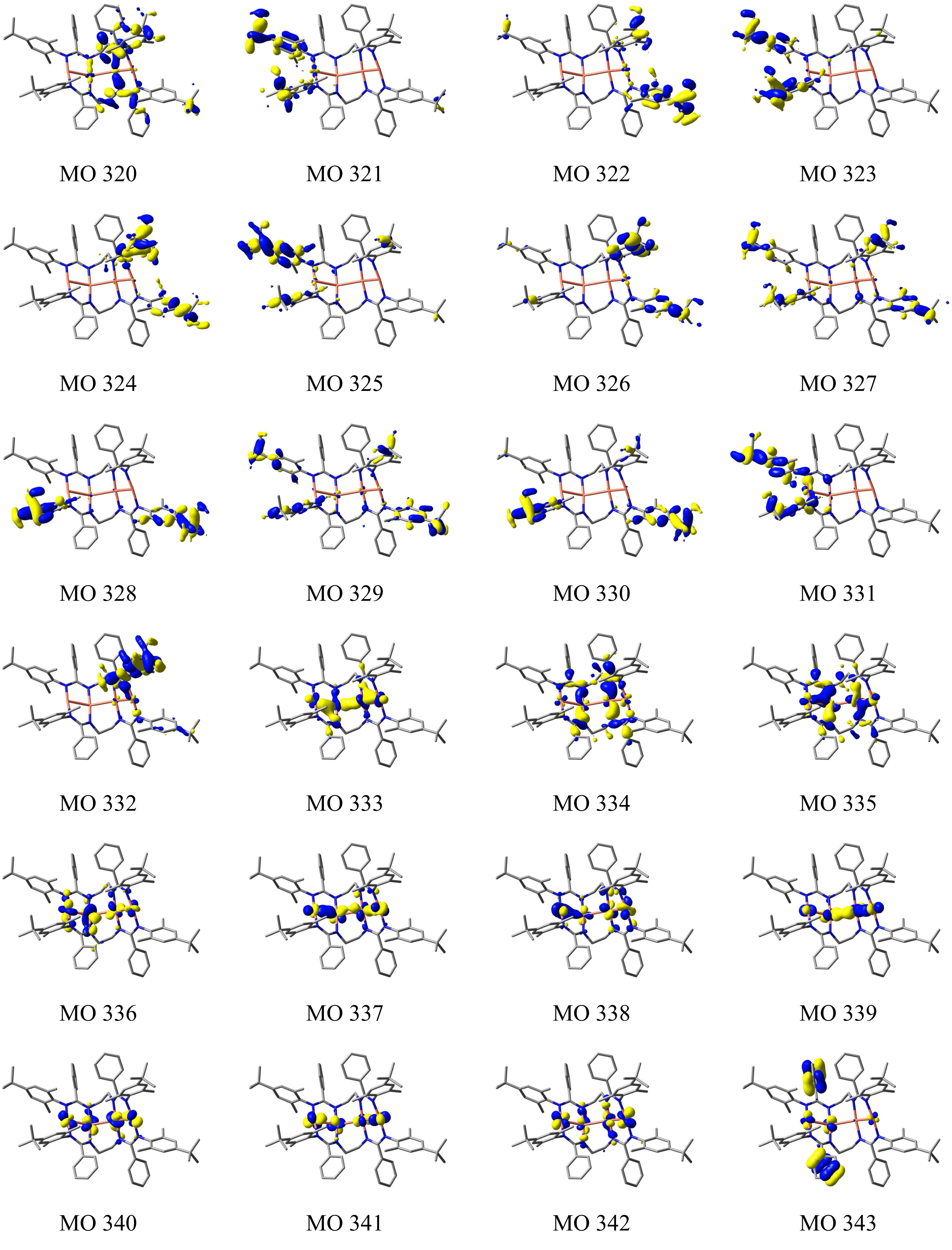

Figure S26: Isodensity plots of orbitals 320 (HOMO-54) through 343 (HOMO-31) for **2** (isovalue = 0.04 a.u.).

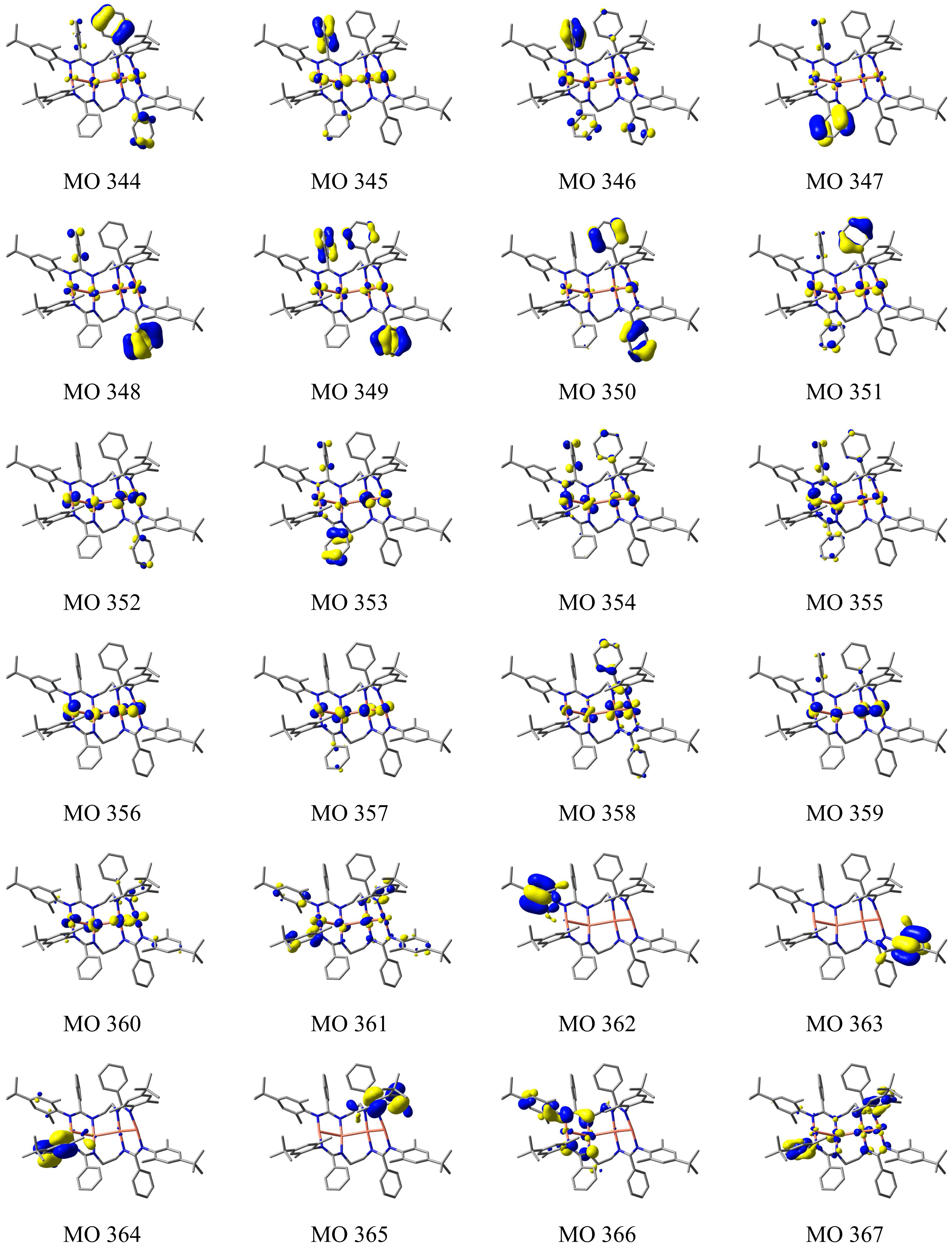

Figure S27: Isodensity plots of orbitals 344 (HOMO-30) through 367 (HOMO-7) for **2** (isovalue = 0.04 a.u.).

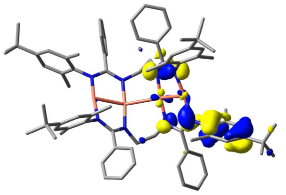

MO 368

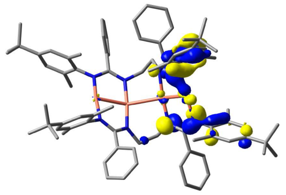

MO 369

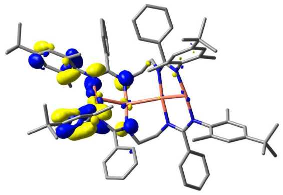

MO 370

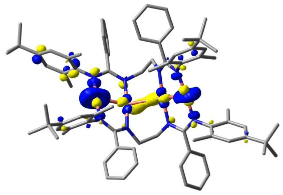

MO 371

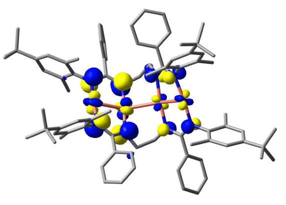

MO 372

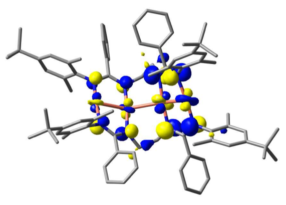

MO 373

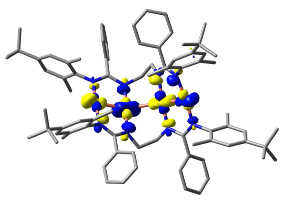

MO 374

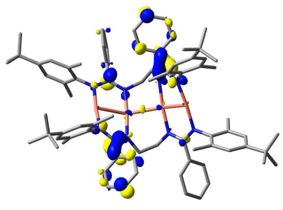

MO 375

Figure S28: Isodensity plots of orbitals 368 (HOMO-6) through 375 (LUMO) for **2** (isovalue = 0.04 a.u.).

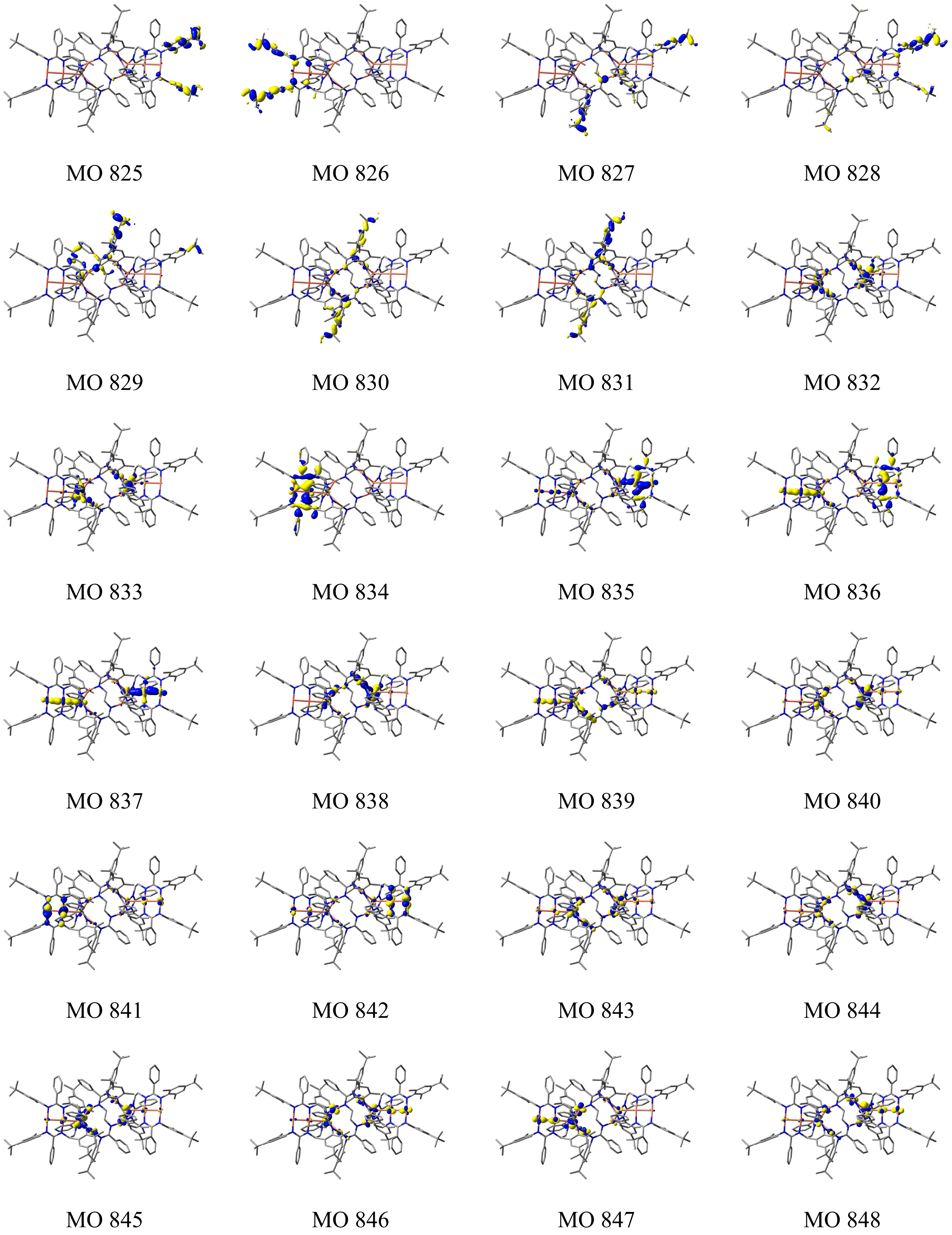

Figure S29: Isodensity plots of orbitals 825 (HOMO-110) through 848 (HOMO-87) for **3** (isovalue = 0.04 a.u.).

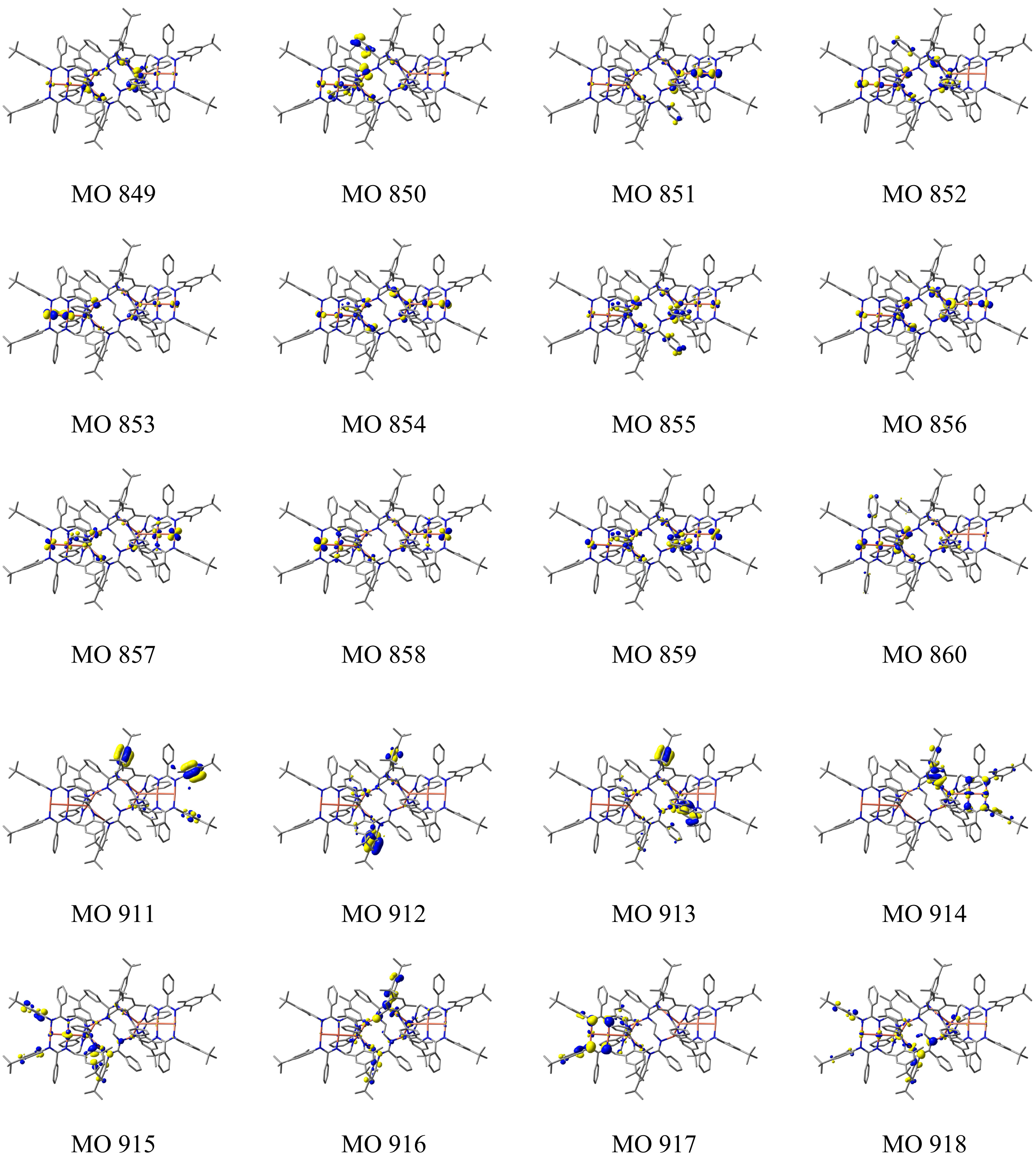

Figure S30: Isodensity plots of orbitals 849 (HOMO-86) through 860 (HOMO-75) and 911 (HOMO-24) through 918 (HOMO-17) for **3** (isovalue = 0.04 a.u.).

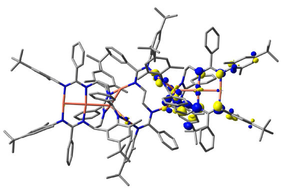

MO 919

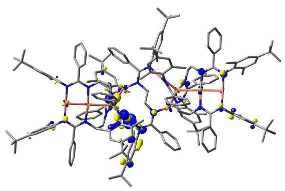

MO 920

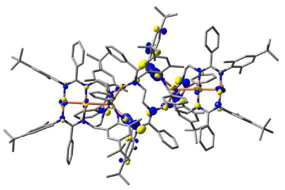

MO 931

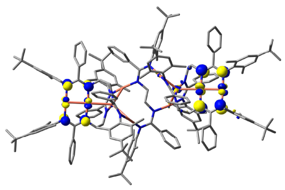

MO 932

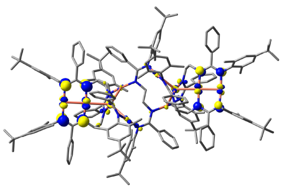

MO 933

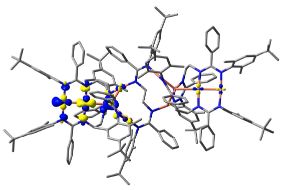

MO 934

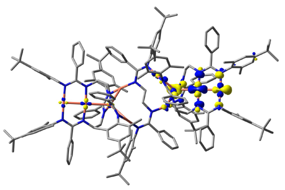

MO 935

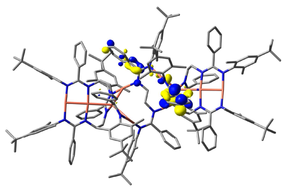

MO 936

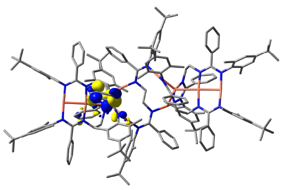

MO 937

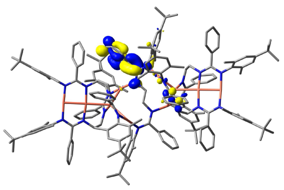

MO 938

Figure S31: Isodensity plots of orbitals 919 (HOMO-16), 920 (HOMO-15), and 931(HOMO-4) through 938 (LUMO+2) for **3** (isovalue = 0.04 a.u.).

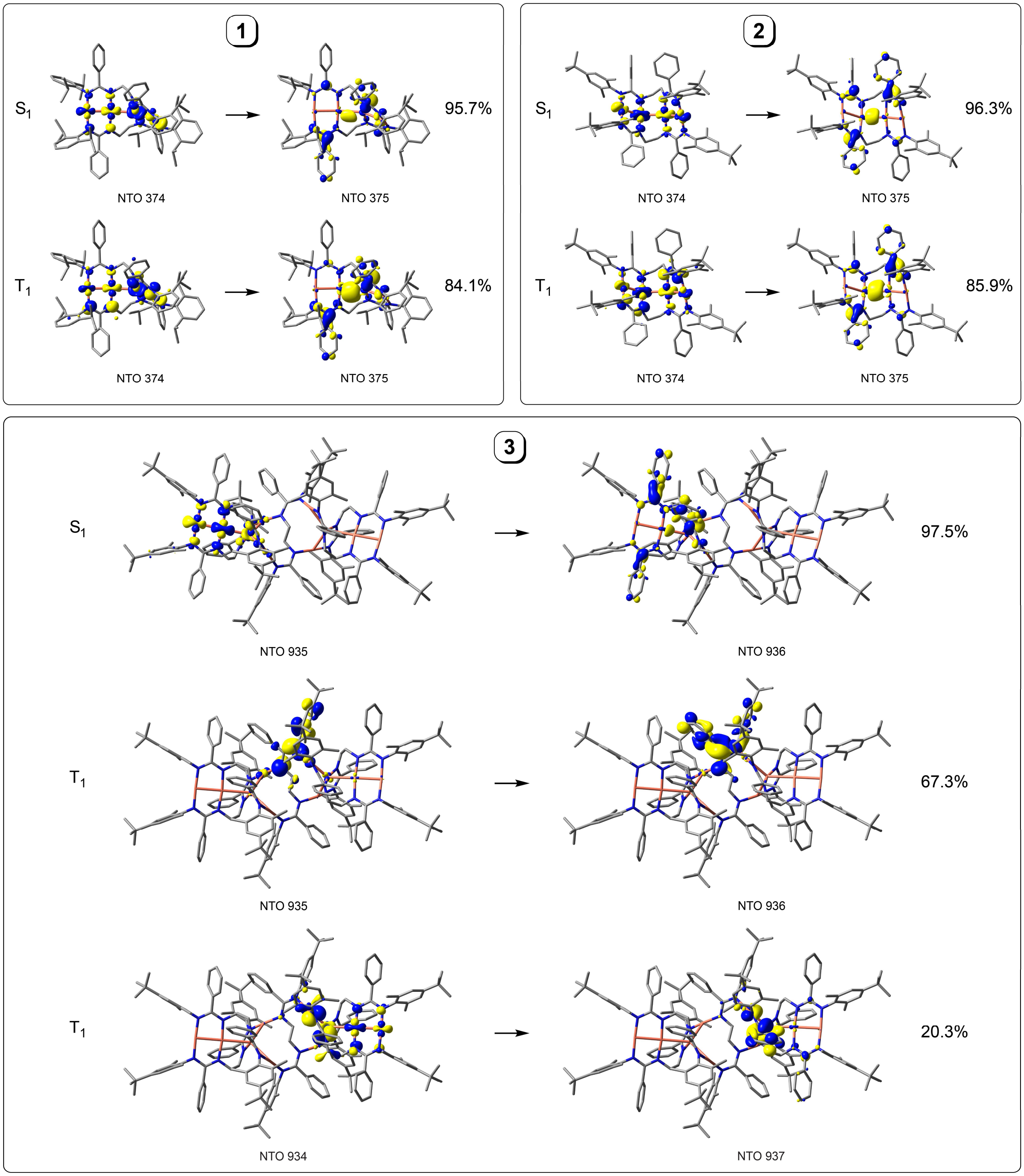

Figure S32: NTO pairs for the ground-state optimized structures of **1−3** with the highest weight in describing the S_1 and T_1 excitations.

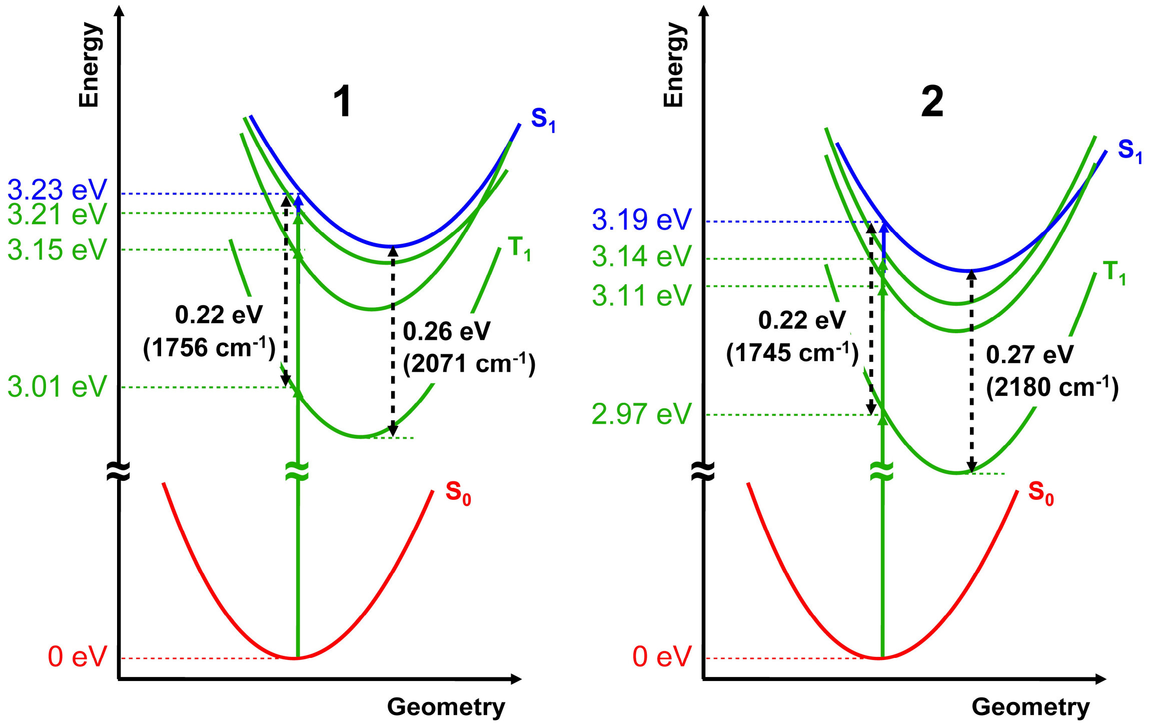

Figure S33: Schematic representation of the electronic states of **1** and **2** as harmonic potentials. The vertical excitation energies are labeled in the corresponding colors. The vertical (left) and adiabatic (right) excitation energies between the T_1 and S_1 states are labeled in black.

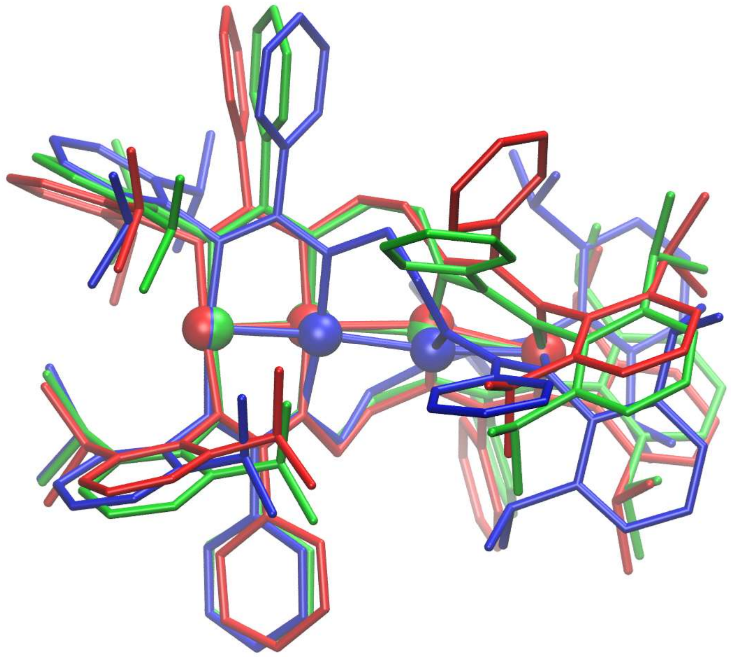

Figure S34: Overlay representation of the geometry-optimized computational structures of **1** in the ground state (S_0, red), first singlet excited state (S_1, blue), and first triplet excited state (T_1, green).[S6] Hydrogen atoms have been omitted for clarity.

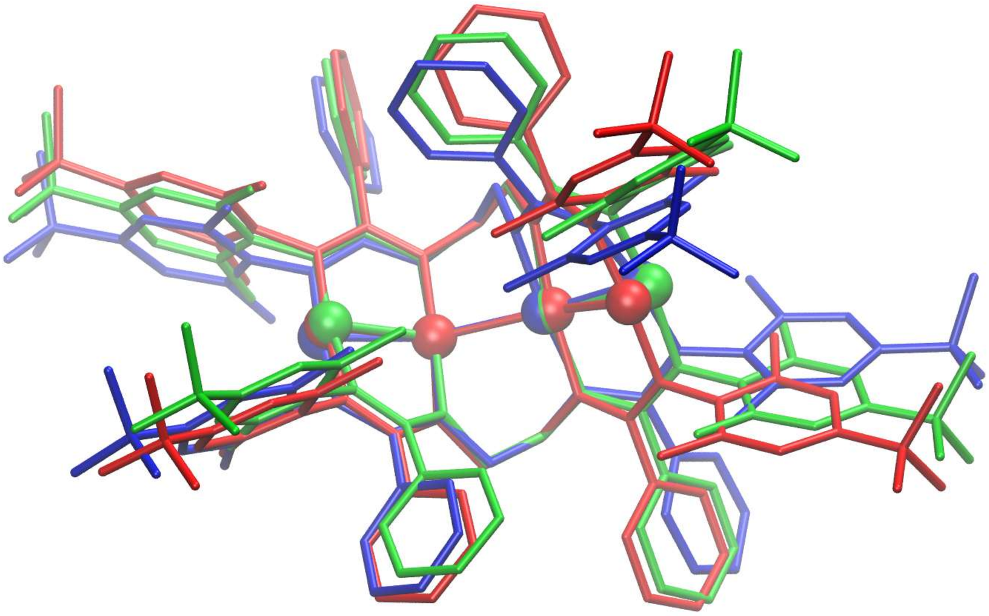

Figure S35: Overlay representation of the geometry-optimized computational structures of **2** in the ground state (S_0, red), first singlet excited state (S_1, blue), and first triplet excited state (T_1, green).[S6] Hydrogen atoms have been omitted for clarity.

Table S3. Overview of selected calculated and measured distances (Å) and angles (deg) for **1**.

	XRD	S_0	S_1	T_1
Cu1···Cu2'	2.4771(4)	2.475	2.369	2.482
Cu1'···Cu2	2.4771(4)	2.475	2.433	2.464
Cu2···Cu2'	2.8702(6)	2.645	2.446	2.561
Cu1–N2	1.8648(19)	1.891	1.890	1.893
Cu1'–N2'	1.8648(19)	1.892	1.883	1.892
Cu1–N4'	1.870(2)	1.891	1.858	1.878
Cu1'–N4	1.870(2)	1.891	1.884	1.892
Cu2'–N1	1.888(2)	1.908	1.886	1.897
Cu2–N1'	1.888(2)	1.908	1.890	1.905
Cu2'–N3'	1.894(2)	1.909	1.846	1.871
Cu2–N3	1.894(2)	1.909	1.891	1.903
N2–C3	1.336(3)	1.336	1.330	1.336
N2'–C3'	1.336(3)	1.336	1.338	1.334
N1–C3	1.333(3)	1.329	1.332	1.331
N1'–C3'	1.333(3)	1.330	1.328	1.329
N4'–C22'	1.333(3)	1.335	1.378	1.388
N4–C22	1.333(3)	1.336	1.333	1.334
N3'–C22'	1.330(3)	1.330	1.382	1.415
N3–C22	1.330(3)	1.330	1.334	1.333
Cu1–Cu2'–Cu2	176.400(15)	159.4	173.7	169.1
Cu1'–Cu2–Cu2'	176.400(15)	159.3	171.9	149.4
N2–Cu1–Cu2'	87.94(6)	86.9	87.4	86.0
N2'–Cu1'–Cu2	87.94(6)	86.9	86.8	86.8
N2–Cu1–Cu2'–N3'	–172.0(1)[S2]	–163.6	–160.7	–158.6
N2'–Cu1'–Cu2–N3	–172.0(1)[S2]	–163.6	–159.3	–168.8
N4'–Cu1–Cu2'–N1	–171.4(1)[S2]	–168.5	–157.0	–166.5
N4–Cu1'–Cu2–N1'	–171.4(1)[S2]	–168.5	–161.2	–168.8
N1–Cu2'–Cu2–N1'	–136.2(1)[S2]	–135.0	–123.1	–134.6
N3'–Cu2'–Cu2–N3	–135.9(1)[S2]	–137.1	–126.7	–141.4

Table S4. Overview of selected calculated and measured distances (Å) and angles (deg) for **2**.

	**XRD**	**S_0**	**S_1**	**T_1**
Cu3···Cu1	2.4398(9)	2.460	2.430	2.444
Cu4···Cu2	2.4579(9)	2.467	2.353	2.493
Cu1···Cu2	2.6464(9)	2.664	2.488	2.593
Cu3–N5	1.870(4)	1.895	1.879	1.896
Cu4–N8	1.884(4)	1.892	1.883	1.886
Cu3–N6	1.858(4)	1.894	1.882	1.897
Cu4–N7	1.879(4)	1.894	1.855	1.871
Cu1–N1	1.867(4)	1.896	1.896	1.887
Cu2–N4	1.880(4)	1.905	1.890	1.904
Cu1–N2	1.875(4)	1.899	1.901	1.890
Cu2–N3	1.879(4)	1.906	1.849	1.871
N5–C5	1.326(6)	1.335	1.336	1.335
N8–C8	1.327(6)	1.335	1.334	1.333
N1–C5	1.344(6)	1.330	1.329	1.332
N4–C8	1.326(6)	1.327	1.330	1.331
N6–C6	1.341(6)	1.336	1.331	1.333
N7–C7	1.337(7)	1.336	1.379	1.387
N2–C6	1.324(6)	1.330	1.336	1.334
N3–C7	1.326(6)	1.331	1.383	1.414
Cu3–Cu1–Cu2	131.37(3)	136.0	149.1	133.6
Cu4–Cu2–Cu1	137.41(4)	148.0	169.4	168.9
N5–Cu3–Cu1	88.30(13)	87.7	88.1	86.8
N8–Cu4–Cu2	88.18(13)	87.6	87.6	87.8
N5–Cu3–Cu1–N2	–170.5(2)[S2]	–169.8	–165.6	–168.4
N8–Cu4–Cu2–N3	–168.9(2)[S2]	–166.9	–161.7	–166.3
N6–Cu3–Cu1–N1	–172.3(2)[S2]	–170.2	–170.9	–165.3
N7–Cu4–Cu2–N4	–177.5(2)[S2]	–169.9	–158.5	–168.1
N1–Cu1–Cu2–N4	–151.8(2)[S2]	–148.6	–130.3	–142.0
N2–Cu1–Cu2–N3	–151.7(2)[S2]	–146.6	–136.5	–145.7

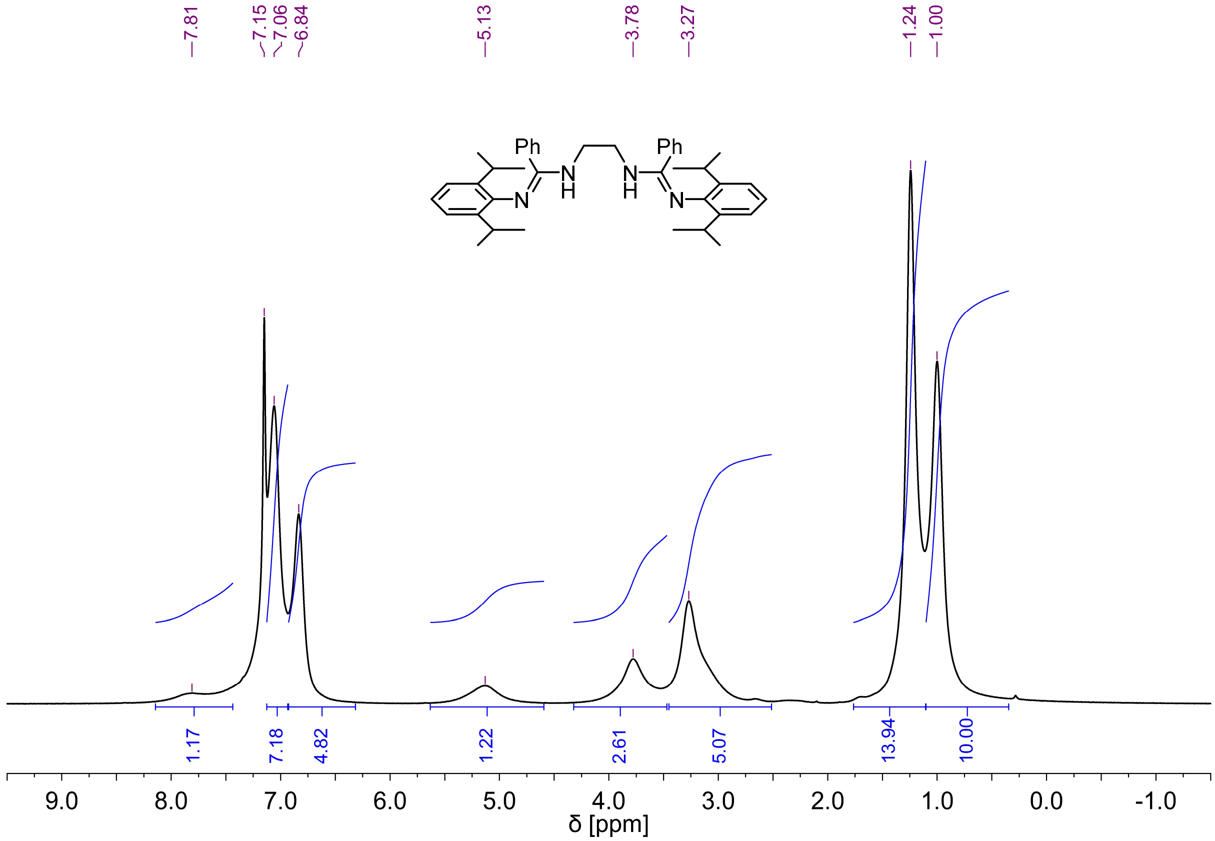

Figure S36: ^{1}H NMR spectrum of $\mathbf{L^1}H_2$ (C_6D_6, 400.1 MHz).

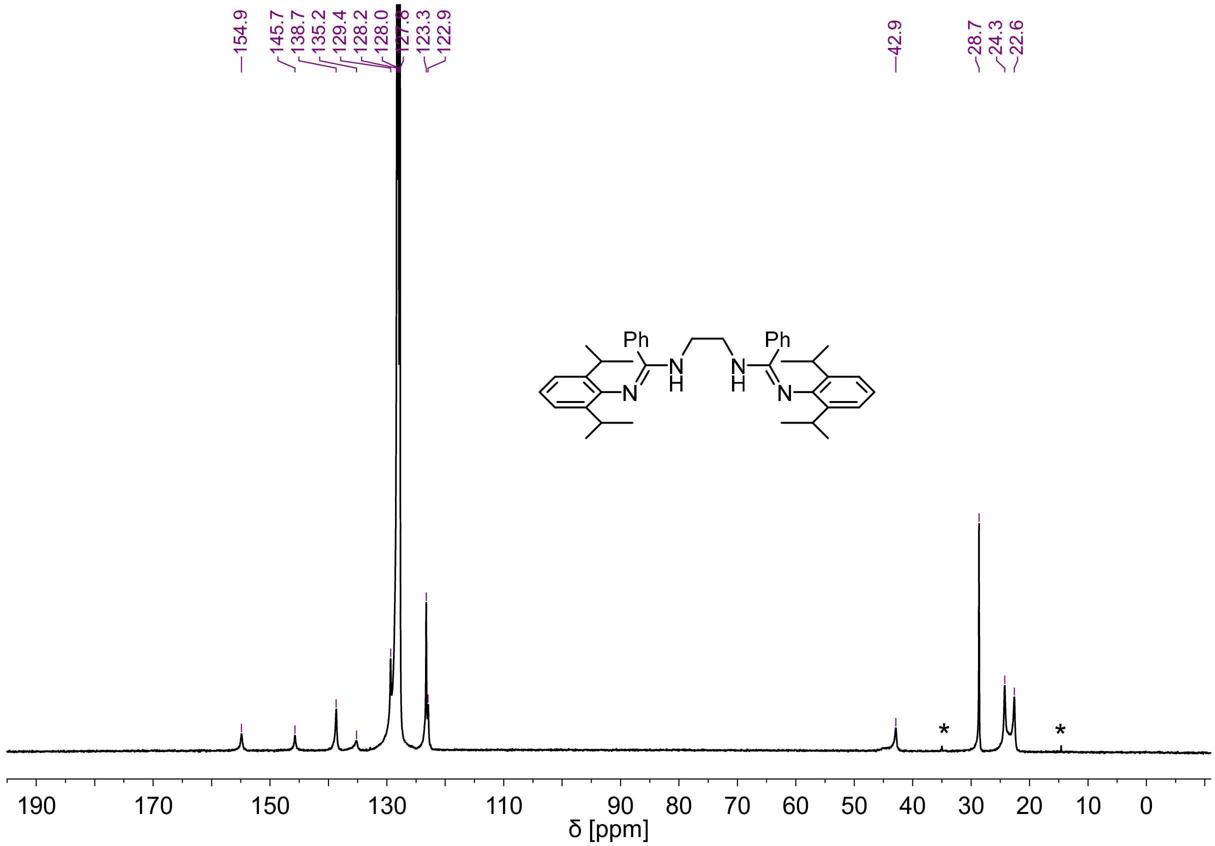

Figure S37: ^{13}C NMR spectrum of $\mathbf{L^1}H_2$ (C_6D_6, 100.6 MHz)

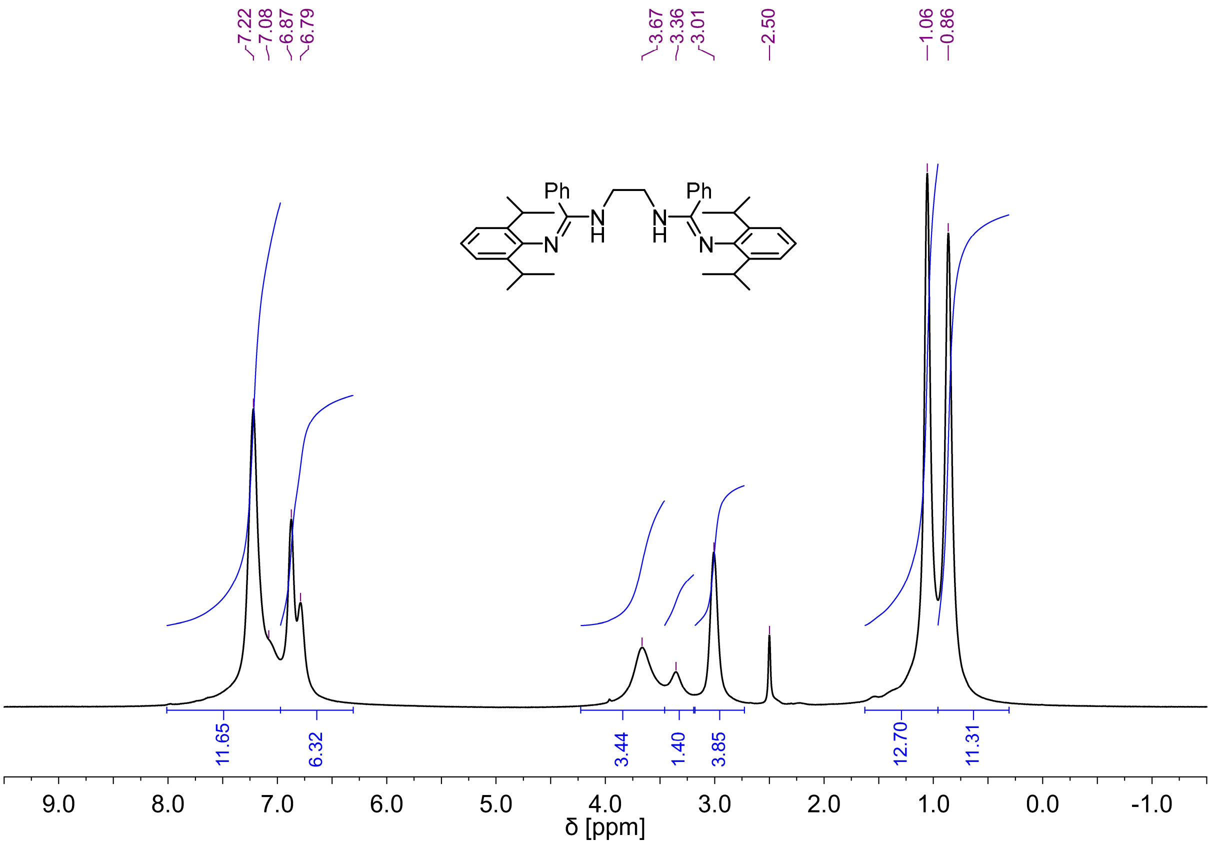

Figure S38: ^{1}H NMR spectrum of $\mathbf{L^1}$H$_2$ (DMSO-d_6, 400.1 MHz).

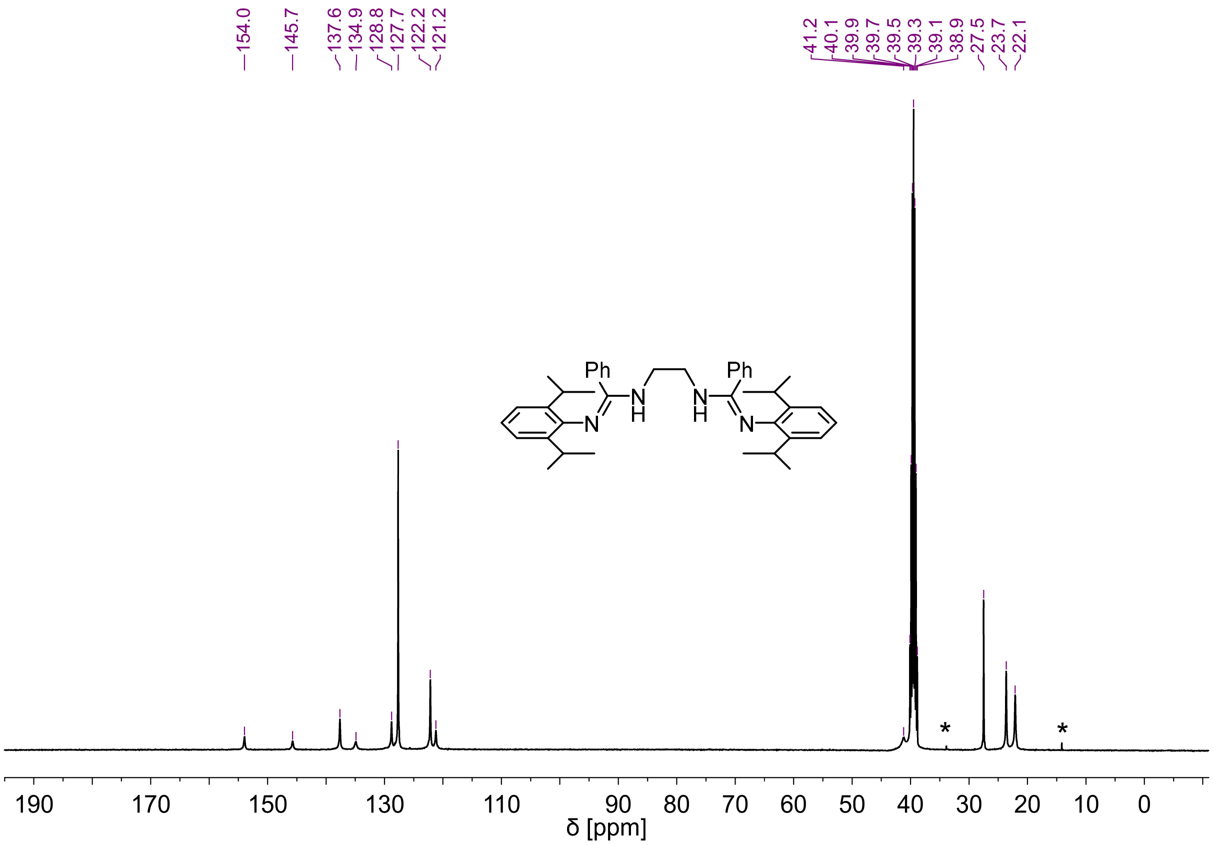

Figure S39: ^{13}C NMR spectrum of $\mathbf{L^1}$H$_2$ (DMSO-d_6, 100.6 MHz, *denotes residual hexanes).

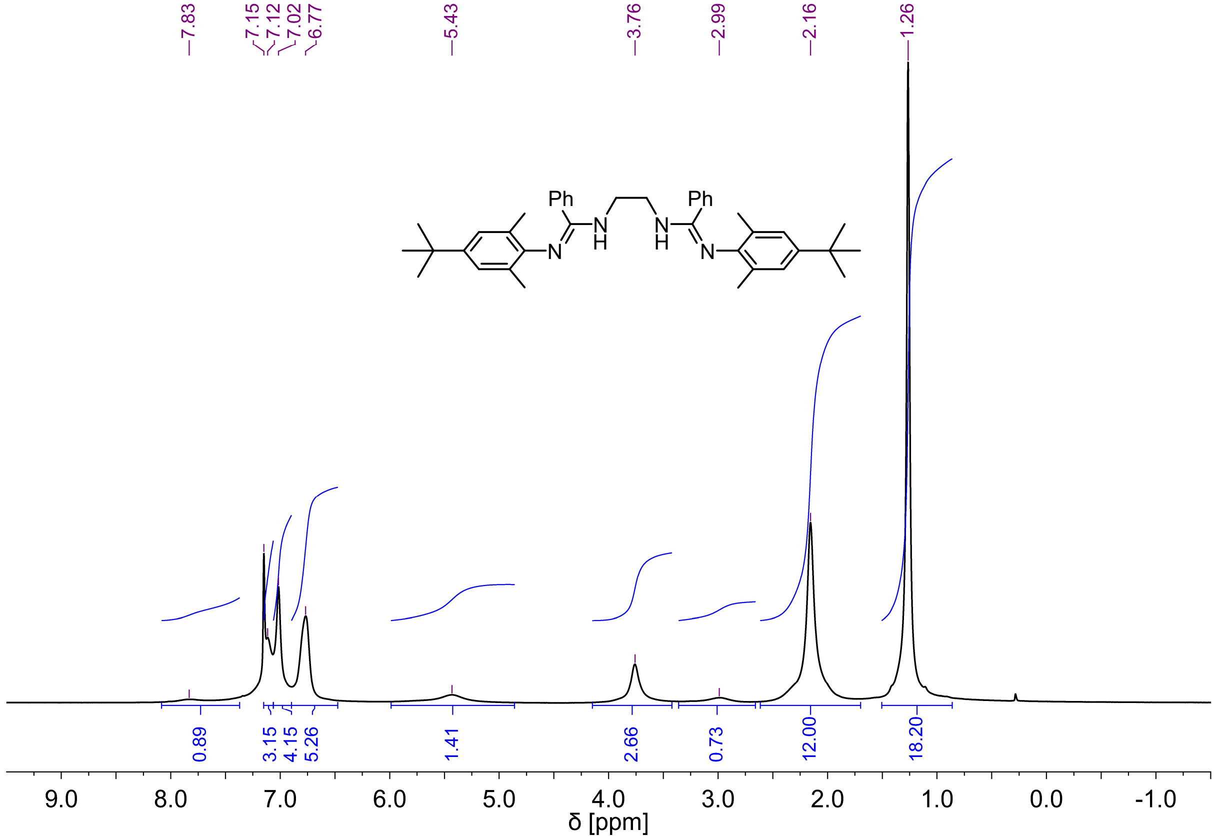

Figure S40: ^{1}H NMR spectrum of $\mathbf{L^2}H_2$ (C_6D_6, 400.1 MHz).

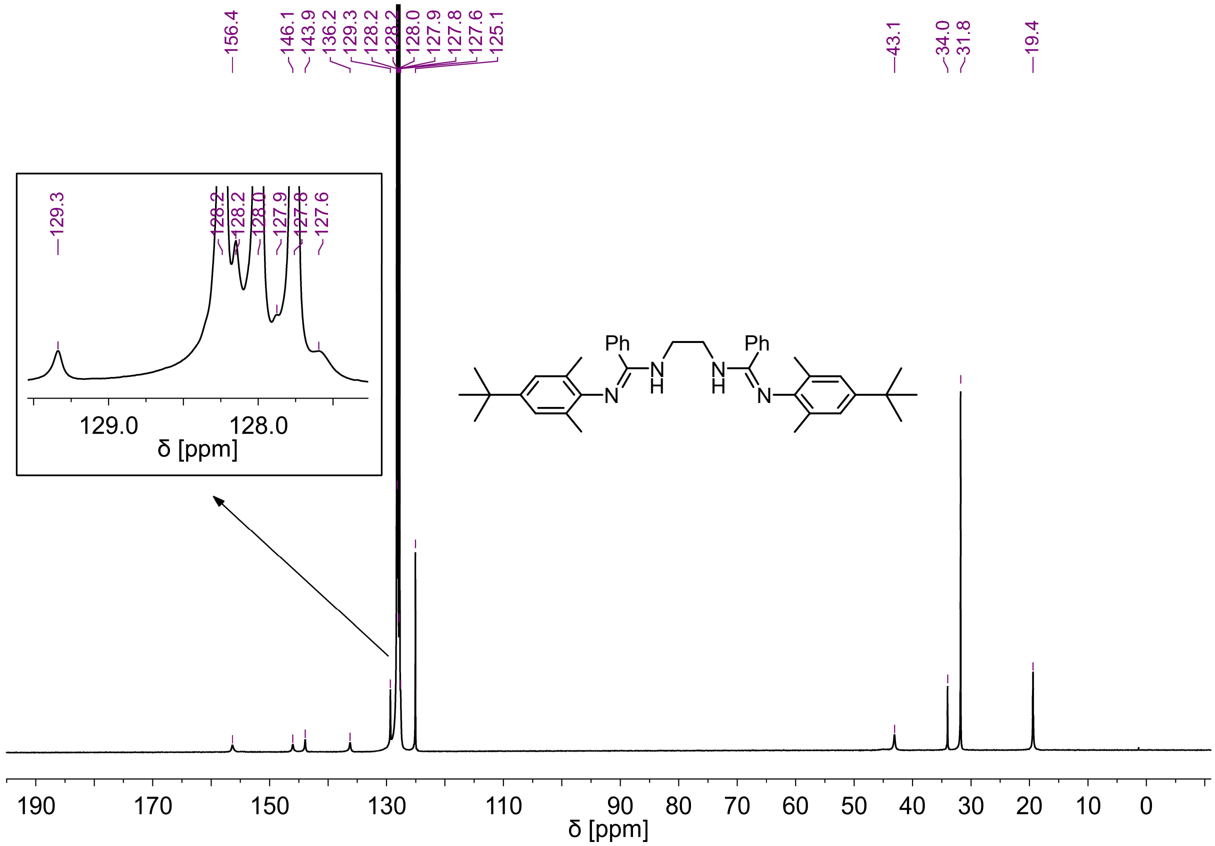

Figure S41: ^{13}C NMR spectrum of $\mathbf{L^2}H_2$ (C_6D_6, 100.6 MHz)

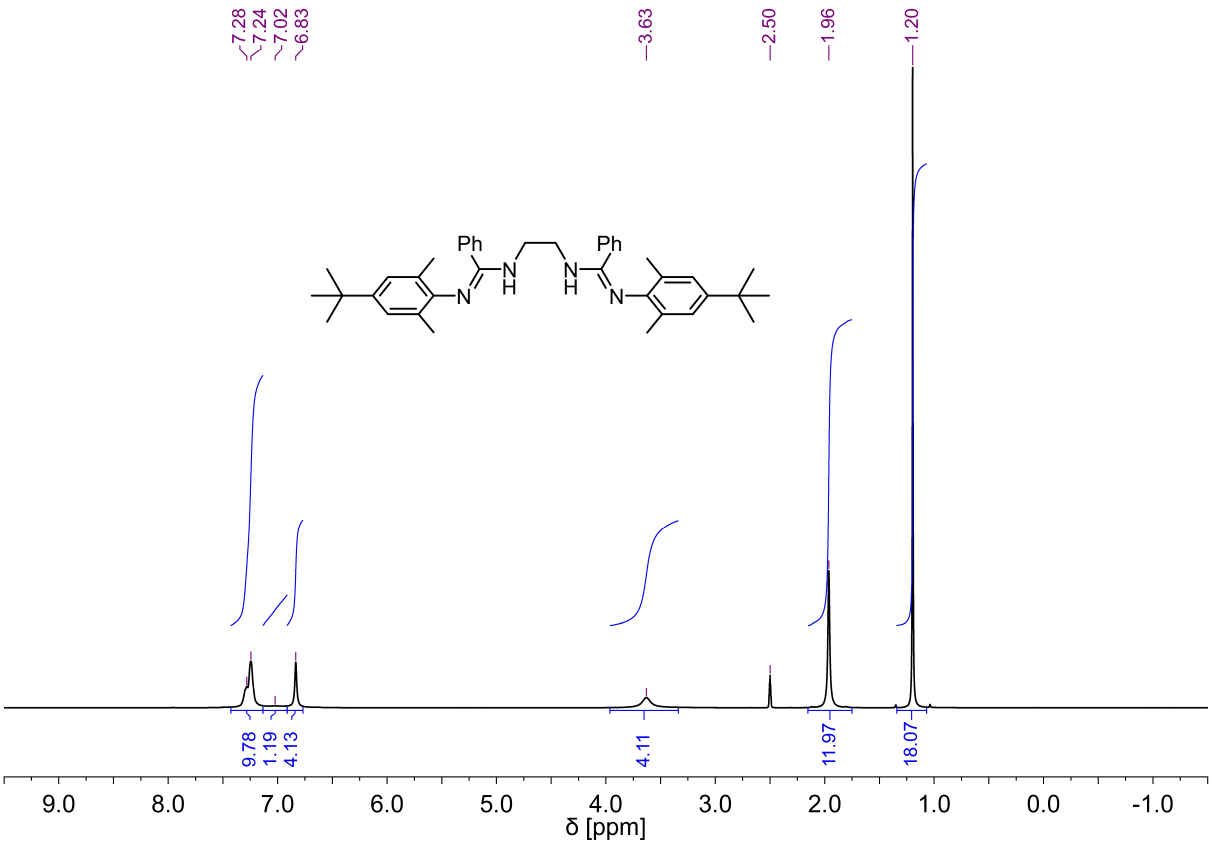

Figure S42: ^{1}H NMR spectrum of $\mathbf{L^2}H_2$ (DMSO-d_6, 400.1 MHz, 60 °C).

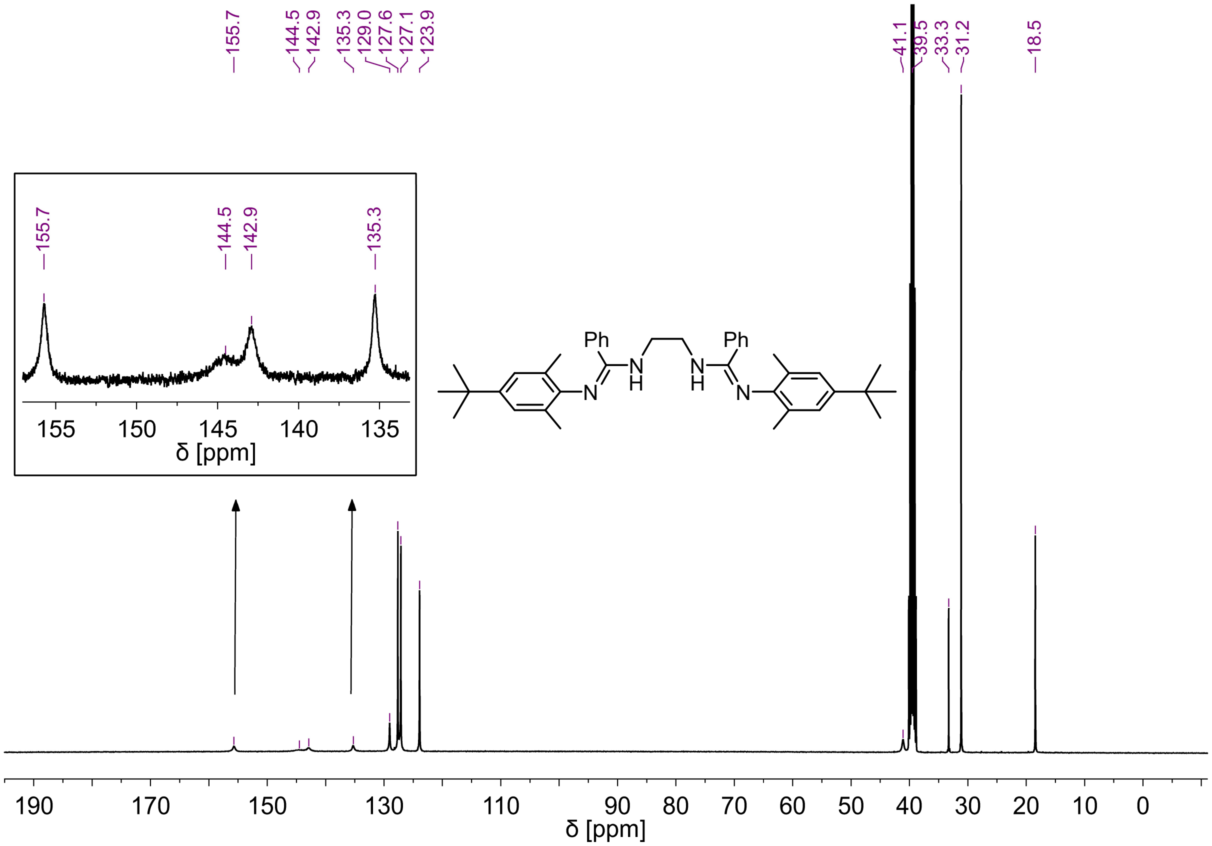

Figure S43: ^{13}C NMR spectrum of $\mathbf{L^2}H_2$ (DMSO-d_6, 100.6 MHz, 60 °C).

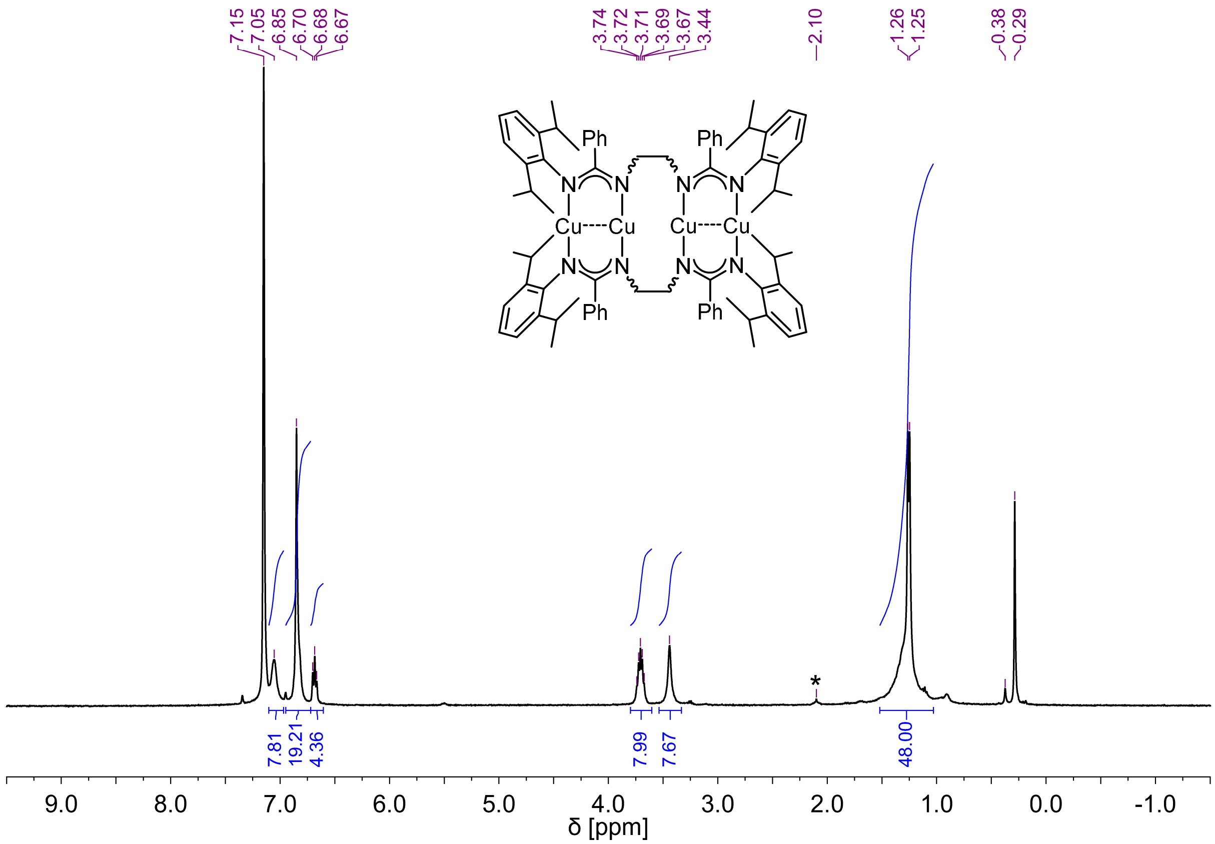

Figure S44: ^{1}H NMR spectrum of **1** (C_6D_6, 400.1 MHz, *denotes residual toluene).

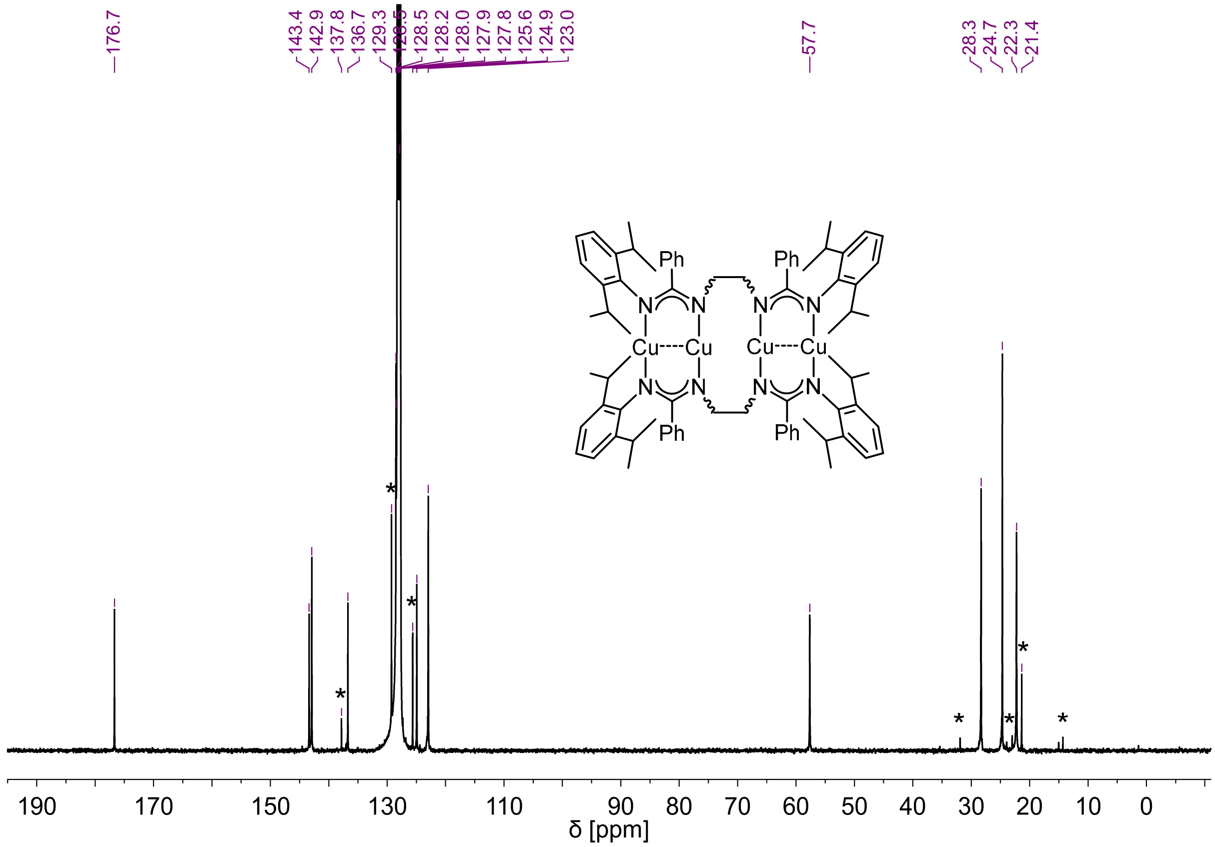

Figure S45: ^{13}C NMR spectrum of **1** (C_6D_6, 100.6 MHz, *denotes residual toluene and hexanes)

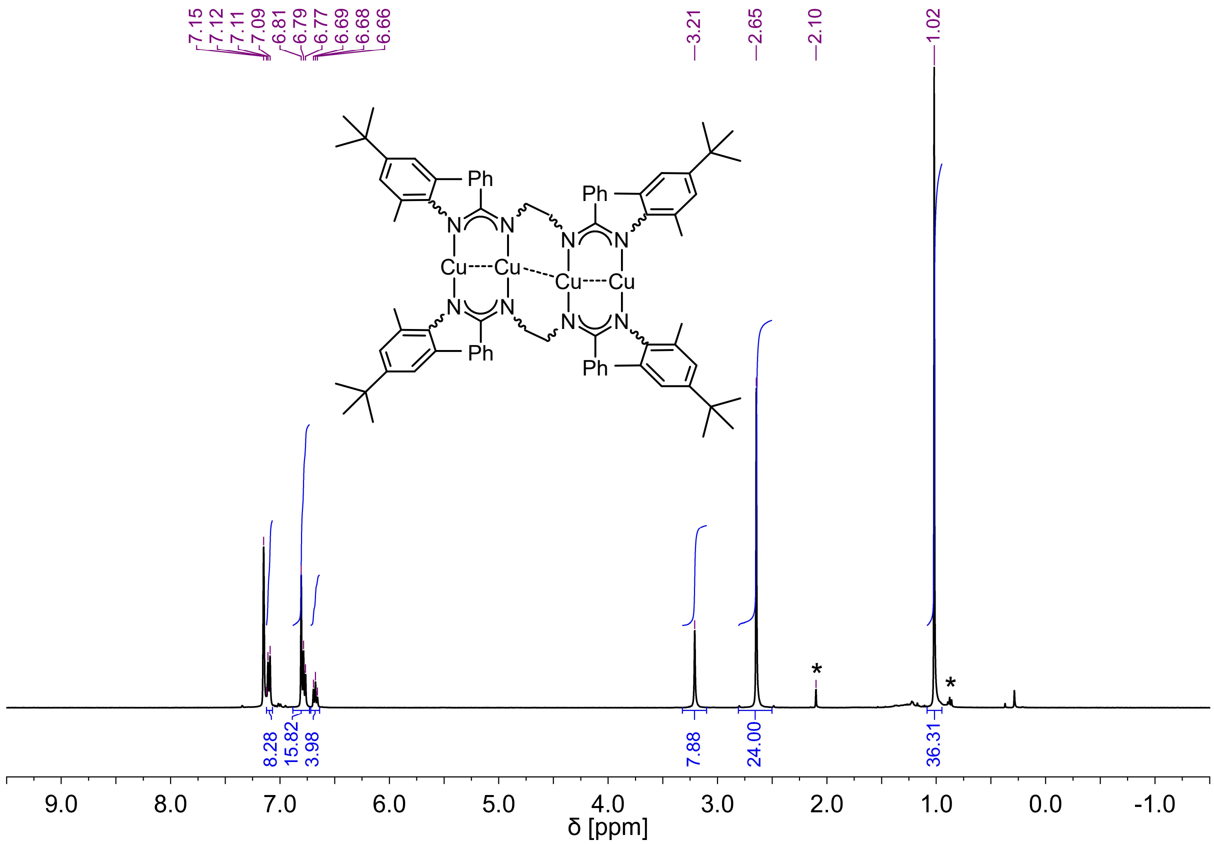

Figure S46: ^{1}H NMR spectrum of **2** (C_6D_6, 400.1 MHz, *denotes residual toluene and hexanes).

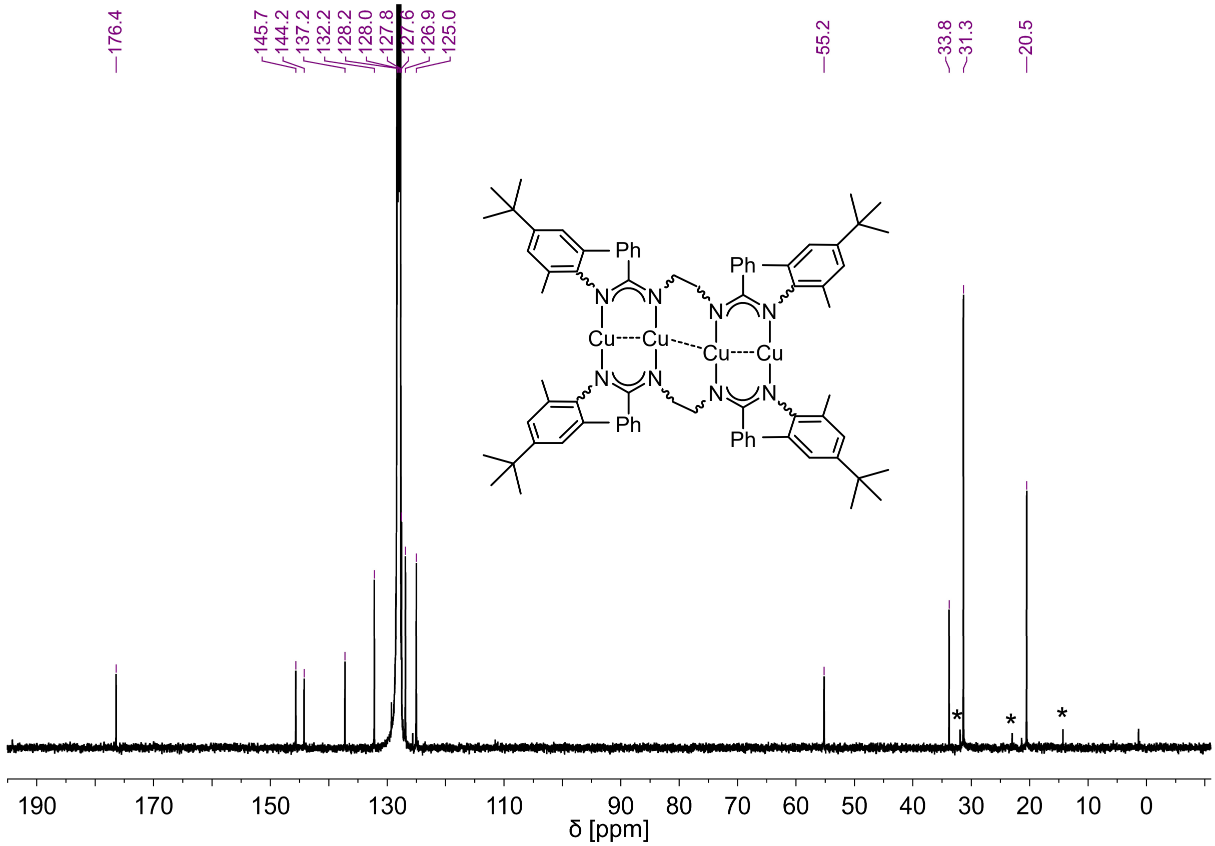

Figure S47: ^{13}C NMR spectrum of **2** (C_6D_6, 100.6 MHz, *denotes residual hexanes)

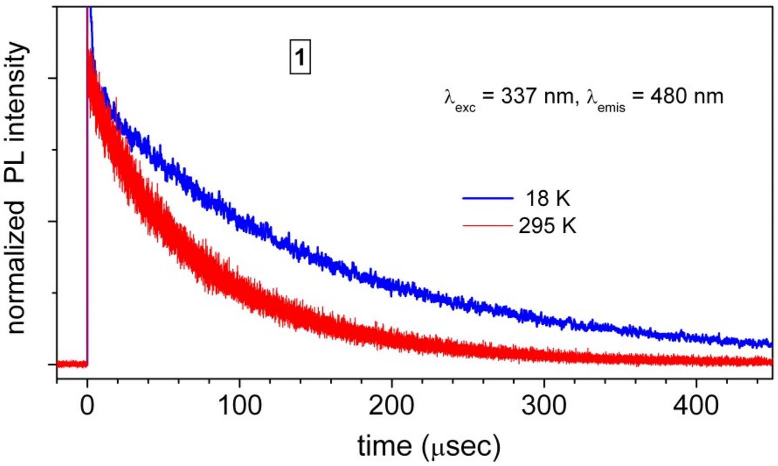

Figure S48. Emission decay traces of polycrystalline complex **1**·1.5C_7H_8 at 18 and 295 K under ns-pulsed excitation at 337 nm (N_2-laser). The decay can be fit with monoexponential curves, with τ = 161 and 74 μs, respectively.

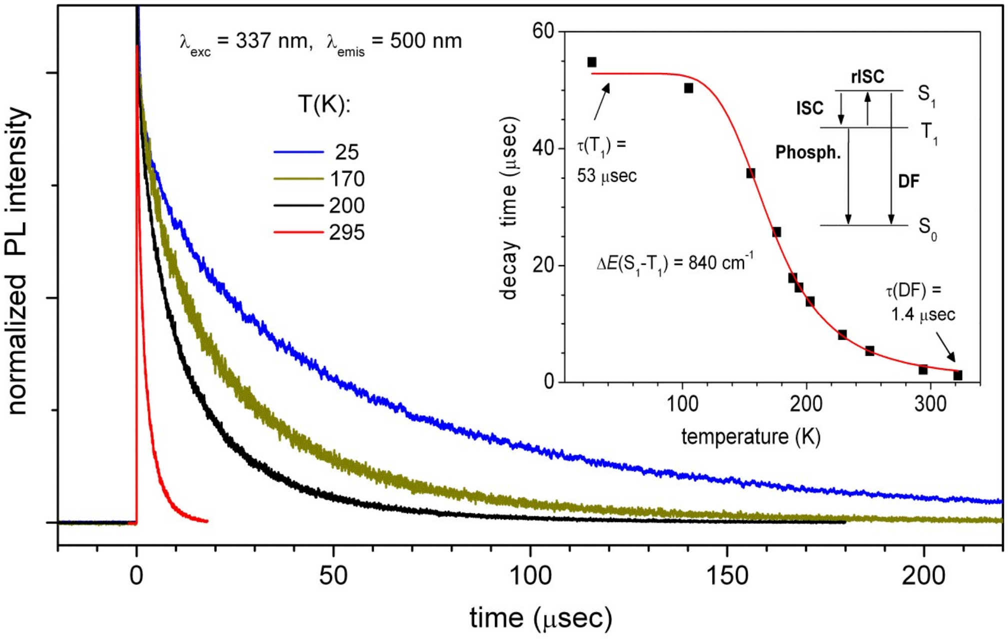

Figure S49. Emission decay traces of solid (polycrystalline) complex **2**·C_7H_8 at selected temperatures under N_2-laser ns-pulsed excitation at 337 nm. The decay can be well fit with biexponential curves, $I = A_1*\exp(-\tau_1/t) + A_2*\exp(-\tau_2/t)$, with relatively close values of τ_1 and τ_2. Monoexponential approximation can also be applied here, as this yields values quite similar to the average PL lifetimes from biexponential fits [$\tau_{aver} = (A_1*\tau_1 + A_2*\tau_2)/(A_1 + A_2)$]. The insert shows the average PL lifetime *vs*. temperature and a scheme of a delayed fluorescence (DF) process due to thermally activated reverse intersystem crossing (RISC) from T_1 to S_1 state. Accordingly, below T ~ 120 K, the emission of **2**·C_7H_8 is phosphorescence with the (average) lifetime of 53 μs. By increasing the temperature, it transforms to DF with the effective lifetime τ(DF) ≈ 1.4 μs at 325 K.

The energy separation ΔE between S_1 and T_1 states, $\Delta E(S_1–T_1)$, is estimated as 840 ± 50 cm^{-1} from the fit (red curve in the insert) of the experimental data to the simple TADF model of thermally equilibrated T_1 and S_1 states:[S7]

$$\tau(T) = \frac{3 + \exp\left(-\frac{\Delta E}{kT}\right)}{\frac{3}{\tau(\mathrm{T1})} + \frac{1}{\tau(\mathrm{S1})}\exp\left(-\frac{\Delta E}{kT}\right)} \qquad eq.\ 1$$

where τ(T) is the observed (effective) decay time and τ(T1) and τ(S1) are intrinsic lifetimes of the T_1 and S_1 states, and factor 3 accounts for three T_1 substates. The fit estimates τ(S1) as 16 ± 5 ns. Radiative lifetimes $\tau_r(T) = \tau(T) / \Phi_{PL}(T)$ would be more accurate input values in *eq*. 1, however, complex **2**·C_7H_8 only shows a very moderate temperature dependence of the emission efficiency $\Phi_{PL}(T)$ (as estimated from the temperature-dependent emission spectra, see Figure 2 in the main text), which could be neglected, in particular for the estimation of ΔE. The same remark applies to the fit in Figure S51.

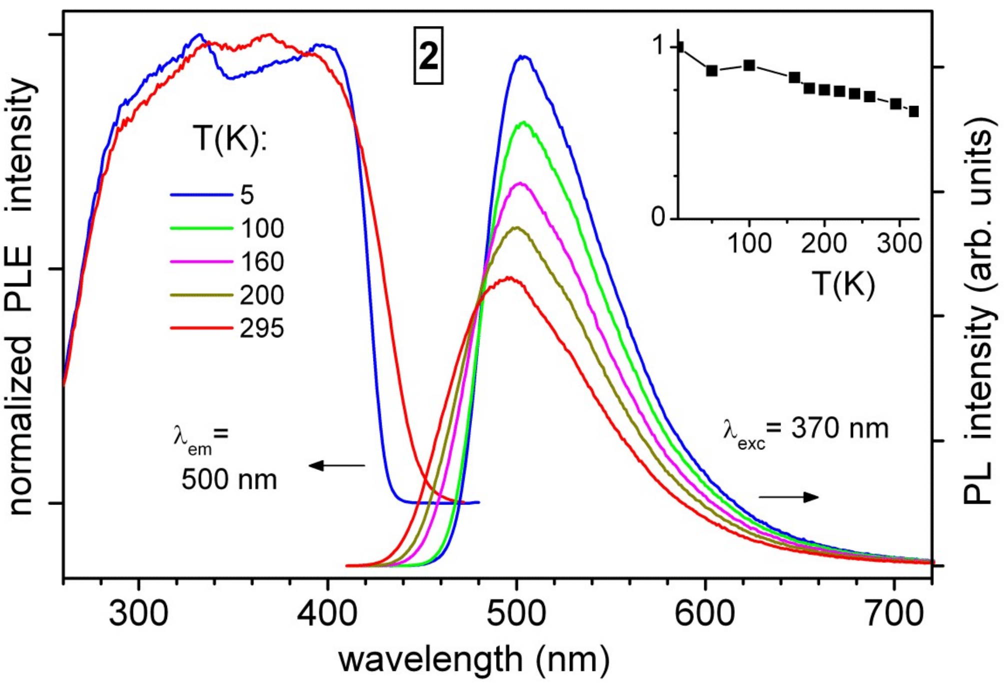

Figure S50. Temperature-dependent PL emission and excitation (PLE) spectra of solid (polycrystalline) complex **2** after vacuum drying (*cf*. Figure 2). The insert shows the integral PL intensity (normalized to unity) as a function of temperature. The emission features a high quantum yield of 67% at ambient temperature (determined using an integrating sphere and excitation at 400 nm), which approaches 100% below ~ 100 K according to the last plot.

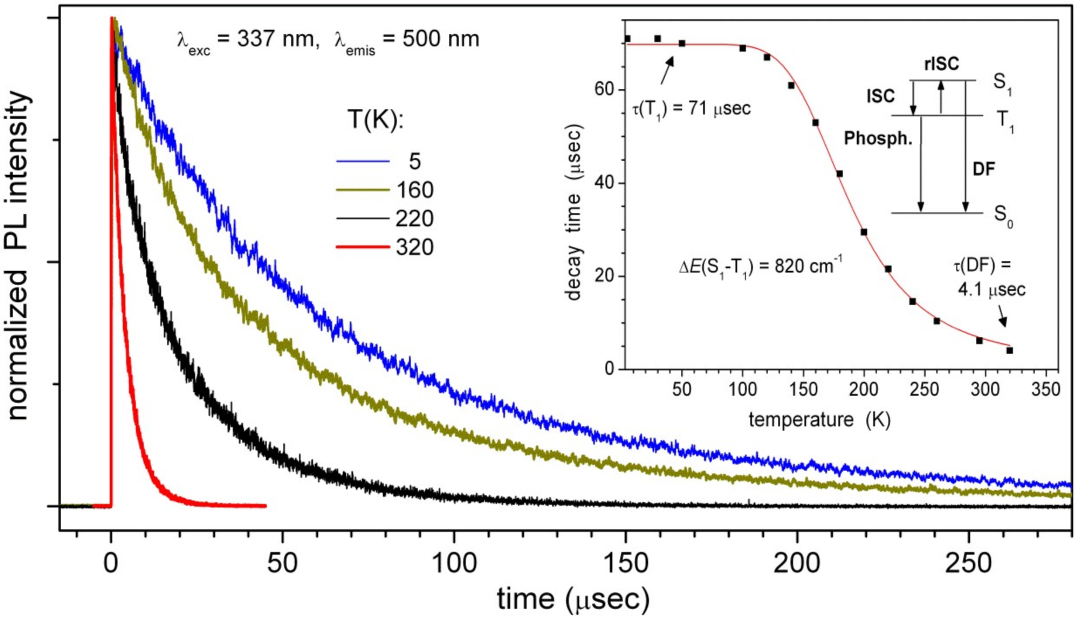

Figure S51. Temperature-dependent emission decay traces of polycrystalline complex **2** after vacuum drying (*cf*. Figure S49). The decay can be fit with biexponential curves, $I = A_1*\exp(-\tau_1/t) + A_2*\exp(-\tau_2/t)$, with relatively close values of τ_1 and τ_2. Monoexponential approximation can also be applied here, as this yields values quite similar to the average PL lifetimes from biexponential fits $[\tau_{aver} = (A_1*\tau_1 + A_2*\tau_2)/(A_1 + A_2)]$. The insert shows the average PL lifetime *vs*. temperature and a scheme of a thermally activated delayed fluorescence (DF) process. According to the simple TADF model (eq. 1 above, red curve in the insert), the energy separation ΔE between S_1 and T_1 states is estimated as 820 ± 30 cm^{-1}, *i.e.* with practically the same value as for $\mathbf{2}\cdot C_7H_8$ (Figure S49).

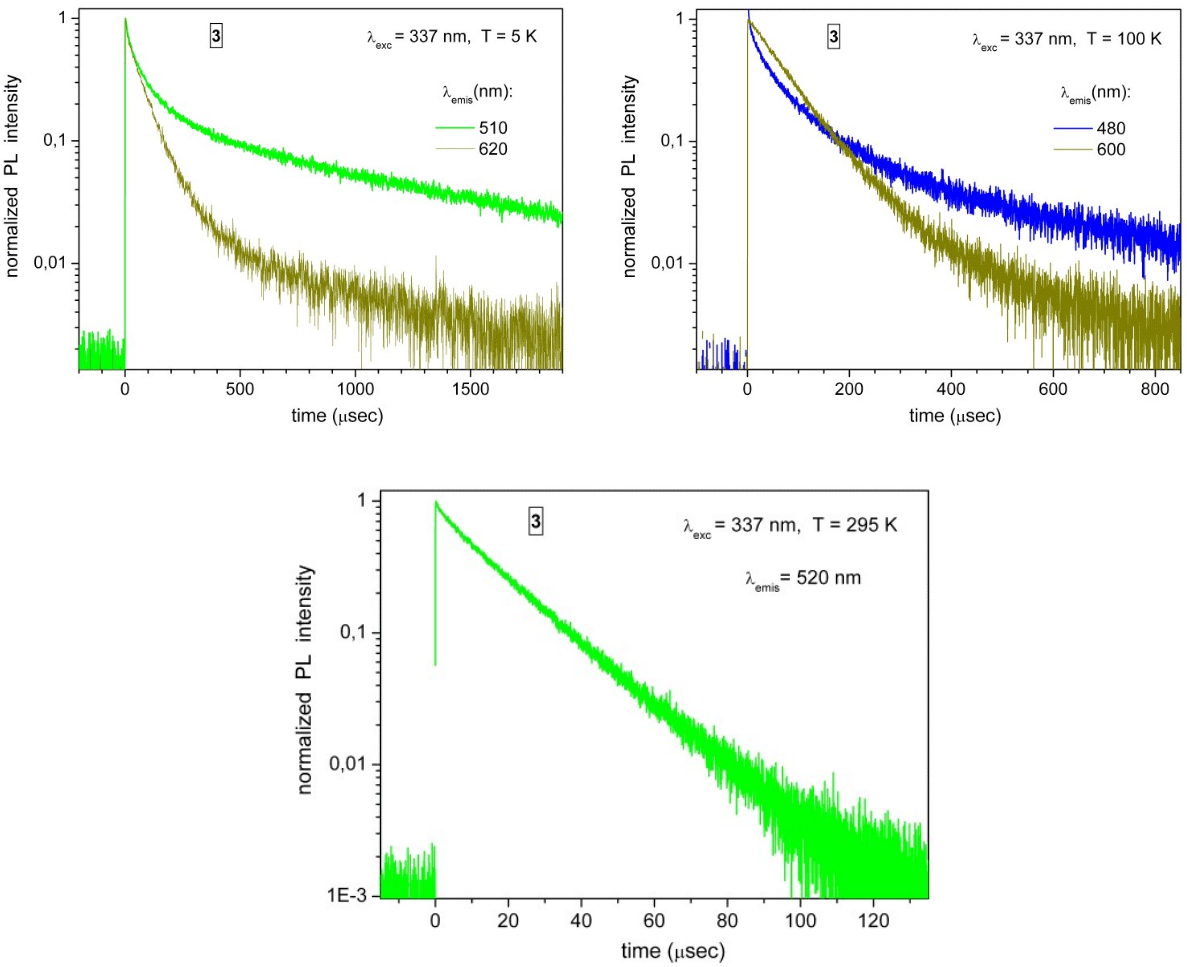

Figure S52. Emission decay traces of solid complex **3**·$5Et_2O$ at selected temperatures and excitation at 337 nm with a ns-pulsed N_2-laser. At ambient temperature the decay is monoexponential with $\tau = 16$ μs, whereas complicated kinetics is observed at low and intermediate (< 200 K) temperatures. At ~ 100–150 K (corresponding to the emission redshift, see main text), the decay also depends on the emission wavelength. Approximation with biexponential curves, $I = A*\exp(-\tau_1/t) + (1-A)*\exp(-\tau_2/t)$, yields following lifetimes (with relative weights of the two components in brackets):

5 K, λ_{emis} = 510 nm: $\tau_1 = 60$ μs (80%), $\tau_2 = 890$ μs (20%),

100 K, λ_{emis} = 480 nm: $\tau_1 = 40$ μs (78%), $\tau_2 = 250$ μs (22%),

100 K, λ_{emis} = 600 nm: $\tau_1 = 71$ μs (97%), $\tau_2 = 350$ μs (3%),

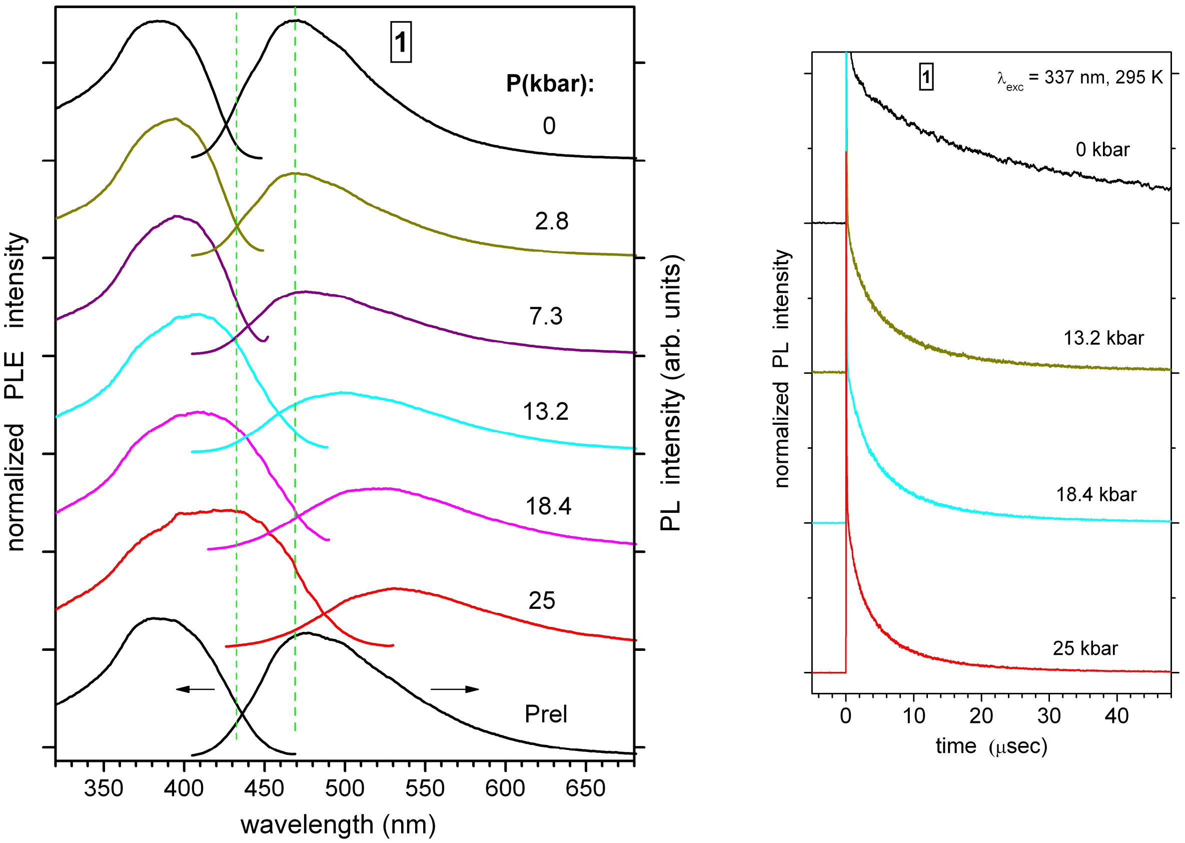

Figure S53. PL emission and excitation (PLE) spectra and decay traces of **1** at ambient temperature under high pressure in a diamond anvil cell (DAC). The PLE spectra are normalized, while the emission spectra are shown on the same scale (vertically shifted for clarity). PL decay was measured at 337 nm excitation with a ns-pulsed laser. The pressure was increased in steps up to 25 kbar and then released (Prel). All emission spectra were excited at 370 nm, the excitation spectra were recorded at 480–560 nm, following the emission redshift. The dashed vertical lines are drawn for convenience, to better follow pressure-induced spectral shifts. Note that both the emission and excitation spectra of **1** as well as the PL decay (not shown) are practically completely recovered after application of 25 kbar pressure.

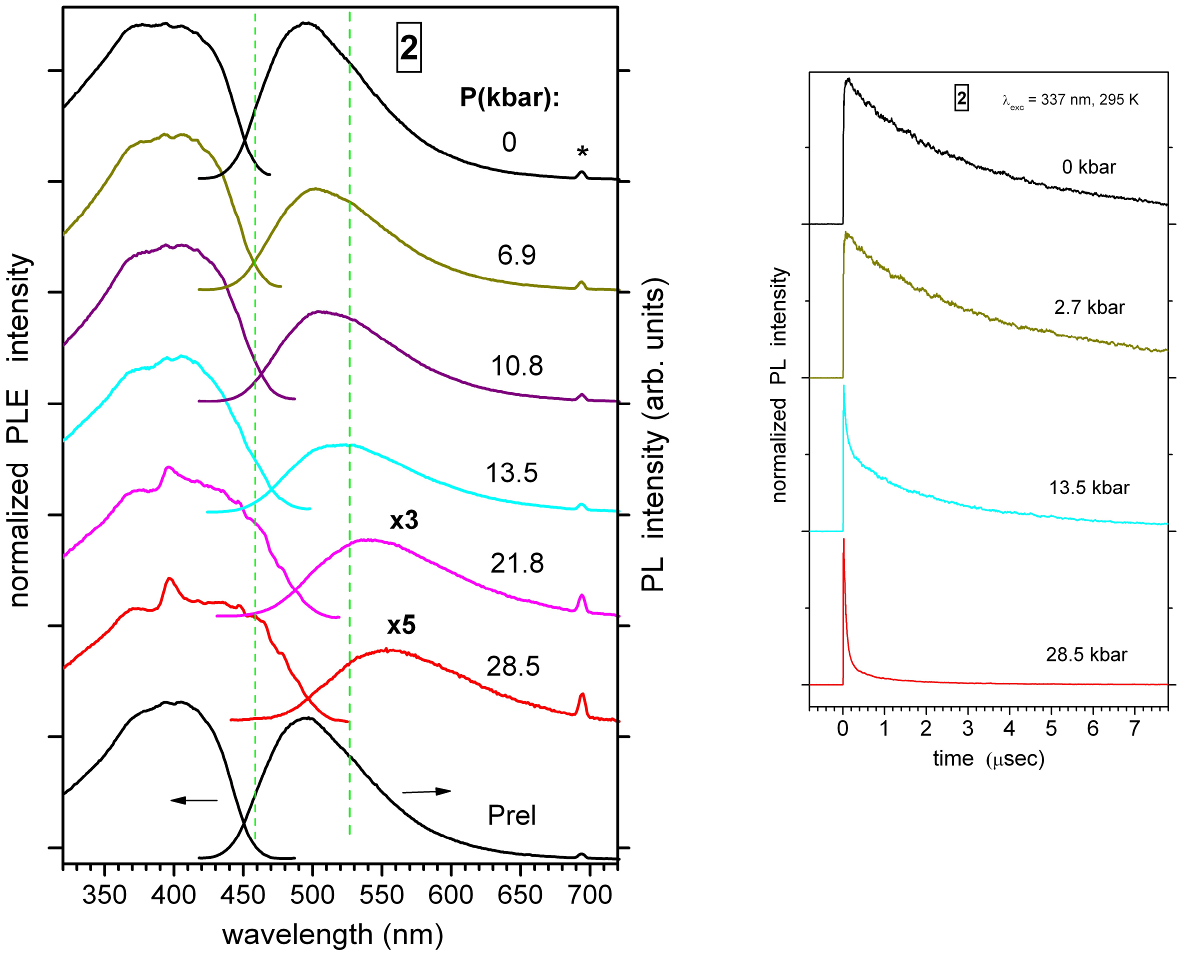

Figure S54. PL emission and excitation (PLE) spectra and decay traces of vacuum dried complex **2** at ambient temperature under high pressure in a diamond anvil cell (DAC). The PLE spectra are normalized, while the emission spectra are shown on the same scale (vertically shifted for clarity). The pressure was increased in steps up to 28.5 kbar and then released (Prel). All emission spectra were excited at 370 nm, the excitation spectra were recorded at 500–560 nm, following the emission redshift. The dashed vertical lines are drawn for convenience, to better follow pressure-induced spectral shifts. PL decay was measured at 337 nm excitation with a ns-pulsed laser. Note that both the emission and excitation spectra as well as the PL decay (not shown) are practically completely recovered after application of 28 kbar pressure. The asterisk indicates a ruby emission at *ca*. 694 nm used a pressure sensor.

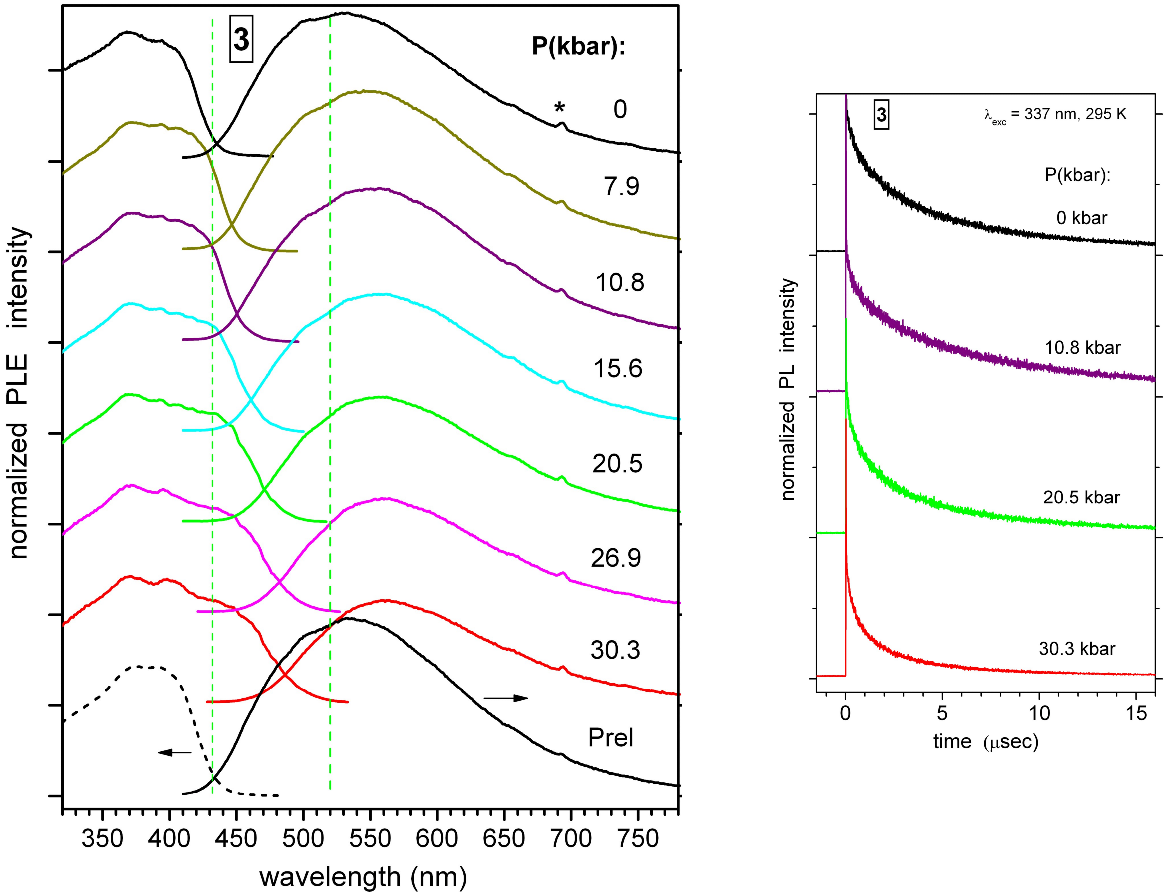

Figure S55. PL emission and excitation (PLE) spectra and decay traces of **3**·5Et_2O at ambient temperature under high pressure in a diamond anvil cell (DAC). The PLE spectra are normalized, while the emission spectra are shown on the same scale (vertically shifted for clarity). PL decay was measured at 337 nm excitation with a ns-pulsed laser. The pressure was increased in steps up to 30.3 kbar and then released (Prel). All emission spectra were excited at 370 nm, the excitation spectra were recorded at 530–580 nm, following the emission redshift. The dashed vertical lines are drawn for convenience, to better follow pressure-induced spectral shifts. The asterisk indicates a ruby (pressure sensor) emission at *ca*. 694 nm. Similar to **1** and **2** (Figures S53, S54), the PL spectra of **3** as well as its PL decay (not shown) are practically completely recovered after application of 30 kbar pressure.

Table S5. Photophysical parameters for polycrystalline complexes **1**·1.5C_7H_8, **2**·C_7H_8, and **3**·5Et_2O.

	**1**·1.5C_7H_8	**2**·C_7H_8	**3**·5Et_2O
Onset of absorption [nm] at 20/295 K [a]	410/430	435/460	430/445
Emission maximum [nm] at 20/ 295 K	461[b], 493[b]/467	495/472	507/525
PL quantum yield [%] (T = 295 K, λ_{exc} = 400 nm)	6.5	17	12.5
Estimated quantum yield at 20 K [%]	23	24	20
Emission lifetime [μs] at 20/ 295 K (ns-laser excitation at λ_{exc}= 337 nm)	160/74	55[c]/2.1[c]	230[c]/16

[a]) determined from PL excitation spectra, [b]) vibronic bands in emission spectrum

[c]) average value from biexponential fit

References and Footnotes

(S1) Except for Fig. S19, graphics were generated using the following program: DIAMOND—Crystal and Molecular Structure Visualization (version 3.2k), CRYSTAL IMPACT, Dr. H. Putz and Dr. K. Brandenburg GbR, Kreuzherrenstr. 102, 53227 Bonn (Germany).

(S2) These torsion angles were calculated with the program DIAMOND 3.2k.

(S3) The analyses was performed using the program Mercury 2023.1.0: Macrae, C. F.; Sovago, I.; Cottrell, S. J.; Galek, P. T. A.; McCabe, P.; Pidcock, E.; Platings, M.; Shields, G. P.; Stevens, J. S.; Towler, M.; Wood, P. A. *J. Appl. Cryst.* **2020**, *53*, 226–235. The calculations are based on the contact surfaces and the approximate grid spacing for the spherical probes was 0.1 Å.

(S4) Stollenz, M.; Raymond, J. E.; Peréz, L. M.; Jessica Wiederkehr, J.; Bhuvanesh, N. *Chem. Eur. J.* **2016**, *22*, 2396–2405.

(S5) Graphic was generated using the following program: UCSF CHIMERA – A Visualization System for Exploratory Research and Analysis, Pettersen, E. F.; Goddard, T. D.; Huang, C. C.; Couch, G. S.; Greenblatt, D. M.; Meng, E. C.; Ferrin, T. E. *J. Comput. Chem.* **2004**, 25, 1605–1612.

(S6) Graphic was generated using the following program: VMD – Visual Molecular Dynamics, Humphrey, W.; Dalke, A.; Schulten, K., *J. Molec. Graphics* **1996**, *14*, 33–38.

(S7) see, e. g., Leitl, M. J.; Kuchle, F. R.; Mayer, H. A.; Wesemann, L.; Yersin, H. *J. Phys. Chem. A* **2013**, *117*, 11823–11836.
